# Supplementary figures and images for: Defining predictors of responsiveness to advanced therapies in Crohn’s disease and ulcerative colitis: protocol for the IBD-RESPONSE and nested CD-metaRESPONSE prospective, multicentre, observational cohort study in precision medicine
Source: BMJ Open. 2024 Apr 17;14(4):e073639. doi: 10.1136/bmjopen-2023-073639 (PMC11029295; doi:10.1136/bmjopen-2023-073639)

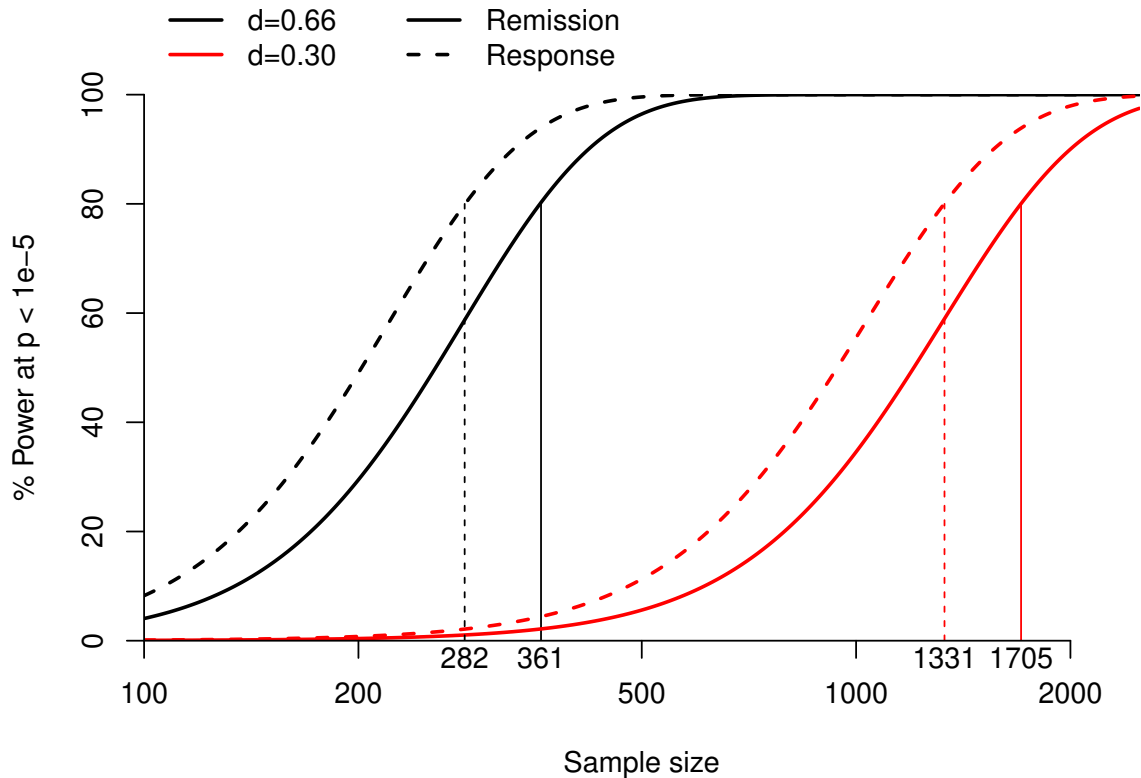

Supplement: Supplementary data [file bmjopen-2023-073639supp001.pdf]
