# Supplementary material for: Defining predictors of responsiveness to advanced therapies in Crohn’s disease and ulcerative colitis: protocol for the IBD-RESPONSE and nested CD-metaRESPONSE prospective, multicentre, observational cohort study in precision medicine
Source: BMJ Open. 2024 Apr 17;14(4):e073639. doi: 10.1136/bmjopen-2023-073639 (PMC11029295; doi:10.1136/bmjopen-2023-073639)
Supplement: Author's manuscript [file bmjopen-2023-073639.draft_revisions.pdf]

BMJ Open is committed to open peer review. As part of this commitment we make the peer review history of every article we publish publicly available.

When an article is published we post the peer reviewers' comments and the authors' responses online. We also post the versions of the paper that were used during peer review. These are the versions that the peer review comments apply to.

The versions of the paper that follow are the versions that were submitted during the peer review process. They are not the versions of record or the final published versions. They should not be cited or distributed as the published version of this manuscript.

BMJ Open is an open access journal and the full, final, typeset and author-corrected version of record of the manuscript is available on our site with no access controls, subscription charges or pay-per-view fees (http://bmjopen.bmj.com).

If you have any questions on BMJ Open's open peer review process please email info.bmjopen@bmj.com

BMJ Open

Defining predictors of responsiveness to advanced therapies in Crohn's disease and ulcerative colitis: Protocol for the IBD-RESPONSE and nested CD-metaRESPONSE prospective, multicentre, observational cohort study in precision medicine

Journal:	*BMJ Open*
Manuscript ID	bmjopen-2023-073639
Article Type:	Protocol
Date Submitted by the Author:	15-Mar-2023
Complete List of Authors:	Wyatt, Nicola; Newcastle University Translational and Clinical Research Institute, Faculty of Medical Sciences; Newcastle Upon Tyne Hospitals NHS Foundation Trust, Department of Gastroenterology<br>Fuller, Hannah; Newcastle University Translational and Clinical Research Institute, Faculty of Medical Sciences<br>Anderson, Carl ; Wellcome Sanger Institute, Wellcome Genome Campus<br>Kennedy, Nicholas A; Royal Devon University Healthcare NHS Foundation Trust, Department of Gastroenterology; University of Exeter Inflammatory Bowel Disease and Pharmacogenetics Research Group<br>Raine, Tim; Cambridge University Hospitals NHS Foundation Trust<br>Ahmad, T.; Royal Devon University Healthcare NHS Foundation Trust, Department of Gastroenterology; University of Exeter Inflammatory Bowel Disease and Pharmacogenetics Research Group<br>Allerton, Dean; Newcastle University, Newcastle Clinical Trials Unit<br>Bardgett, Michelle; Newcastle University, Newcastle Clinical Trials Unit<br>Clark, Emma; Newcastle University, Newcastle Clinical Trials Unit<br>Clewes, Dawn; Newcastle University, Newcastle Clinical Trials Unit<br>Cotobal Martin, Cristina; Wellcome Sanger Institute, Wellcome Genome Campus<br>Doona, Mary; Newcastle Upon Tyne Hospitals NHS Foundation Trust, Department of Gastroenterology<br>Doyle, Jennifer; Newcastle University Translational and Clinical Research Institute, Faculty of Medical Sciences<br>Hancock, Helen; Newcastle University, Newcastle Clinical Trials Unit<br>Hart, Ailsa L.; St Mark's Hospital and Academic Institute, Department of Gastroenterology; Imperial College London, Department of Surgery and Cancer<br>Irving, Peter; Guy's and St Thomas' NHS Foundation Trust, Department of Gastroenterology; King's College London, School of Immunology & Microbial Sciences<br>Iqbal, Sameena; Wellcome Sanger Institute, Wellcome Genome Campus<br>Kennedy, Ciara; Newcastle University, Newcastle Clinical Trials Unit<br>King, Andrew; Newcastle Upon Tyne Hospitals NHS Foundation Trust, Department of Gastroenterology<br>Lawrence, Sarah; Newcastle University, Newcastle Clinical Trials Unit<br>Lees, Charlie W.; The University of Edinburgh MRC Institute of Genetics and Molecular Medicine; Western General Hospital, Institute of Genetics

& Molecular Medicine
Lees, Robert; Newcastle Upon Tyne Hospitals NHS Foundation Trust, Department of Gastroenterology
Letchford, Laura; Wellcome Sanger Institute, Wellcome Genome Campus
Liddle, Trevor; Newcastle Upon Tyne Hospitals NHS Foundation Trust
Lindsay, James ; Barts Health NHS Trust, Department of Gastroenterology; Blizard Institute of Cell and Molecular Science, Centre for Immunobiology
Maier, Rebecca; Newcastle University, Newcastle Clinical Trials Unit
Mansfield, John; Newcastle University Translational and Clinical Research Institute, Faculty of Medical Sciences; Newcastle Upon Tyne Hospitals NHS Foundation Trust, Department of Gastroenterology
Marchesi, Julian R.; St Mary's Hospital, Division of Digestive Diseases, Department of Metabolism, Digestion and Reproduction
McGregor, Naomi; Newcastle University, Newcastle Clinical Trials Unit
McIntyre, Rebecca ; Wellcome Sanger Institute, Wellcome Genome Campus
Ostermayer, Jasmin; Wellcome Sanger Institute, Wellcome Genome Campus
Osunnuyi, Tolulope; Wellcome Sanger Institute, Wellcome Genome Campus
Powell, Nick; St Mary's Hospital, Division of Digestive Diseases, Department of Metabolism, Digestion and Reproduction; Imperial College Healthcare NHS Trust, Division of Digestive Diseases, Department of Metabolism, Digestion and Reproduction
Prescott, Natalie; King's College London, Department of Medical and Molecular Genetics, Guy's Hospital
Satsangi, J; University of Oxford, Nuffield Department of Medicine
Sharma, Shriya; Newcastle University, Newcastle Clinical Trials Unit
Shrestha, Tara; Newcastle University, Newcastle Clinical Trials Unit
Speight, Ally; Newcastle University Translational and Clinical Research Institute; Newcastle Upon Tyne Hospitals NHS Foundation Trust, Department of Gastroenterology
Strickland, Michelle; Wellcome Sanger Institute, Wellcome Genome Campus
Wason, James; Newcastle University, Population Health Sciences Institute
Whelan, Kevin; King's College London, Department of Nutritional Sciences
Wood, Ruth; Newcastle University, Newcastle Clinical Trials Unit
Zhang, Xinyue; Newcastle University, Population Health Sciences Institute
Parkes, M; Cambridge University Hospitals NHS Foundation Trust, Department of Gastroenterology
Stewart, Christopher; Newcastle University Translational and Clinical Research Institute, Faculty of Medical Sciences
Jostins-Dean, Luke; University of Oxford, Kennedy Institute of Rheumatology
Lamb, Christopher; Newcastle University Translational and Clinical Research Institute, Faculty of Medical Sciences; Newcastle Upon Tyne Hospitals NHS Foundation Trust, Department of Gastroenterology

Keywords: Inflammatory bowel disease < GASTROENTEROLOGY, Gastroenterology < INTERNAL MEDICINE, MICROBIOLOGY, Patient-Centered Care, Patient Reported Outcome Measures

SCHOLARONE™
Manuscripts

Defining predictors of responsiveness to advanced therapies in Crohn's disease and ulcerative colitis: Protocol for the IBD-RESPONSE and nested CD-metaRESPONSE prospective, multicentre, observational cohort study in precision medicine

Nicola J. Wyatt[1,2], Hannah Fuller[1], Carl A. Anderson[3], Nicholas A. Kennedy[4,5], Tim Raine[6], Tariq Ahmad[4,5], Dean Allerton[7], Michelle Bardgett[7], Emma Clark[7], Dawn Clewes[7], Cristina Cotobal Martin[3], Mary Doona[2], Jennifer A. Doyle[1], Helen Hancock[7], Ailsa Hart[8,9], Peter M. Irving[10,11], Sameena Iqbal[3], Ciara Kennedy[7], Andrew King[2], Sarah Lawrence[7], Charlie W. Lees[12,13], Robert Lees[2], Laura Letchford[3], Trevor Liddle[14], James O. Lindsay[15,16], Rebecca H. Maier[7], John C. Mansfield[1,2], Julian R. Marchesi[17], Naomi McGregor[7], Rebecca E. McIntyre[3], Jasmin Ostermayer[3], Tolulope Osunnuyi[3], Nick Powell[17,18], Natalie J Prescott[19], Jack Satsangi[20], Shriya Sharma[7], Tara Shrestha[7], R. Alexander Speight[1,2], Michelle Strickland[3], James M. S. Wason[21], Kevin Whelan[22], Ruth Wood[7], Xinyue Zhang[21], Miles Parkes[6], Christopher J. Stewart[*1], Luke Jostins-Dean[*23], Christopher A. Lamb[*1,2]

[*]Denotes co-senior authorship

Correspondence:

Dr Christopher Lamb, Translational & Clinical Research Institute, Faculty of Medical Sciences, Newcastle University, Newcastle upon Tyne, NE2 4HH. United Kingdom

christopher.lamb@newcastle.ac.uk

For peer review only

Affiliations:

1. Translational & Clinical Research Institute, Faculty of Medical Sciences, Newcastle University, Newcastle upon Tyne, United Kingdom
2. Department of Gastroenterology, The Newcastle upon Tyne Hospitals NHS Foundation Trust, Newcastle upon Tyne, United Kingdom
3. Wellcome Sanger Institute, Wellcome Genome Campus, Hinxton, Cambridgeshire, United Kingdom
4. Department of Gastroenterology, Royal Devon University Healthcare NHS Foundation Trust, Exeter, United Kingdom
5. Exeter Inflammatory Bowel Disease and Pharmacogenetics Research Group, University of Exeter, Exeter, United Kingdom
6. Cambridge University Hospitals NHS Foundation Trust, Cambridge, United Kingdom
7. Newcastle Clinical Trials Unit (NCTU), 1-4 Claremont Terrace, Newcastle University, Newcastle upon Tyne, NE2 4AE, United Kingdom
8. Department of Gastroenterology, St Marks Hospital and Academic Institute, Gastroenterology, London, United Kingdom
9. Department of Surgery and Cancer, Imperial College, London, United Kingdom
10. Department of Gastroenterology, Guy's & St Thomas' NHS Foundation Trust, London, United Kingdom
11. School of Immunology & Microbial Sciences, King's College London, London, United Kingdom
12. Institute of Genetics & Molecular Medicine, University of Edinburgh, Edinburgh, United Kingdom
13. Edinburgh IBD Unit, Western General Hospital, NHS Lothian, Edinburgh, United Kingdom

For peer review only

14. The Newcastle upon Tyne Hospitals NHS Foundation Trust, Newcastle upon Tyne. United Kingdom

15. Department of Gastroenterology, Barts Health NHS Trust, The Royal London Hospital, London, United Kingdom

16. Centre for Immunobiology, Blizard Institute, Barts and the London School of Medicine Queen Mary University of London, London, United Kingdom

17. Division of Digestive Diseases, Department of Metabolism, Digestion and Reproduction, St Mary's Hospital, Imperial College London, London, United Kingdom

18. Department of Gastroenterology, Imperial College Healthcare NHS Trust, London, United Kingdom

19. Department of Medical and Molecular Genetics, King's College London, Guy's Hospital, London, United Kingdom

20. Nuffield Department of Medicine, University of Oxford, Old Road Campus, Oxford, United Kingdom

Department of Nutritional Sciences, King's College London, London, United Kingdom

21. Population Health Sciences Institute, Newcastle University, Newcastle upon Tyne, United Kingdom

22. Department of Nutritional Sciences, King's College London, United Kingdom

23. Kennedy Institute of Rheumatology, University of Oxford, Oxford, United Kingdom

For peer review only

Keywords:

Inflammatory bowel disease, Crohn disease, gastrointestinal microbiome, multiomics, precision medicine

ORCiDs (alphabetical):

For peer review only - http://bmjopen.bmj.com/site/about/guidelines.xhtml

Tariq Ahmad: https://orcid.org/0000-0002-6058-5528

Dean Allerton: https://orcid.org/0000-0003-0507-4343

Carl A. Anderson: https://orcid.org/0000-0003-1719-7009

Michelle Bardgett: https://orcid.org/0000-0003-1393-2010

Emma Clark: https://orcid.org/0000-0003-0065-1463

Dawn Clewes: https://orcid.org/0000-0001-9600-859X

Cristina Cotobal-Martin: https://orcid.org/0000-0002-5877-2228

Mary Doona: https://orcid.org/0000-0001-9533-3745

Jennifer A. Doyle: https://orcid.org/0000-0001-7042-3269

Hannah Fuller: https://orcid.org/0000-0002-7965-938X

Helen Hancock: https://orcid.org/0000-0002-1494-8551

Ailsa Hart: https://orcid.org/0000-0002-7141-6076

Peter M. Irving: https://orcid.org/0000-0003-0972-8148

Sameena Iqbal: https;//orcid.org/0000-0003-0926-593X

Luke Jostins-Dean: https://orcid.org/0000-0002-2475-3969

Ciara Kennedy: https://orcid.org/0000-0003-2987-0977

Nicholas A. Kennedy: https://orcid.org/0000-0003-4368-1961

Andrew King: https://orcid.org/0000-0002-5002-4751

Christopher A. Lamb: https://orcid.org/0000-0002-7271-4956

Sarah Lawrence: n/a

Charlie W. Lees: https://orcid.org/0000-0002-0732-8215

Robert Lees: n/a

Laura Letchford: n/a

Trevor Liddle: n/a

James O. Lindsay: https://orcid.org/0000-0003-3353-9590

Rebecca H. Maier: https://orcid.org/0000-0002-7350-3288

John C. Mansfield: https://orcid.org/0000-0003-2490-7750

Julian R. Marchesi: https://orcid.org/0000-0002-7994-5239

Naomi McGregor: https://orcid.org/0000-0002-5961-124X

Rebecca E. McIntyre: https://orcid.org/0000-0001-5291-1533

Jasmin Ostermayer: https://orcid.org/0000-0001-6461-398X

For peer review only

Tolulope Osunnuyi: https://orcid.org/0000-0002-0482-917X

Miles Parkes: https://orcid.org/0000-0002-6467-0631

Nick Powell: https://orcid.org/0000-0003-3231-6950

Natalie J. Prescott: https://orcid.org/0000-0002-5901-7371

Tim Raine: https://orcid.org/0000-0002-5855-9873

Jack Satsangi: https://orcid.org/0000-0002-6357-9684

Shriya Sharma: https://orcid.org/0000-0001-5237-757X

Tara Shrestha: n/a

Michelle Strickland: https://orcid.org/0000-0001-8053-400X

R. Alexander Speight: https://orcid.org/0000-0003-3184-9181

Christopher J. Stewart: https://orcid.org/0000-0002-6033-338X

James M. S. Wason: https://orcid.org/0000-0002-4691-126X

Kevin Whelan: https://orcid.org/0000-0001-5414-2950

Ruth Wood: https://orchid.org/0000-0002-8296-1774

Nicola J. Wyatt: https://orcid.org/0000-0001-5050-327X

Xinyue Zhang: https://orcid.org/0000-0002-5321-4235

For peer review only

Abstract

INTRODUCTION

Characterised by chronic inflammation of the gastrointestinal tract, symptoms of inflammatory bowel disease (IBD), including diarrhoea, abdominal pain and fatigue, can significantly impact patient quality of life. Therapeutic developments in the last 20 years have revolutionised treatment. Despite this, clinical trial and real-world data show primary non-response rates up to 40%. A significant challenge is an inability to predict which class of treatment will benefit individual patients.

Current understanding of IBD pathogenesis implicates complex interactions between host genetics and the gut microbiome. Most cohorts studying the gut microbiota to date have been underpowered, examined single treatments, and produced heterogeneous results. Lack of cross-treatment comparisons and well-powered independent replication cohorts hampers the ability to infer real-world utility of predictive signatures.

IBD-RESPONSE will use multi-omic data to create a predictive tool for treatment response. Future patient benefit may include development of biomarker-based treatment stratification or manipulation of intestinal microbial targets. IBD-RESPONSE and downstream studies have the potential to improve quality of life, reduce patient risk, and reduce expenditure on ineffective treatments.

METHODS AND ANALYSIS

This prospective, multicentre, observational study will identify and validate a predictive model for response to advanced IBD therapies, incorporating gut microbiome, metabolome, single-cell transcriptomics, human genome and clinical data. 1,325 participants commencing

For peer review only - http://bmjopen.bmj.com/site/about/guidelines.xhtml

advanced therapies will be recruited from ~40 UK sites. Data will be collected at baseline, week 14 and week 54. The primary outcome is week 14 clinical response. Secondary outcomes include clinical remission, loss of response in week 14 responders, corticosteroid-free response/remission, time to treatment escalation, and change in patient-reported quality of life and fatigue.

ETHICS AND DISSEMINATION

Ethical approval has been obtained and recruitment is ongoing. Following study completion, results will be submitted for publication in peer-reviewed journals and presented at scientific meetings. Publications will be summarised at www.ibd-response.co.uk.

TRIAL REGISTRATION NUMBER

ISRCTN96296121 (pre-results)

Article Summary

STRENGTHS AND LIMITATIONS OF THIS STUDY

- IBD-RESPONSE is the largest precision medicine study of its kind to date, designed to develop a predictive tool for treatment response – or failure to respond – to IBD treatment, promoting *a priori* selection of the right drug, for the right patient, at the right time.
- IBD-RESPONSE will establish one of the largest biorepositories of longitudinal stool, blood, matched tissue and organoids with detailed linked phenotypic, diet, genetic and treatment outcome data in patients with IBD.
- This study will use a standardised yet pragmatic methodology for sample collection, processing and storage, allowing multicentre participation, improving generalisation and transferability of findings to real-world clinical practice.

For peer review only - http://bmjopen.bmj.com/site/about/guidelines.xhtml

For peer review only

- Remote ‘at home’ patient data and stool collection will allow >40 IBD centres to contribute to recruitment, increasing power in this multi-omics study when compared to other studies in the field that generally recruit from one or a small number of centres.
- Whilst recruiting sites are limited to the UK only, identified cross-validation cohorts from the UK and Canada will increase generalisability of our findings.

For peer review only

For peer review only - http://bmjopen.bmj.com/site/about/guidelines.xhtml

INTRODUCTION

Crohn's disease (CD) and ulcerative colitis (UC) are the principal forms of inflammatory bowel disease (IBD).(1) Characterised by symptoms including diarrhoea, rectal bleeding, abdominal pain and extra-intestinal features such as fatigue, IBD can have a substantial negative impact on patient quality of life.(2) Approximately 20% of CD patients and 10% of UC patients are unable to work due to their condition.(3) The global prevalence of IBD is rising. In the United Kingdom (UK), 1 in 125 people are currently affected, with prevalence expected to reach 1 in 100 by 2028.(4, 5)

The biologics era has revolutionised IBD treatment in the last twenty years. Patients and clinicians have more advanced therapies to choose from than ever before. Several biologic classes are now licensed in the UK, targeting tumour necrosis factor alpha (TNFα) (including infliximab and adalimumab), interleukin (IL)-12/23 (ustekinumab) cytokine pathways, or the gut-homing α4β7 integrin (vedolizumab). In addition, recently licensed small molecule therapies for UC include the Janus kinase inhibitors (JAKi) tofacitinib, filgotinib and upadacitinib, and the sphingosine-1-phosphate receptor (S1PR) modulator ozanimod. With several additional therapies in advanced stages of development or having completed phase 3 randomised clinical trials, the number of treatments available to patients is likely to increase.(6)

Current understanding of biological mechanisms driving the pathogenesis and natural history of IBD, implicates complex interactions between host genetics and the gut microbiome (bacteria, viruses, fungi, archaea and phage).(7) Whilst large clinical cohorts for human genetic discovery have led to major advances in understanding disease pathogenesis,(8) cohorts for the study of gut microbiota have mostly been under-powered. Nonetheless,

For peer review only - http://bmjopen.bmj.com/site/about/guidelines.xhtml

existing research intriguingly suggests utility of microbiome signatures in predicting response to therapy. In a small prospective study of 85 patients starting vedolizumab therapy, greater alpha-diversity and higher abundance of *Roseburia inulinivorans* and a Burkholderiales species at baseline were associated with therapeutic-induced remission in CD.(9) Incorporation of microbial taxonomy data alongside clinical data in a predictive model produced an area under the receiver operating characteristic curve (AUC) of 0.776 (compared to an AUC of 0.619 using clinical data only). A larger study of 232 patients receiving ustekinumab implicated *Bacteroides* and *Faecalibacterium* as predictors of treatment response.(10) Here, a predictive model of response to ustekinumab using clinical metadata produced an AUC of 0.616, rising to 0.844 when combined with baseline bacterial profile data.

In a study of 185 (77 UC, 108 CD) patients commencing anti-cytokine (anti-TNFα or anti-IL12/23) or anti-integrin (vedolizumab) therapy, metabolomic and proteomic analysis of blood in addition to taxonomic and functional profiling of stool samples was conducted.(11) Among patients receiving anti-cytokine therapy, 120 enzymes were differentially abundant in baseline samples of remitters versus non-remitters. Single-species dominance (>50% of enzyme copies in >50% of samples explained by a single species) was observed for 8/120 enzymes. *Eggerthella lenta* was dominant for 5/8 enzymes, 3 of which are involved in secondary bile acid biosynthesis. Metabolomic analysis of baseline blood samples revealed significant enrichment of serum secondary bile acids in patients achieving week 14 clinical remission. Analysis of paired baseline stool samples revealed a significant positive correlation with the abundance of 7α/β-dehydroxylation enzymes (responsible for primary to secondary bile acid conversion), the presence of which was associated with a preferential response to anti-cytokine therapy. This was replicated in a small validation cohort of 46 patients initiating anti-TNFα (infliximab) therapy.

For peer review only - http://bmjopen.bmj.com/site/about/guidelines.xhtml

Beyond IBD, further proof of concept that the gut microbiome is of prognostic importance in the context of systemically administered immune-targeted therapies is found with immune checkpoint inhibitor treatment in cancer, where experimental animal data demonstrated the beneficial impact of microbial modulation on treatment outcome.(12-14)

Whilst these previous studies were important first steps to utilising gut microbial signatures in stratified treatment algorithms, they were mostly underpowered, examined single treatments, used different sequencing technologies, and produced heterogeneous non-overlapping results. The lack of cross-treatment comparisons and well-powered independent replication cohorts hampers the ability to infer real-world utility of these predictive signatures, and to move from observations of association to causation in IBD.(15)

METHODS AND ANALYSIS

Study rationale

A significant challenge to effective, personalised use of biologic or small molecule therapies (collectively termed 'advanced therapies') in IBD is an inability to predict which class of treatment is most likely to benefit an individual patient. Despite increasing therapeutic options, clinical trial and real-world efficacy data show primary non-response rates of up to 40% across all therapeutic classes and in those with initial symptomatic benefit, up to 40% lose response by 1 year.(1, 16-18) Consequently, complications of chronic, active inflammation including strictures, fistulae and malignancy, continue to affect a substantial number of patients and have a negative impact on patient quality of life.(2) Up to 30% of CD patients require surgical intervention within 10 years of diagnosis, and around 15% of UC patients will ultimately require a colectomy.(19) Off-target side effects may also occur, including infection and malignancy.(1) With average treatment costs of £6,156/year for CD

For peer review only

For peer review only - http://bmjopen.bmj.com/site/about/guidelines.xhtml

and £3,084/year for UC, future treatment algorithms must avoid the potential morbidity and additional cost associated with expensive treatments that do not benefit individual patients.(20) The absolute importance of precision medicine research to identify biomarkers for treatment stratification and develop prognostic algorithms was highlighted by two recent national research prioritisation exercises incorporating responses from almost 3,000 patients, their families and friends.(1, 21) Validated prognostic models for treatment stratification do not exist and understanding of mechanisms controlling treatment non-response is limited.

Scientific objectives of IBD-RESPONSE

The primary scientific objective of IBD-RESPONSE is to identify and validate a predictive model for clinical response or failure to respond to advanced therapies in IBD after 14 weeks of therapy (the primary clinical outcome, see below). Modelling will incorporate gut microbiome, human genome, blood and intestinal single cell transcriptome data, and detailed clinical data. Through data derived from the nested CD-metaRESPONSE cohort, predictive modelling will also include detailed dietary information and blood and faecal metabolome data. The co-primary scientific objective of IBD-RESPONSE is to determine the relationship between clinical response and remission at week 14 and baseline gut microbiome.

Secondary scientific objectives of IBD-RESPONSE are to determine if there is a relationship between the microbiome at baseline or changes in the microbiome following advanced therapy with any of the secondary clinical outcomes (*Box 1*). Further exploratory scientific objectives of IBD-RESPONSE are listed in *Box 2.*

- Clinical remission at week 14.
- Clinical response at week 54.
- Clinical remission at week 54.

For peer review only - http://bmjopen.bmj.com/site/about/guidelines.xhtml

- Loss of response at week 54 in week 14 responders.
- Durable corticosteroid-free response or remission at week 54 defined as receiving no corticosteroids between week 14 and week 54 assessments inclusive and not meeting criteria for loss of response.
- Time to treatment escalation from baseline, defined as:
    - Advanced therapy switch due to lack of efficacy/those with loss of response (does not include biosimilar switch or switch from IV to SC route).
    - Dose intensification of drug due to lack of efficacy (does not include intensification based on therapeutic drug monitoring without flare in responders).
    - Resectional intestinal surgery (does not include examination under anaesthesia procedures in patients with perianal CD).
    - Induction or dose escalation of corticosteroids.
- Time to treatment escalation as defined above, but disregarding dose intensification.
- Time to treatment escalation as defined above, among week 14 responders.
- Time to discontinuation of index drug (persistence).
- Incidence of and time to potential side effects of treatment during follow up.
- Continuation of drug at week 14 and/or week 54 in those not meeting criteria for response and/or remission.
- Change from baseline SF subscore at week 14 and/or 54 (both CD and UC).
- Change from baseline RB subscore at week 14 and/or 54 (UC only).
- Change from baseline AP subscore at week 14 and/or 54 (CD only).
- Development of anti-drug antibodies by week 14 or 54.

For peer review only - http://bmjopen.bmj.com/site/about/guidelines.xhtml

- Change in CRP from baseline at week 14 or 54 (50% reduction or absolute value ≤5mg/L deemed as clinically significant).
- Change in faecal calprotectin from baseline at week 14 or 54 (50% reduction or absolute value ≤100µg/g deemed as clinically significant).
- Endoscopic remission during follow up (Mayo endoscopic subscore ≤1 for UC or SES-CD ≤2 for CD).
- Change in quality of life, physical activity dietary intake, joint pain and fatigue as measured by study questionnaires.

Box 1. IBD-RESPONSE secondary clinical outcome measures

Box 1 abbreviations: abdominal pain (AP), Crohn's disease (CD), c-reactive protein (CRP), intravenous (IV), rectal bleeding (RB), subcutaneous (SC), stool frequency (SF), simple endoscopic score for Crohn's disease (SES-CD), ulcerative colitis (UC)

- Test the association of microbial metabolites (metabolome) in stool or plasma, human genetics and/or single cell transcriptome data from blood or intestinal tissue with the above primary and secondary objectives.
- Determine the influence of diet on the gut microbiome and treatment response in IBD, and the factors associated with dietary intake in IBD.
- Explore host human genetic-gut microbiome-metabolome interactions in IBD pathogenesis and causal pathways to treatment response.
- Ascertain the utility of archived endoscopy collected FFPE biopsies at predicting/imputing the gut microbiome and for inclusion in the predictive model.

For peer review only - http://bmjopen.bmj.com/site/about/guidelines.xhtml

- Establish a longitudinal tissue, organoid and stool biobank from this well characterised clinical cohort.

Box 2. IBD-RESPONSE exploratory scientific objectives

Box 2 abbreviations: inflammatory bowel disease (IBD), formalin fixed paraffin embedded (FFPE)

Study design

The design of IBD-RESPONSE and the nested CD-metaRESPONSE studies are summarised in *Figure 1*. This prospective, observational, multi-centre, cohort study will recruit participants with IBD (CD, UC, IBD-unclassified (IBD-U)) who are due to commence either biologic, JAKi or S1PR modulator therapy for symptomatic, clinically active (moderate to severe) luminal disease. Participants do not have to be naïve to advanced therapies and may be recruited when switching within or between class of advance therapy. Participants may be taking or planned to start concurrent thiopurines or methotrexate as combination therapy. Participation in the study will not change standard clinical care received. Detailed longitudinal clinical data will be collected alongside stool, blood and (where possible) biopsy samples, patient reported outcome measures, and dietary intake. Planned recruitment of 1,325 participants will consist of 762 patients with CD and 563 patients with UC (or IBD-U).

[Figure 1]

Data will be collected at baseline (prior to starting treatment), week 14 (following completion of induction therapy) and week 54. Participants will collect stool samples at each study timepoint. If a participant attends hospital within the baseline and/or week 14 study window, two blood samples (per timepoint) will be collected. Where a participant undergoes

For peer review only - http://bmjopen.bmj.com/site/about/guidelines.xhtml

endoscopy as part of routine clinical care during the study period, up to 12 research biopsies will be collected.

Of the 762 CD participants, 300 will be consented to take part in a sub-cohort (CD-metaRESPONSE). Clinical data collection will occur at the same time points as in the main cohort. Additional study components will include completion of a 4-day food diary questionnaire, capturing all food and drink consumed at the baseline and week 14 timepoints. This is in addition to the food frequency questionnaire completed by all participants. CD-metaRESPONSE participants will also be required to provide two additional stool tubes (one at baseline and one at week 14) for faecal metabolome analysis.

All participants recruited to IBD-RESPONSE will be invited to co-recruit to IBD BioResource (if not already participating). IBD BioResource is a national platform and recallable biorepository linked to the National Institute for Health and Care Research (NIHR) BioResource that is designed to expedite IBD research, currently with >36,000 participants.(22). Co-recruiting participants to IBD-RESPONSE and the IBD BioResource platform, will generate a rich dataset and provide a long term means of archiving data from IBD-RESPONSE to facilitate ongoing research and maximise downstream patient benefit. To minimise participant burden, the contact for recruitment to IBD BioResource can occur any time in the 12 months following consent to IBD-RESPONSE. If a participant ultimately decides not to participate in the IBD BioResource they will not be withdrawn from IBD-RESPONSE.

If a participant discontinues treatment prior to week 14 or week 54 follow up, the next timepoint assessment will be brought forward and completed as a treatment discontinuation assessment. Where a participant does not respond to the first prescribed advanced therapy and a second (or third) advanced therapy is subsequently prescribed, this discontinuation

For peer review only

assessment will act as the baseline sample and data collection assessment for the successive advanced therapy. Follow up samples and data collection will occur at week 14 and week 54 following commencement of each successive agent. The participant may remain in the study up to 54 weeks after commencement of a particular agent or until the end of the study period. We anticipate that up to 40% of patients will not respond to the initial prescribed therapy or will lose response by 1 year and will move on to a second (or third) advanced therapy. We therefore anticipate that recruitment of 1,325 participants may capture approximately 1,760 new treatment initiation episodes.

Study setting

This multi-centre cohort study will be conducted at >40 study centres, based within National Health Service (NHS) Trusts across the UK. All sites must be able to accommodate the needs of the IBD-RESPONSE cohort including clinical engagement, research nurse support and facilities for assessments.

Eligibility criteria

Individuals will be deemed eligible to enrol in the study if they fulfil all inclusion criteria and meet none of the exclusion criteria (*Box 3*).

For peer review only

INCLUSION CRITERIA

Individuals must fulfil all the following criteria to be enrolled in the study.

- Aged 16 years and over.
- Diagnosis of IBD: CD, UC or IBD-U.*
- Already participating or willing to be approached for participation in IBD BioResource.
- Willing and able to provide informed consent.
- Willing to undertake study procedures including:
  - Completion of study questionnaires
  - Collection of home stool specimens
- Provision of blood and (where applicable) biopsy specimens.
- Symptoms of active luminal IBD (see definition of 'Clinically active disease', *Table 1*).
- At least one biochemical, endoscopic or radiological marker of active disease within 12 weeks of study consent (see *Box 4* and *Box 5*)
- Intention of clinical team to commence one of the following advanced therapies for active luminal IBD within 6 weeks of consent:△
  - Infliximab
  - Adalimumab
  - Vedolizumab
  - Ustekinumab
  - Tofacitinib
  - Filgotinib
  - Upadacitinib
  - Ozanimod

*Individuals with IBD-U will be managed as per the UC relevant protocol.

△Participants may be advanced therapy-naïve or -exposed. Any new biologic or small molecule drug that becomes licensed for the treatment of IBD during the planned study period will be permitted to allow study inclusion. Drugs used through Early Access to Medicines Schemes, compassionate use or expanded access schemes for unlicensed therapies are also permitted. Patients starting immunosuppressant monotherapy with a thiopurine or methotrexate are not eligible to take part. However, use of these treatments as part of combination therapy with an advanced therapy is not an exclusion to enrolment.

For peer review only

EXCLUSION CRITERIA

Individuals meeting any of the following criteria will not be eligible to participate in the study.

- Receiving oral corticosteroids for any indication where the dose is unlikely to be weaned by week 14.^
- Planned bowel resection surgery within 14 weeks of commencing therapy.
- Advanced therapy being commenced as rescue for ASUC.
- Advanced therapy being commenced as part of a CTIMP.
- Presence of an ileal pouch anal anastomosis.
- Presence of a stoma.
- Perianal CD in the absence of active luminal inflammation.
- Antibiotics or short-term (≤4 weeks) use of probiotics within the preceding 2 weeks.†
- FMT within the preceding 12 weeks or planned FMT within 14 weeks of commencing advanced therapy for IBD.‡

^Examples may include long term steroids for IBD where weaning by 14 weeks may not be possible irrespective of response to advanced therapy, or concurrent diagnosis where long term steroids are used e.g. polymyalgia rheumatica.

†Use of long-term (>4 weeks), stable doses of probiotics does not exclude individual participation but should be noted in the eCRF.

‡Use of antibiotics or prior FMT outside the exclusion period is permitted. Antibiotic use in the preceding 1 year and ever having received FMT will be noted in the eCRF.

Box 3. Eligibility criteria for IBD-RESPONSE

Box 3 abbreviations: Acute severe ulcerative colitis (ASUC), Clinical Trial of an Investigational Medicinal Product (CTIMP), Crohn's disease (CD), electronic case report form (eCRF), faecal microbial transplantation (FMT), inflammatory bowel disease (IBD), inflammatory bowel disease-unclassified (IBD-U), ulcerative colitis (UC).

Participants must have at least one biochemical, endoscopic or radiological (CD only) marker of clinically active luminal disease within 12 weeks of study consent (see *Box 4* (CD) and *Box 5* (UC)). Endoscopic assessment of disease activity will be judged locally and may

For peer review only - http://bmjopen.bmj.com/site/about/guidelines.xhtml

be assessed prospectively or retrospectively. Whilst pregnancy may influence advance therapy selection, pregnancy is not an exclusion criterion.

CD patients must also have at least one of the following documented within 12 weeks prior to consent:

- Faecal calprotectin ≥250 μg/g.
- CRP ≥6 mg/L.
- Any endoscopic evidence of active CD, defined as ulceration (with at least one ulcer ≥5mm) judged locally from available clinical data (as an approximation equivalent to SES-CD of ≥4 for ileal disease or ≥6 for ileocolonic or colonic disease.
- Active inflammatory disease on imaging (MRI/CT/ultrasound) judged locally from available clinical data.

Box 4. IBD-RESPONSE additional inclusion criteria: CD

Box 4 abbreviations: Crohn's disease (CD), C-reactive protein (CRP), Computed tomography (CT), Magnetic resonance imaging (MRI), Simple Endoscopic Score for Crohn's Disease (SES-CD)

UC/IBD-U patients must also have at least one of the following documented within 12 weeks prior to consent:

- Faecal calprotectin ≥250 μg/g.
- CRP ≥6 mg/L.
- Any endoscopic evidence of at least moderately active ulcerative colitis (of any extent including proctitis), defined as features of MCS endoscopic sub-score ≥2 (marked erythema, lack of vascular pattern, friability, erosions, spontaneous bleeding or ulceration.

Box 5. IBD-RESPONSE additional inclusion criteria: UC and IBD-U

For peer review only

Box 5 abbreviations: C-reactive protein (CRP), inflammatory bowel disease-unclassified (IBD-U), Mayo Clinic Score (MCS), ulcerative colitis (UC)

Clinical outcome measures

Key definitions related to clinical outcome measures can be found in *Table 1*.

Clinically active disease	• CD: Unweighted PRO-2 (CD) of average daily SF subscore ≥4 and/or average daily AP subscore ≥2.<br>• UC: Total PRO-2 (UC) ≥3 with RB subscore ≥1.<br>PRO-2 data will be entered by patients over 4 days (minimum 2 days PRO-2 data is permissible for PRO-2 calculation).
Clinical remission	Patient remains on drug and meets the following criteria:<br>• CD: Unweighted PRO-2 (CD) average daily SF subscore ≤2.8 and average daily AP subscore ≤1 (and neither worse than inclusion scores at baseline).<br>• UC: PRO-2 (UC) SF subscore ≤1 with a decrease of ≥1 point(s) in SF subscore from baseline, plus RB subscore = 0.<br>**AND** an absence of any of the following at time of assessment:<br>• Resectional bowel surgery at any time after baseline until time of current assessment.<br>• Current use of oral corticosteroids/failure to wean oral corticosteroids prescribed at baseline.
Clinical response	Meeting criteria for clinical remission **OR**:<br>• CD: unweighted PRO-2 (CD) ≥30% reduction in average daily SF subscore and/or ≥30% decrease in average daily AP subscore (and neither worse than inclusion scores at baseline).<br>• UC: total PRO-2 (UC) decrease ≥3 from baseline.

For peer review only - http://bmjopen.bmj.com/site/about/guidelines.xhtml

	**AND** an absence of any of the following at time of assessment:<br>• Resectional bowel surgery at any time after baseline until time of current assessment.<br>• Current use of oral corticosteroids/failure to wean oral corticosteroids prescribed at baseline.
Week 14 non-response	• Not meeting clinical response criteria AND not having stopped drug for any reason other than lack of efficacy.
Week 54 loss of response	• Not meeting clinical response criteria at week 54 having met clinical response criteria at week 14 (AND not having stopped drug for any reason other than inefficacy between week 14 and 54 assessments).

Table 1. IBD-RESPONSE key clinical definitions

Table 1 abbreviations: abdominal pain (AP), Crohn's disease (CD), patient reported outcome (PRO)-2, rectal bleeding (RB), stool frequency (SF), ulcerative colitis (UC).

Primary clinical outcome measures

Clinical response at week 14 after commencing therapy.

Secondary clinical outcome measures

Secondary clinical outcome measures are listed in *Box 1*.

Sample size calculations

The sample size (n=1,325 cases overall, including n=300 cases for CD-metaRESPONSE sub-cohort) was chosen to ensure sufficient power to answer the co-primary scientific objective (to detect associations between microbiome measures and clinical response or remission) and key exploratory scientific objectives (to detect associations between metabolites and clinical response or remission, and to detect associations between

For peer review only - http://bmjopen.bmj.com/site/about/guidelines.xhtml

genetics and microbiome measures). Sample size calculations for predictive models require extensive assumptions about the number and effect size of associations and the correlation structure of the data. We noted predictive models built with microbiome and/or metabolite measures with high in-sample predictive accuracy (including AUC=0.78 with n=84 from Ananthakrishnan et al.(9), AUC=0.91 with n=76 from Ding et al.(23), and AUC=0.84 with n=232 from the CERTIFI study(10)), implying that n=300 individuals (from CD-metaRESPONSE) and n=1,325 individuals (from IBD-RESPONSE), should be sufficient for high predictive in-sample accuracy.

Sample size for analysis of primary objective

We took plausible effect sizes for the primary scientific objective analysis from the effect of *Bacteroides* levels in stool on ustekinumab response (d=0.66, from the from the CERTIFI study(10)) and the effect of anti-drug immunogenicity on remission after anti-TNFα treatment (d=0.30, from the PANTS study(16)). We calculated power for a simple two-sample t-test (using the R package pwr), assuming the expected non-response rate at 14 weeks (23.8%) and non-remission rate at 54 weeks (63.1%) from the PANTS study. We assumed a conservative Bonferroni-corrected significance threshold of 1e-5 (correcting for 5000 microbiome measures). The sample size of n=1,325 gives a power of close to 100% for the larger *Bacteroides* effect size for both week 14 response and week 54 remission, and 57% and 80% for the smaller anti-drug immunogenicity effect size for week 14 response and week 54 remission, respectively.

Sample size for analysis of secondary and exploratory scientific objectives

We took plausible effect sizes for the exploratory metabolite analysis from the effects of three selected stool lipid and bile acid metabolites on anti-TNFα response from Ding et

For peer review only - http://bmjopen.bmj.com/site/about/guidelines.xhtml

al.(23): faecal triglyceride (d=1.00), and two bile acid metabolites; BA1 (d=0.89) and BA3 (d=0.70). We assumed a conservative Bonferroni-corrected significance threshold of 5e-5 (correcting for 1000 metabolites). For n=300, this gives a power of 99.7%, 91% and 74% for faecal triglyceride, BA1 and BA3 respectively for week 14 response, and 100%, 99.8% and 91% for week 54 remission.

We do not anticipate that this study on its own will be well powered to detect new associations between genotype and microbiome measures in IBD. It is known from studies of healthy individuals that genetic variants that explain more than 3% of variation (R^2=0.03) in microbial abundance are rare.(24) Assuming a conservative significance threshold of < 1e-11 (correcting for 1e6 independent genotypes and 5000 microbiome measures), n=1,325 samples would only have 33% power to detect associations with R^2=0.03 (calculated using genpwr(25)). We will therefore combine our samples with a further genotype/microbiome study of IBD (PREdiCCt) to increase sample size to n=2,325. This will provide us 80% power to detect genetic associations with R^2>0.025. In the case where such genotype/microbiome measure associations exist, and are associated with a causal biomarker for week 54 remission, a Mendelian Randomization analysis would have >80% power to demonstrate causality of this biomarker when the causal effect odds ratios is larger than 2 (calculated using mRnd(26)).

Study procedures and measures

Participants ≥16 years old may be identified from a variety of settings such as outpatient clinics (face-to-face or virtual), flare assessments, IBD clinical nurse specialist helplines/email contact, endoscopy examinations, infusion suites, multidisciplinary team meetings and virtual biologics clinics (*Figure 1*). Consent will be taken electronically using a

For peer review only - http://bmjopen.bmj.com/site/about/guidelines.xhtml

REDCap (Research Electronic Data Capture) online database. Full informed e-consent will be supported by an appropriately delegated member of the study team, using a laptop, tablet or mobile device in the patient's own home, or using a hospital tablet, laptop, computer or patient mobile device during a scheduled visit to hospital as part of routine clinical care. Paper copies of the consent form will be made available for those patients unable to access e-consent. If a later decision is made not to commence an advanced therapy for IBD, the participant will not be eligible to continue in the study and must be withdrawn. Data collected up to the point of withdrawal may be used for the study. Any samples collected will be used for research within IBD-RESPONSE or stored for future research.

All research activity will be completed by the participant either remotely or during hospital visits scheduled to deliver routine clinical care (see *Figure 2*). Participants will be asked to complete data collection after consent and before starting treatment (baseline), and at week 14 and week 54 following commencement of advanced therapy in line with routine dosing schedule visits. This will include patient questionnaires, stool samples and, where applicable, blood and biopsy specimens. Participants will be asked to complete questionnaires with data entered directly into the study specific REDCap database. Paper questionnaires will be made available for those participants without access or who express a preference to complete in paper format. Participants will be asked to complete questionnaires related to disease activity, health, physical activity, quality of life and diet at the three assessment timepoints (see *Box 6*).

Participant samples

All sample collection and processing will be standardised, with full requirements detailed in a study Sample Collection Manual.

For peer review only - http://bmjopen.bmj.com/site/about/guidelines.xhtml

Stool samples will be collected by participants at home using stool collection kits and returned using a prepaid Royal Mail Safebox™. All participants will collect two stool samples per study assessment timepoint (DNA Genotek OMNIgene®•GUT tube and universal polystyrene tube). Participants recruited to the CD-metaRESPONSE sub-cohort will be required to collect a third stool sample at baseline and week 14 (DNA Genotek OMNImet®•GUT tube). DNA extraction and metagenomic shotgun sequencing will be performed on buffered samples. Calprotectin will be measured in unbuffered stool. Remaining fresh stool and nucleic acids will be cryopreserved for use in future research.

Participants who attend a clinical appointment prior to commencing advanced therapy or within the week 14 visit window (week 10-20; week 12-16 preferred) will be asked to provide two blood samples (lithium heparin (LiH) tube and ethylenediaminetetra-acetic acid (EDTA) tube). Blood samples will be utilised for single cell analysis, plasma extraction and cryopreservation. Any remaining blood samples or derivatives will be stored at Newcastle Biobank for use in future research. Participants will not be asked to attend hospital specifically for blood sample collection. If participants are not scheduled to attend hospital face-to-face within either study assessment window, blood samples will not be taken.

If a participant has a lower gastrointestinal endoscopy as part of planned care during study participation, up to 12 research biopsies (one set of six biopsies to be collected from the colon in all participants and a further set of six biopsies from the ileum in CD participants) will be taken. Where a participant meets the study eligibility criteria and a disease assessment endoscopy is planned prior to starting therapy, consent should be received ahead of their planned endoscopy to enable the collection of research biopsies. Biopsy samples will be utilised for single cell sequencing and organoid generation. 16S rRNA gene sequencing of

For peer review only - http://bmjopen.bmj.com/site/about/guidelines.xhtml

both FFPE and fresh tissue will also be undertaken. Any remaining biopsy samples will be stored for use in future research.

[Figure 2]

COMPLETED BY ALL PARTICIPANTS AT BASELINE, WEEK 14 AND WEEK 54
**PRO-2 (CD or UC version depending on diagnosis)**<br>PRO-2 is a validated questionnaire measuring patient reported outcomes including stool frequency, abdominal pain and rectal bleeding.(27, 28).<br>**Bristol Stool Form Scale (BSFS)**<br>The BSFS is a 7-point scale that helps describe stool shape and consistency and assess bowel patterns and habits.(29)<br>**IBD-Control**<br>The IBD-Control questionnaire comprises 13 items plus a visual analogue scale (VAS) ranging from 0–100.(30) The questionnaire measures patient related outcome of their disease state during the past 2 weeks.<br>**PROMIS-Fatigue 8a Short Form**<br>The Patient Reported Outcomes Measurement Information System (PROMIS), are validated questionnaires that help evaluate patients' quality of life.(31)<br>**EQ-5D-5L**<br>The EQ-5D-5L is a quality-of-life questionnaire and is a widely used generic PROM incorporating five domains: 1) mobility, 2) self-care, 3) usual activities, 4) pain/discomfort, 5) anxiety/depression.(32, 33) Scores for each domain are combined to describe the patient's state of health.<br>**International Physical Activity Questionnaire (IPAQ)**

For peer review only - http://bmjopen.bmj.com/site/about/guidelines.xhtml

IPAQ is a commonly used self-reported questionnaire to estimate physical activity and sedentary behaviours for adults across a range of socio-economic settings.(34) The IPAQ measures the type of physical activities people do as part of their everyday lives.(34)

IBD-RESPONSE Joint Pain and Stiffness Score (JPSS)

The Ankylosing Spondylitis Disease Activity Score (ASDAS) is a patient reported questionnaire which quantifies clinical disease activity in Ankylosing Spondylitis (AS) and combines five disease activity variables (four 10-point Likert scale patient symptom responses and a CRP measurement), to produce a single score.(35-37) We believe joint pain to be an under-recognised symptom in active inflammatory bowel disease which may change in response to therapy as inflammation resolves/fails to resolve and so we have modified the ASDAS to assess joint pain, swelling and stiffness in all IBD-RESPONSE patients irrespective of whether they have a rheumatological diagnosis. We have called this modified score the IBD-RESPONSE Joint Pain and Stiffness Score (JPSS).

Food frequency questionnaire (FFQ)

The FFQ will be completed by participants directly into the study specific REDCap database and can also be completed on paper. The FFQ requests information on 175 food items, their typical portion size and frequency of consumption and has been extensively validated for measuring nutrient intakes in adults.(38) Data are converted to nutrient intake using the Composition of Foods Integrated Dataset (COFIDs), as well as diet quality indices and other food components (e.g. polyphenols) and food categorisation (e.g. ultra-processed foods). The FFQ data will initially be analysed at the Centre for Healthcare Randomised Trials (CHaRT), University of Aberdeen and other collaborating institutions at the Department of Nutritional Sciences, King's College London.

COMPLETED BY CD-METARESPONSE PARTICIPANTS AT BASELINE AND WEEK 14 ONLY

4-day food diaries

The 4-day food diary measures current food intake. It will comprehensively and prospectively measure all intake allowing calculation of energy and nutrient intake, dietary

For peer review only

For peer review only - http://bmjopen.bmj.com/site/about/guidelines.xhtml

indices (e.g. diet diversity and Mediterranean diet), intake of ultra-processed foods, prebiotic and emulsifier intake.
Box 6. Summary of patient questionnaires completed throughout study period

Statistical analysis

The analysis approach for our primary objective will be to test for the association between features of the patient microbiome at baseline and primary clinical response to treatment at 14 weeks. Features will include alpha diversity, abundance of bacterial taxa (including species, genus and phyla) as well as the abundance of genes within various microbial pathways (for example using KEGG pathways, MetaCyc metabolic pathways and gene families). Association testing will be carried out using negative binomial regression, controlling for total sequence depth and pre-defined technical and clinical confounders, and significance will be determined using Benjamini-Hochberg multiple testing correction to ensure a false discovery rate of less than 5%. Individuals who are lost to follow-up or withdraw from the study before 14 weeks will be removed from the analysis, though we will also carry out a robustness analysis where we include individuals lost to follow-up as non-responders to ensure the results are robust to this choice.

For the predictive modelling aim, we will use a random forest classifier to predict primary response to treatment at 14 weeks using microbiome, host genetic and clinical features at baseline. Model parameters will be tuned and accuracy assessed using nested cross-validation. This full model will be compared to a clinical-variables-only model, with improvement measured by area under the received operator curve, as well as positive and

For peer review only - http://bmjopen.bmj.com/site/about/guidelines.xhtml

negative predictive power. A further model will be fitted using the same approach including metabolomic and dietary data on the CD-metaRESPONSE subset.

Secondary and exploratory objective analyses will use the same general analysis approach as described above. For time-dependent events, such as treatment escalation due to loss of response, a Cox proportional hazards regression will be used to assess the impact of microbiome features on time to event, with patients censured at 54 weeks, last recorded (if lost to follow-up) or date of withdrawal (if withdrawn). The dietary data will be analysed to test the association between primary response and measures of specific nutrients (such as dietary fibre) and dietary indices will be used to assess adherence to certain recommended diets (such as achievement of food-based dietary guidelines or a "Mediterranean diet"). Host genotype data will be used to test for associations between primary response and generate polygenic risk scores of susceptibility to Crohn's disease and ulcerative colitis, as well as pre-specified variants associated with response to therapy (including HLA-DQA1*05). In all these individual analyses, Benjamini-Hochberg will be applied used to control the false discovery rate at 5%.

Statistical analysis will be carried out in R.

Replication

Scaling up microbiome discoveries and providing validation of results is needed to benefit patients. Whilst IBD-RESPONSE is the largest project of its kind to date, it is a UK-only programme. Translating findings to maximise patient benefit requires international collaboration. Standardised data collection and data integration from different countries and ethnic groups is key to establishing predictors of treatment response that are generalisable and replicable.

For peer review only

We will validate our predictive model using an appropriate, already assembled microbiome validation cohort. To generate this replication cohort, we will use banked stool DNA from the Prognostic effect of Environmental factors in Crohn's and Colitis Study (PREdiCCt; www.predicct.co.uk), led by Professor Charlie Lees. PREdiCCt is a prospective observational study of IBD participants in clinical remission, designed to identify whether baseline factors (including genetics, dietary habits and gut microbiota) predict subsequent disease flare. We will perform metagenomic sequencing of 1,000 stool samples from patients who experienced a disease flare during the PREdiCCt study. We expect approximately 40% of PREdiCCt patients to experience a disease flare requiring commencement of biologics. As these patients all have baseline (clinical remission) stool microbiome samples in storage, they provide a well matched and cost-efficient set of samples for replicating IBD-RESPONSE results.

We will also carry out a collaborative cross-replication analysis of results from IBD-RESPONSE and the Montreal, Canada iGenoMed-MTT cohort, led by Professor John Rioux to evaluate how well microbiome associations and predictive algorithms replicate across continents. This will involve analysis of stored stool samples from 105 Canadian IBD patients collected prior to commencing advanced therapies (following the sequencing and analysis protocols established in IBD-RESPONSE) with treatment outcome data. It will provide a rapid "first-look" analysis to discover and assess microbiome predictors of biological response in an international cohort with extensive metadata, ahead of larger studies coming online over a longer timeframe within IBD-RESPONSE and allow later cross-replication/meta-analysis.

For peer review only - http://bmjopen.bmj.com/site/about/guidelines.xhtml

Potential future benefit to patients

IBD-RESPONSE will provide timely and important information regarding associations between the gut microbiome and responsiveness to treatment in IBD. It will likely highlight potential mechanisms through which the microbiota may drive inflammation. We hope that findings from IBD-RESPONSE will lead to new personalised avenues for IBD treatment through discovery and validation of predictive tools that may be incorporated directly into clinical practice or further tested in stratified clinical trials. This could lead to the development of experimental techniques to modify gut microbes; for example, donor selection for faecal microbial transplantation, identification of single or multiple strains of microbes, or use of antimicrobials, phage or microbial metabolites that may be used to induce a more 'treatment responsive' microbiome.

Patient and public involvement statement

IBD-RESPONSE was informed by two national patient research prioritisation exercises in IBD care led by members of our team and involving feedback from 3,000 people living with IBD, their family and friends.(1, 21). These identify aspects of precision medicine, microbiome and diet in IBD as of high importance. The initial IBD-RESPONSE grant proposal to the Medical Research Council (MRC) was reviewed by the NIHR Research Design Service North East and North Cumbria Patient and Public Involvement Panel and was presented and discussed at the 2020 Crohn's & Colitis UK Patient and Public Involvement in Research Day. IBD-RESPONSE has been supported by two patient representatives in the Study Oversight Committee (SOC) since inception. The study team have engaged with lay members of the SOC to ensure all patient facing documents including the participant information sheet (PIS), consent forms, stool collection guidance for patients and study questionnaires have

For peer review only - http://bmjopen.bmj.com/site/about/guidelines.xhtml

undergone review. Patients will also be involved in dissemination activities relating to outputs from this research.

ETHICS AND DISSEMINATION

Ethical approval for the study was obtained from the Wales Research Ethics Committee 5 (reference 21/WA/0228). Recruitment to IBD-RESPONSE began in February 2022 and is currently ongoing at sites around the UK.

In line with the Newcastle University and The Newcastle upon Tyne Hospitals NHS Foundation Trust research data policy, datasets will be kept for at least five years after the date they were last accessed. Metadata linked to genomic and metagenomic datasets will include anonymised clinical information. Examples include diagnosis (UC/CD/IBD-U), disease location, disease behaviour, complications, extra-intestinal manifestations, co-morbidities, family history, smoking history, surgical interventions, and outcomes from prior drug therapies.

Raw data files in the original format (e.g. fastq) and the accompanying anonymised phenotypic data will be uploaded to a public repository e.g. the NCBI database of Genotypes and Phenotypes (dbGaP) at https://www.ncbi.nlm.nih.gov/gap/.

As part of CD-metaRESPONSE, microbial sequence and faecal/serum metabolomic data will be integrated with single-cell RNA sequencing, human genomics and clinical outcome data; the whole dataset will be made available to other investigators and will be archived long term within the IBD BioResource to facilitate downstream research.

The CI, SMG, Sponsor, Funders and research team members are committed to ensure that the research findings are shared. Findings will be written up and submitted to a peer-reviewed scientific journal. Findings will be presented by the study team at national and international conferences for example the British Society of Gastroenterology (BSG) annual

For peer review only

For peer review only - http://bmjopen.bmj.com/site/about/guidelines.xhtml

meeting, the European Crohn's and Colitis (ECCO) meeting and Digestive Diseases Week (DDW). The study team will prepare a lay summary of the study findings for dissemination to the study participants and members of the national patient group, Crohn's & Colitis UK. Following study completion, results will be submitted for publication in peer-reviewed journals and presented at national and international scientific meetings.

REGISTRATION DETAILS

Trial registration number: ISRCTN96296121 (pre-results).

For peer review only

For peer review only - http://bmjopen.bmj.com/site/about/guidelines.xhtml

REFERENCES

1. Lamb CA, Kennedy NA, Raine T, Hendy P, Smith PJ, Limdi J, et al. British Society of Gastroenterology consensus guidelines on the management of inflammatory bowel disease in adults. Gut2019.

2. Floyd DN, Langham S, Severac HC, Levesque BG. The economic and quality-of-life burden of Crohn's disease in Europe and the United States, 2000 to 2013: a systematic review. Dig Dis Sci. 2015;60(2):299-312.

3. van der Valk ME, Mangen MJ, Leenders M, Dijkstra G, van Bodegraven AA, Fidder HH, et al. Risk factors of work disability in patients with inflammatory bowel disease--a Dutch nationwide web-based survey: work disability in inflammatory bowel disease. J Crohns Colitis. 2014;8(7):590-7.

4. Jones GR, Lyons M, Plevris N, Jenkinson PW, Bisset C, Burgess C, et al. IBD prevalence in Lothian, Scotland, derived by capture-recapture methodology. Gut. 2019;68(11):1953-60.

5. Hamilton B, Green H, Heerasing N, Hendy P, Moore L, Chanchlani N, et al. Incidence and prevalence of inflammatory bowel disease in Devon, UK. Frontline Gastroenterology. 2021;12(6):461.

6. Wyatt NJ, Speight RA, Stewart CJ, Kirby JA, Lamb CA. Targeting Leukocyte Trafficking in Inflammatory Bowel Disease. BioDrugs. 2021;35(5):473-503.

7. Jostins L, Ripke S, Weersma RK, Duerr RH, McGovern DP, Hui KY, et al. Host-microbe interactions have shaped the genetic architecture of inflammatory bowel disease. Nature. 2012;491(7422):119-24.

8. Luo Y, de Lange KM, Jostins L, Moutsianas L, Randall J, Kennedy NA, et al. Exploring the genetic architecture of inflammatory bowel disease by whole-genome sequencing identifies association at ADCY7. Nat Genet. 2017;49(2):186-92.

9. Ananthakrishnan AN, Luo C, Yajnik V, Khalili H, Garber JJ, Stevens BW, et al. Gut Microbiome Function Predicts Response to Anti-integrin Biologic Therapy in Inflammatory Bowel Diseases. Cell Host Microbe. 2017;21(5):603-10 e3.

10. Doherty MK, Ding T, Koumpouras C, Telesco SE, Monast C, Das A, et al. Fecal Microbiota Signatures Are Associated with Response to Ustekinumab Therapy among Crohn's Disease Patients. MBio. 2018;9(2).

11. Lee JWJ, Plichta D, Hogstrom L, Borren NZ, Lau H, Gregory SM, et al. Multi-omics reveal microbial determinants impacting responses to biologic therapies in inflammatory bowel disease. Cell Host & Microbe. 2021;29(8):1294-304.e4.

12. Routy B, Le Chatelier E, Derosa L, Duong CPM, Alou MT, Daillere R, et al. Gut microbiome influences efficacy of PD-1-based immunotherapy against epithelial tumors. Science. 2018;359(6371):91-7.

13. Matson V, Fessler J, Bao R, Chongsuwat T, Zha Y, Alegre ML, et al. The commensal microbiome is associated with anti-PD-1 efficacy in metastatic melanoma patients. Science. 2018;359(6371):104-8.

14. Gopalakrishnan V, Spencer CN, Nezi L, Reuben A, Andrews MC, Karpinets TV, et al. Gut microbiome modulates response to anti-PD-1 immunotherapy in melanoma patients. Science. 2018;359(6371):97-103.

15. Plichta DR, Graham DB, Subramanian S, Xavier RJ. Therapeutic Opportunities in Inflammatory Bowel Disease: Mechanistic Dissection of Host-Microbiome Relationships. Cell. 2019;178(5):1041-56.

For peer review only - http://bmjopen.bmj.com/site/about/guidelines.xhtml

16. Kennedy NA, Heap GA, Green HD, Hamilton B, Bewshea C, Walker GJ, et al. Predictors of anti-TNF treatment failure in anti-TNF-naive patients with active luminal Crohn's disease: a prospective, multicentre, cohort study. Lancet Gastroenterol Hepatol. 2019;4(5):341-53.

17. Narula N, Peerani F, Meserve J, Kochhar G, Chaudrey K, Hartke J, et al. Vedolizumab for Ulcerative Colitis: Treatment Outcomes from the VICTORY Consortium. Am J Gastroenterol. 2018;113(9):1345.

18. Wils P, Bouhnik Y, Michetti P, Flourie B, Brixi H, Bourrier A, et al. Subcutaneous Ustekinumab Provides Clinical Benefit for Two-Thirds of Patients With Crohn's Disease Refractory to Anti-Tumor Necrosis Factor Agents. Clin Gastroenterol Hepatol. 2016;14(2):242-50 e1-2.

19. Tsai L, Ma C, Dulai PS, Prokop LJ, Eisenstein S, Ramamoorthy SL, et al. Contemporary Risk of Surgery in Patients With Ulcerative Colitis and Crohn’s Disease: A Meta-Analysis of Population-Based Cohorts. Clinical Gastroenterology and Hepatology. 2021;19(10):2031-45.e11.

20. Ghosh N, Premchand P. A UK cost of care model for inflammatory bowel disease. Frontline Gastroenterol. 2015;6(3):169-74.

21. Hart AL, Lomer M, Verjee A, Kemp K, Faiz O, Daly A, et al. What Are the Top 10 Research Questions in the Treatment of Inflammatory Bowel Disease? A Priority Setting Partnership with the James Lind Alliance. J Crohns Colitis. 2017;11(2):204-11.

22. Parkes M, Investigators IBDB. IBD BioResource: an open-access platform of 25 000 patients to accelerate research in Crohn's and Colitis. Gut. 2019;68(9):1537-40.

23. Ding NS, McDonald JAK, Perdones-Montero A, Rees DN, Adegbola SO, Misra R, et al. Metabonomics and the Gut Microbiome Associated With Primary Response to Anti-TNF Therapy in Crohn's Disease. J Crohns Colitis. 2020;14(8):1090-102.

24. Bonder MJ, Kurilshikov A, Tigchelaar EF, Mujagic Z, Imhann F, Vila AV, et al. The effect of host genetics on the gut microbiome. Nat Genet. 2016;48(11):1407-12.

25. Moore CM, Jacobson SA, Fingerlin TE. Power and Sample Size Calculations for Genetic Association Studies in the Presence of Genetic Model Misspecification. Hum Hered. 2019;84(6):256-71.

26. Brion MJ, Shakhbazov K, Visscher PM. Calculating statistical power in Mendelian randomization studies. Int J Epidemiol. 2013;42(5):1497-501.

27. Jairath V, Khanna R, Zou GY, Stitt L, Mosli M, Vandervoort MK, et al. Development of interim patient-reported outcome measures for the assessment of ulcerative colitis disease activity in clinical trials. Aliment Pharmacol Ther. 2015;42(10):1200-10.

28. Khanna R, Zou G, D'Haens G, Feagan BG, Sandborn WJ, Vandervoort MK, et al. A retrospective analysis: the development of patient reported outcome measures for the assessment of Crohn's disease activity. Aliment Pharmacol Ther. 2015;41(1):77-86.

29. Lewis SJ, Heaton KW. Stool form scale as a useful guide to intestinal transit time. Scand J Gastroenterol. 1997;32(9):920-4.

30. Bodger K, Ormerod C, Shackcloth D, Harrison M, Collaborative IBDC. Development and validation of a rapid, generic measure of disease control from the patient's perspective: the IBD-control questionnaire. Gut. 2014;63(7):1092-102.

31. Cella D, Yount S, Rothrock N, Gershon R, Cook K, Reeve B, et al. The Patient-Reported Outcomes Measurement Information System (PROMIS): progress of an NIH Roadmap cooperative group during its first two years. Med Care. 2007;45(5 Suppl 1):S3-S11.

For peer review only

For peer review only - http://bmjopen.bmj.com/site/about/guidelines.xhtml

32. Herdman M, Gudex C, Lloyd A, Janssen M, Kind P, Parkin D, et al. Development and preliminary testing of the new five-level version of EQ-5D (EQ-5D-5L). Qual Life Res. 2011;20(10):1727-36.

33. Janssen MF, Pickard AS, Golicki D, Gudex C, Niewada M, Scalone L, et al. Measurement properties of the EQ-5D-5L compared to the EQ-5D-3L across eight patient groups: a multi-country study. Qual Life Res. 2013;22(7):1717-27.

34. Craig CL, Marshall AL, Sjostrom M, Bauman AE, Booth ML, Ainsworth BE, et al. International physical activity questionnaire: 12-country reliability and validity. Med Sci Sports Exerc. 2003;35(8):1381-95.

35. Lukas C, Landewe R, Sieper J, Dougados M, Davis J, Braun J, et al. Development of an ASAS-endorsed disease activity score (ASDAS) in patients with ankylosing spondylitis. Ann Rheum Dis. 2009;68(1):18-24.

36. Machado PM, Landewe R, Heijde DV, Assessment of SpondyloArthritis international S. Ankylosing Spondylitis Disease Activity Score (ASDAS): 2018 update of the nomenclature for disease activity states. Ann Rheum Dis. 2018;77(10):1539-40.

37. van der Heijde D, Lie E, Kvien TK, Sieper J, Van den Bosch F, Listing J, et al. ASDAS, a highly discriminatory ASAS-endorsed disease activity score in patients with ankylosing spondylitis. Ann Rheum Dis. 2009;68(12):1811-8.

38. Hollis JL, Craig LCA, Whybrow S, Clark H, Kyle JAM, McNeill G. Assessing the relative validity of the Scottish Collaborative Group FFQ for measuring dietary intake in adults. Public Health Nutrition. 2017;20(3):449-55.

For peer review only

For peer review only - http://bmjopen.bmj.com/site/about/guidelines.xhtml

WORD COUNT

4,363 words

AUTHOR STATEMENT

All authors contributed to clinical protocol and/or laboratory standard operating procedures. The grants to fund IBD-RESPONSE and CD-metaRESPONSE were conceptualised and written by CAL, LJ-D, CJS, MP, CA, NAK, TR, TA, AH, HH, CWL, JCM, JRM, NPo, NPr, JS, RAS. Clinical study design and analytical plans were further developed by these authors plus MB, EC, DC, MD, AK, CK, PMI, JOL, RL, SL, TL, RM, SS, TS, JW, KW, NJW, RW and XZ with critical review from all authors. Laboratory standard operating procedures were developed by CAL, CJS, HF, REM, LJ-D, CK, NAK, CA, MB, EC, DC, JAD, MD, SI, JOL, CCM, JRM, TO, MP, MS, NP, NJP, TR, MS, SS and NJW. Manuscript drafting was led by NJW and CAL with subsequent critical review and revision by all authors.

FUNDING

The IBD-RESPONSE cohort is supported by a grant from the Medical Research Council (funder reference MR/T032162/1) and the CD-metaRESPONSE cohort by, the Leona M. and Harry B. Helmsley Charitable Trust (funder reference 2002-04255). Single cell RNA sequencing in IBD-RESPONSE and CD-metaRESPONSE is supported by a grant from the Leona M. and Harry B. Helmsley Charitable Trust (funder reference 2304-05972). Cross-replication analysis utilising the Canada iGenoMed-MTT cohort us supported by a grant from Crohn's and Colitis UK (funder reference M2022-4 Lamb). CJS is supported by a Sir Henry Dale Fellowship jointly funded by the Wellcome Trust and the Royal Society (Grant Number 221745/Z/20/Z) and the 2021 Lister Institute Prize Fellow Award. NJW is supported by the NIHR Academic Clinical Fellowship (ACF) programme. LJD is supported by a Sir Henry Dale

For peer review only - http://bmjopen.bmj.com/site/about/guidelines.xhtml

Fellowship jointly funded by the Wellcome Trust and the Royal Society (Grant Number 208750/Z/17/Z) and the Kennedy Trust for Rheumatology Research. JMSW is funded by a NIHR Research Professorship (NIHR301614). XZ is funded by a NIHR Pre-doctoral Fellowship (NIHR302014).

ACKNOWLEDGEMENTS

We are grateful for support from the Newcastle Clinical Trials Unit, the National Phenome Centre, Imperial College London and the NIHR Biomedical Research Centres from Newcastle, Imperial and Cambridge. The views expressed are those of the authors and not necessarily those of our funders, the NIHR or the Department of Health and Social Care.

COMPETING INTERESTS STATEMENT

Tariq Ahmad reports personal grants from F. Hoffmann-La Roche AG, Biogen Inc, AbbVie, Janssen, Celltrion, Galapagos NV, Immunodiagnostik, and Takeda, outside the submitted work; personal fees for educational development/delivery from from Pfizer, payment or honoraria for lectures, presentations, speakers bureaus, manuscript writing or educational events from Pfizer, Takeda, and F. Hoffman-La Roche AG; support for attending meetings from Celltrion, Tillotts, and Pfizer. Carl A. Anderson reports grants from the Wellcome Sanger Institute Quinquennial Review 2021-2026, Crohn's and Colitis Foundation (USA), the Medical Research Council, Open Targets UK, and Helmsley Charitable Trust; consulting fees from BridgeBio Ltd, Genomics PLC, and Brigham & Women's Hospital Boston; payment or honoraria for lectures, presentations, speakers bureaus, manuscript writing or educational events from GlaxoSmithKline; support for attending meetings and/or travel membership from the Wellcome Sanger Institute Quinquennial Review 2021-2026; (Chair) of the Board of Trustees for the Sanger Prize; other interests as Director of Anderson Genomics Consultancy

For peer review only - http://bmjopen.bmj.com/site/about/guidelines.xhtml

Ltd. Michelle Bardgett reports partial personal salary funding from the Medical Research Council. Ailsa Hart reports personal consulting fees from AbbVie, BMS, Celltrion, Falk, Galapagos, Janssen, Pfizer, Takeda, and Roche; payment or honoraria for lectures, presentations, speakers bureaus, manuscript writing or educational events from BMS, Celltrion, Falk, Galapagos, Janssen, Pfizer, Takeda, Roche, and AbbVie; support for attending meetings and/or travel from BMS, Celltrion, Falk, Galapagos, Janssen, Pfizer, Takeda, Roche, and AbbVie. Peter M. Irving reports reports personal grants from Celltrion, Galapagos, and Pfizer, outside the submitted work; personal consulting fees from AbbVie, Takeda, BMS, Janssen, Arena, Pfizer, Galapagos, Lilly, Boehringer-Ingelheim, and Celgene; payment or honoraria for lectures, presentations, speakers bureaus, manuscript writing or educational events from AbbVie, Takeda, Janssen, Lilly, BMS, Pfizer, and Galapagos; support for attending meetings and/or travel from AbbVie and Tillotts. Luke Jostins-Dean reports grants from the Wellcome Trust, the Royal Society, the Kennedy Trust for Rheumatology Research, the Leona M. and Harry B. Helmsley Charitable Trust, and the Medical Research Council; grants from Novartis Pharmaceutical, outside the submitted work; consulting fees from Nightingale Health and Genomics PLC. Ciara Kennedy reports partial salary funding from the Medical Research Council. Nicholas A. Kennedy reports grants from AbbVie, Biogen, Celltrion, Galapagos, and Immunodiagnostik; consulting fees from AbbVie, Bristol-Meyer Squibb, and Dr Falk; payment or honoraria for lectures, presentations, speakers bureaus, manuscript writing or educational events from AbbVie, Dr Falk, Tillotts, Galapagos, and Takeda; support for attending meetings and/or travel from Tillotts; participation (Chair) on the Board of the British Society of Gastroenterology IBD Clinical Research Group. Christopher A. Lamb reports grants from Janssen, Takeda, AbbVie, AstraZeneca, Eli Lilly, Orion, Pfizer, Roche, Sanofi Aventis, UCB, Biogen, and Genentech Inc; payment or honoraria for lectures,

For peer review only - http://bmjopen.bmj.com/site/about/guidelines.xhtml

presentations, speakers bureaus, manuscript writing or educational events from Ferring, Takeda, Janssen, and Dr Falk; participation (Secretary) on the British Society of Gastroenterology IBD Section; participation on the Steering Committee of IBD UK. Charlie W. Lees reports grants from UKRI Future Leaders Fellowship; personal consulting fees from AbbVie, Pfizer, Janssen, Takeda, Galapagos, Fresnius Kabi, Novartis/Sandoz, BMS, and Celltrion; payment or honoraria for lectures, presentations, speakers bureaus, manuscript writing or educational events from AbbVie, Pfizer, Janssen, Takeda, Galapagos, Fresnius Kabi, Novartis/Sandoz, BMS, Ferring, Dr Falk, and Celltrion. James O. Lindsay reports grants from AbbVie, and Gilead; personal consulting fees from Allergan, AbbVie, Bristol Myers Squib, Celgene, Cornerstones US, Galapagos, Gilead, GSK, Lilly, MSD UK, Shire UK, Shire International, Ferring UK, Ferring International, Celltrion, Takeda, Pfizer, and Janssen; payment or honoraria for lectures, presentations, speakers bureaus, manuscript writing or educational events from AbbVie, Bristol Myers Squibb, Cornerstones US, Galapagos, Ferring UK, Ferring International, Celltrion, Takeda, Pfizer, and Janssen; support to attend meetings and/or travel from AbbVie, and Janssen. Rebecca H. Maier reports that she is an independent membership on the Trial Steering Committee for the National Institute for Health and Care Research funded ALLEGRO trial. Julian R. Marchesi reports personal consulting fees from EnteroBiotix Limited and Cultech Limited; patent held (without financial gain) on C. *Difficile* therapy (WO2019197836A1), participation (Chair) on the IDMC Board. Naomi McGregor reports partial (10%) salary funding from the Medical Research Council. Rebecca E. McIntyre reports personal salary funding from the Wellcome Sanger Institute. Jasmin Ostermayer reports stock held in Novartis AG. Miles Parkes reports grants from Pfizer and Gilead; personal consulting fees from Galapagos; payment or honoraria for lectures, presentations, speakers bureaus, manuscript writing or educational events from Janssen. Nick Powell

For peer review only - http://bmjopen.bmj.com/site/about/guidelines.xhtml

reports grants from Bristol-Myers Squibb, Takeda, and Pfizer; consulting fees from AbbVie, Allergan, Astra-Zeneca, Bristol-Myers Squibb, Celgene, Celltrion, Galapagos, GSK, Takeda, and Vifor; payment or honoraria for lectures, presentations, speakers bureaus, manuscript writing or educational events from AbbVie, Bristol-Myers Squibb, Ferring, Galapagos, Janssen, Roche, Pfizer, Takeda, and Tillotts; support for attending meetings and/or travel from AbbVie, Allergan, Celltrion, Janssen, and Takeda; participation on a data safety monitoring board or advisory board for AbbVie, Allergan, Astra-Zeneca, Bristol-Myers Squibb, Celgene, Celltrion, Galapagos, GSK, Takeda, and Vifor. Tim Raine reports personal grants from AbbVie; personal consulting fees from AbbVie, Arena, Aslan, AstraZeneca, Boehringer-Ingelheim, BMS, Celgene, Ferring, Galapagos, Gilead, GSK, Heptares, LabGenius, Janssen, Mylan, MSD, Novartis, Pfizer, Roche, Sandoz, Takeda, UCB, and XAP therapeutics; participation on the board of UCB, membership (Chair) of the ECCO Guidelines Committee, membership of the UEG Scientific Committee. Jack Satsangi reports grants from Crohn's and Colitis UK, Helmsley Trust, ECCO, the European Commission, CCFA, and Action Medical Research; payment or honoraria for lectures, presentations, speakers bureaus, manuscript writing or educational events from Roche; participation on a Data Safety Monitoring Board or Advisory Board for the MODULATE trial and the TRIBUTE trial; leadership or fiduciary role as the Director of the Royal College of Physicians IBD Registry, and Governing Body Fellow at Green Templeton College. R. Alexander Speight reports personal consulting fees from GSK; payment or honoraria for lectures, presentations, speakers bureaus, manuscript writing or educational events from Falk, and AbbVie; payment of conference fees to attend the British Society of Gastroenterology Annual Conference 2022 from Celltrion; participation on a Data Safety Monitoring Board or Advisory Board for the IBD-RESPONSE study (unpaid), and Abbvie; participation on the British Society of Gastroenterology IBD Section Committee.

For peer review only - http://bmjopen.bmj.com/site/about/guidelines.xhtml

Christopher J. Stewart reports personal consultancy fees from Astarte Medical; payment or honoraria for lectures, presentations, speakers bureaus, manuscript writing or educational events from Nestle Nutrition Institute. James M. S. Wason reports grants from Intercept; consulting fees from Worg and UCB; payment or honoraria for lectures, presentations, speakers bureaus, manuscript writing or educational events from Janssen; participation on a Data Safety Monitoring Board or Advisory Board for Roche. Kevin Whelan reports grants from the Leona M. and Harry B. Helsmsley Charitable Trust, Crohn's and Colitis UK, Almond Board of California, Danone, International Dried Fruit and Nut Council, Medical Research Council, National Institute for Health and care Research; royalty or license payments for Volatile organic compounds in the diagnosis and management of irritable bowel syndrome, and Wiley BDA Advances in Nutrition & Dietetics book series; personal consulting fees from Danone; payment or honoraria for lectures, presentations, speakers bureaus, manuscript writing or educational events from Janssen; support for attending meetings and/or travel from Yakult; participation on a Data Safety Monitoring Board or Advisory Board for the MODULATE trial (unpaid). The remaining authors disclose no conflicts of interest.

For peer review only

FIGURE LEGENDS

Figure 1. Study overview schematic (created with BioRender.com). 1,325 participants with IBD planned to commence an advanced therapy will be recruited, including a nested sub-cohort of 300 CD patients (CD-metaRESPONSE). All participants will collect two stool sample tubes at each study assessment timepoint (baseline, week 14 and week 54). CD-metaRESPONSE participants will be required to collect a third stool sample tube at baseline and week 14. If a participant attends hospital for a face-to-face appointment within the baseline and/or week 14 study assessment window, blood samples will be collected. If a participant attends hospital for a lower gastrointestinal endoscopy at any time during the study period (pre- or post- treatment), biopsy samples will be collected. Participants will complete several questionnaires at each assessment time point. For CD-metaRESPONSE participants, additional detailed analyses will be undertaken of metabolic profiles (metabolome) in stool and matched blood plus in-depth dietary assessment (additional elements highlighted in blue boxes). Data generated will be used to perform predictive modelling. Any remaining participant samples will form a large biorepository for use in future research.

Figure 1 abbreviations: anti-tumour necrosis factor alpha (anti-TNFα), anti-interleukin (anti-IL), Bristol stool form scale (BSFS), Crohn's disease (CD), formalin-fixed paraffin embedded (FFPE), inflammatory bowel disease (IBD), International Physical Activity Questionnaire (IPAQ), janus kinase inhibitor (JAKi), Joint Pain and Stiffness Score (JPSS), Patient Reported Outcome-2 (PRO-2), Patient Reported Outcomes Measurement Information System (PROMIS), sphingosine-1-phosphate receptor (S1PR), ulcerative colitis (UC).

Figure 2. Flowchart providing overview of study events.

For peer review only

Figure 2 abbreviations: Crohn's disease (CD), inflammatory bowel disease (IBD), Janus kinase inhibitor (JAKi), Mayo Clinic Score (MCS), multidisciplinary team (MDT), Patient Reported Outcome-2 (PRO-2), simple endoscopic score for Crohn's disease (SES-CD), sphingosine-1-phosphate receptor (S1PR), Research Electronic Data Capture (REDCap), ulcerative colitis (UC).

For peer review only

For peer review only - http://bmjopen.bmj.com/site/about/guidelines.xhtml

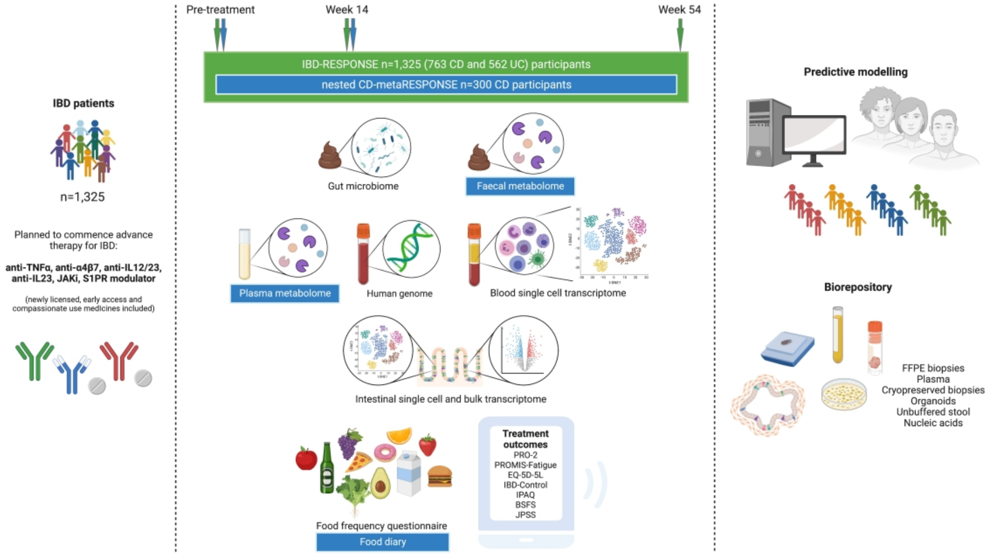

Figure 1. Study overview schematic (created with BioRender.com). 1,325 participants with IBD planned to commence an advanced therapy will be recruited, including a nested sub-cohort of 300 CD patients (CD-metaRESPONSE). All participants will collect two stool sample tubes at each study assessment timepoint (baseline, week 14 and week 54). CD-metaRESPONSE participants will be required to collect a third stool sample tube at baseline and week 14. If a participant attends hospital for a face-to-face appointment within the baseline and/or week 14 study assessment window, blood samples will be collected. If a participant attends hospital for a lower gastrointestinal endoscopy at any time during the study period (pre- or post-treatment), biopsy samples will be collected. Participants will complete several questionnaires at each assessment time point. For CD-metaRESPONSE participants, additional detailed analyses will be undertaken of metabolic profiles (metabolome) in stool and matched blood plus in-depth dietary assessment (additional elements highlighted in blue boxes). Data generated will be used to perform predictive modelling. Any remaining participant samples will form a large biorepository for use in future research.

Figure 1 abbreviations: anti-tumour necrosis factor alpha (anti-TNFα), anti-interleukin (anti-IL), Bristol stool form scale (BSFS), Crohn's disease (CD), formalin-fixed paraffin embedded (FFPE), inflammatory bowel disease (IBD), International Physical Activity Questionnaire (IPAQ), janus kinase inhibitor (JAKi), Joint Pain and Stiffness Score (JPSS), Patient Reported Outcome-2 (PRO-2), Patient Reported Outcomes Measurement Information System (PROMIS), sphingosine-1-phosphate receptor (S1PR), ulcerative colitis (UC).

400x228mm (300 x 300 DPI)

Potential participant identified

Where: Face-to-face clinics, telephone consultations, endoscopy lists, infusion suites, MDT meetings, virtual biologics clinics, IBD nurse helpline

Who: ≥16 years old; symptomatic, active luminal IBD; no minimum disease duration

Advanced therapy being commenced: Licensed biologic, JAKi or S1PR modulator (infliximab, adalimumab, vedolizumab, ustekinumab, tofacitinib, filgotinib, upadacitinib, ozanimod) or early access/compassionate use therapy (e.g. risankizumab)

Screening tips:

- UC symptoms: increased stool frequency relative to baseline AND at least one episode of recent rectal bleeding within e.g. last 3-5 days
- CD symptoms of active flare: presence of liquid or very soft stools AND/OR presence of abdominal pain
- Active disease confirmed by clinical test in 12 weeks prior to consent: faecal calprotectin, CRP, endoscopy or imaging (CD only)
- Participants do not need to be naïve to advanced therapies e.g. can previously have received a biologic
- Participants switching between advanced therapies do not need a minimum washout period
- Participants can be receiving or about to start concomitant thiopurine or methotrexate alongside planned advanced therapy
- Participants can be receiving or starting steroids at baseline provided likely to have fully weaned by week 14

Eligibility confirmed

Study information and informed consent given

- Participant Information Sheet (PIS) provided (paper or electronic format)
- Potential participant questions answered by local study team
- REDCap access given to participant to complete informed study consent

Consent verified

Stool sample collection kit given to/posted to participant once valid consent obtained and verified by local study team

Baseline assessment

Ideal: Week 0
Target: Up to 6 weeks prior to commencing advanced therapy
Permitted: Assessment >6 weeks prior to commencing advanced therapy is permitted, e.g. if delayed due to infusion unit capacity

All participants to complete after consent and before starting advanced therapy:

1. Participant questionnaires completed on REDCap
2. Stool sample tubes (n=2) collected and returned using prepaid Royal Mail Safebox™
3. If attending hospital for a face-to-face clinical encounter within assessment window, two tubes (total 20mls) blood collected

CD-metaRESPONSE sub-cohort (in addition to above requirements):

1. Third stool sample tube collected (provided in home stool sample collection kit)
2. Prospective 4-day food diary completed

Participants unable/unwilling to complete PRO-2 questionnaire, those not meeting PRO-2 threshold for clinically active disease, missing stool samples or who do not start treatment will be withdrawn from the study

Eligibility re-confirmed

Baseline 4 day PRO-2 (minimum 2 days data) assessed to confirm inclusion criteria for clinically active disease met

Biologic, JAKi or S1PR modulator commenced

WEEK 0

Week 14 assessment

Ideal: Week 14
Target: Week 12 - 16
Permitted: Week 10 - 20

As per baseline assessment (including additional requirements for CD-metaRESPONSE)

Week 54 assessment

Ideal: Week 54
Permitted: Week 48 - 60

All participants:

1. Participant questionnaires completed on REDCap
2. Stool sample tubes (n=2) collected and returned using prepaid Royal Mail Safebox™

If a participant stops treatment, the next planned study assessment should be brought forward and completed as a treatment discontinuation assessment. Where an alternative advanced therapy is then commenced, this discontinuation assessment will serve as the baseline assessment for the next therapy. Further assessments should then be completed at week 14 and 54 after commencing a new advanced therapy as per the study protocol

In addition to the above assessments, if a participant attends hospital for a planned lower gastrointestinal endoscopy at any time during the study period following consent, the following samples/data will be collected: up to 12 research biopsies, patient-reported 1-day PRO-2 score, endoscopic assessment of disease activity data (SES-CD/MCS endoscopic subscore)

BMJ Open

Defining predictors of responsiveness to advanced therapies in Crohn's disease and ulcerative colitis: Protocol for the IBD-RESPONSE and nested CD-metaRESPONSE prospective, multicentre, observational cohort study in precision medicine

Journal:	*BMJ Open*
Manuscript ID	bmjopen-2023-073639.R1
Article Type:	Protocol
Date Submitted by the Author:	14-Sep-2023
Complete List of Authors:	Wyatt, Nicola; Newcastle University Translational and Clinical Research Institute, Faculty of Medical Sciences; Newcastle Upon Tyne Hospitals NHS Foundation Trust, Department of Gastroenterology<br>Watson, Hannah; Newcastle University Translational and Clinical Research Institute, Faculty of Medical Sciences<br>Anderson, Carl ; Wellcome Sanger Institute, Wellcome Genome Campus<br>Kennedy, Nicholas A; Royal Devon University Healthcare NHS Foundation Trust, Department of Gastroenterology; University of Exeter Inflammatory Bowel Disease and Pharmacogenetics Research Group<br>Raine, Tim; Cambridge University Hospitals NHS Foundation Trust<br>Ahmad, T.; Royal Devon University Healthcare NHS Foundation Trust, Department of Gastroenterology; University of Exeter Inflammatory Bowel Disease and Pharmacogenetics Research Group<br>Allerton, Dean; Newcastle University, Newcastle Clinical Trials Unit<br>Bardgett, Michelle; Newcastle University, Newcastle Clinical Trials Unit<br>Clark, Emma; Newcastle University, Newcastle Clinical Trials Unit<br>Clewes, Dawn; Newcastle University, Newcastle Clinical Trials Unit<br>Cotobal Martin, Cristina; Wellcome Sanger Institute, Wellcome Genome Campus<br>Doona, Mary; Newcastle Upon Tyne Hospitals NHS Foundation Trust, Department of Gastroenterology<br>Doyle, Jennifer; Newcastle University Translational and Clinical Research Institute, Faculty of Medical Sciences<br>Hancock, Helen; Newcastle University, Newcastle Clinical Trials Unit<br>Hart, Ailsa L.; St Mark's Hospital and Academic Institute, Department of Gastroenterology; Imperial College London, Department of Surgery and Cancer<br>Irving, Peter; Guy's and St Thomas' NHS Foundation Trust, Department of Gastroenterology; King's College London, School of Immunology & Microbial Sciences<br>Iqbal, Sameena; Wellcome Sanger Institute, Wellcome Genome Campus<br>Kennedy, Ciara; Newcastle University, Newcastle Clinical Trials Unit<br>King, Andrew; Newcastle Upon Tyne Hospitals NHS Foundation Trust, Department of Gastroenterology<br>Lawrence, Sarah; Newcastle University, Newcastle Clinical Trials Unit<br>Lees, Charlie W.; The University of Edinburgh MRC Institute of Genetics and Molecular Medicine; Western General Hospital, Institute of Genetics

	& Molecular Medicine<br>Lees, Robert; Newcastle Upon Tyne Hospitals NHS Foundation Trust, Department of Gastroenterology<br>Letchford, Laura; Wellcome Sanger Institute, Wellcome Genome Campus<br>Liddle, Trevor; Newcastle Upon Tyne Hospitals NHS Foundation Trust<br>Lindsay, James ; Barts Health NHS Trust, Department of Gastroenterology; Blizard Institute of Cell and Molecular Science, Centre for Immunobiology<br>Maier, Rebecca; Newcastle University, Newcastle Clinical Trials Unit<br>Mansfield, John; Newcastle University Translational and Clinical Research Institute, Faculty of Medical Sciences; Newcastle Upon Tyne Hospitals NHS Foundation Trust, Department of Gastroenterology<br>Marchesi, Julian R.; St Mary's Hospital, Division of Digestive Diseases, Department of Metabolism, Digestion and Reproduction<br>McGregor, Naomi; Newcastle University, Newcastle Clinical Trials Unit<br>McIntyre, Rebecca ; Wellcome Sanger Institute, Wellcome Genome Campus<br>Ostermayer, Jasmin; Wellcome Sanger Institute, Wellcome Genome Campus<br>Osunnuyi, Tolulope; Wellcome Sanger Institute, Wellcome Genome Campus<br>Powell, Nick; St Mary's Hospital, Division of Digestive Diseases, Department of Metabolism, Digestion and Reproduction; Imperial College Healthcare NHS Trust, Division of Digestive Diseases, Department of Metabolism, Digestion and Reproduction<br>Prescott, Natalie; King's College London, Department of Medical and Molecular Genetics, Guy's Hospital<br>Satsangi, J; University of Oxford, Nuffield Department of Medicine<br>Sharma, Shriya; Newcastle University, Newcastle Clinical Trials Unit<br>Shrestha, Tara; Newcastle University, Newcastle Clinical Trials Unit<br>Speight, Ally; Newcastle University Translational and Clinical Research Institute; Newcastle Upon Tyne Hospitals NHS Foundation Trust, Department of Gastroenterology<br>Strickland, Michelle; Wellcome Sanger Institute, Wellcome Genome Campus<br>Wason, James; Newcastle University, Population Health Sciences Institute<br>Whelan, Kevin; King's College London, Department of Nutritional Sciences<br>Wood, Ruth; Newcastle University, Newcastle Clinical Trials Unit<br>Zhang, Xinyue; Newcastle University, Population Health Sciences Institute<br>Parkes, M; Cambridge University Hospitals NHS Foundation Trust, Department of Gastroenterology<br>Stewart, Christopher; Newcastle University Translational and Clinical Research Institute, Faculty of Medical Sciences<br>Jostins-Dean, Luke; University of Oxford, Kennedy Institute of Rheumatology<br>Lamb, Christopher; Newcastle University Translational and Clinical Research Institute, Faculty of Medical Sciences; Newcastle Upon Tyne Hospitals NHS Foundation Trust, Department of Gastroenterology
**Primary Subject Heading**:	Gastroenterology and hepatology
Secondary Subject Heading:	Pharmacology and therapeutics, Patient-centred medicine, Immunology (including allergy), Genetics and genomics, Diagnostics
Keywords:	Inflammatory bowel disease < GASTROENTEROLOGY, Gastroenterology < INTERNAL MEDICINE, MICROBIOLOGY, Patient-Centered Care, Patient Reported Outcome Measures

SCHOLARONE™
Manuscripts

Defining predictors of responsiveness to advanced therapies in Crohn's disease and ulcerative colitis: Protocol for the IBD-RESPONSE and nested CD-metaRESPONSE prospective, multicentre, observational cohort study in precision medicine

Nicola J. Wyatt[1,2], Hannah Fuller[1], Carl A. Anderson[3], Nicholas A. Kennedy[4,5], Tim Raine[6], Tariq Ahmad[4,5], Dean Allerton[7], Michelle Bardgett[7], Emma Clark[7], Dawn Clewes[7], Cristina Cotobal Martin[3], Mary Doona[2], Jennifer A. Doyle[1], Helen Hancock[7], Ailsa Hart[8,9], Peter M. Irving[10,11], Sameena Iqbal[3], Ciara Kennedy[7], Andrew King[2], Sarah Lawrence[7], Charlie W. Lees[12,13], Robert Lees[2], Laura Letchford[3], Trevor Liddle[14], James O. Lindsay[15,16], Rebecca H. Maier[7], John C. Mansfield[1,2], Julian R. Marchesi[17], Naomi McGregor[7], Rebecca E. McIntyre[3], Jasmin Ostermayer[3], Tolulope Osunnuyi[3], Nick Powell[17,18], Natalie J Prescott[19], Jack Satsangi[20], Shriya Sharma[7], Tara Shrestha[7], Ally Speight[1,2], Michelle Strickland[3], James M. S. Wason[21], Kevin Whelan[22], Ruth Wood[7], Xinyue Zhang[21], Miles Parkes[6], Christopher J. Stewart[*1], Luke Jostins-Dean[*23], Christopher A. Lamb[*1,2]

[*]Denotes co-senior authorship

Correspondence:

Dr Christopher Lamb, Translational & Clinical Research Institute, Faculty of Medical Sciences, Newcastle University, Newcastle upon Tyne, NE2 4HH. United Kingdom

christopher.lamb@newcastle.ac.uk

For peer review only - http://bmjopen.bmj.com/site/about/guidelines.xhtml

Affiliations:

1. Translational & Clinical Research Institute, Faculty of Medical Sciences, Newcastle University, Newcastle upon Tyne, United Kingdom
2. Department of Gastroenterology, The Newcastle upon Tyne Hospitals NHS Foundation Trust, Newcastle upon Tyne, United Kingdom
3. Wellcome Sanger Institute, Wellcome Genome Campus, Hinxton, Cambridgeshire, United Kingdom
4. Department of Gastroenterology, Royal Devon University Healthcare NHS Foundation Trust, Exeter, United Kingdom
5. Exeter Inflammatory Bowel Disease and Pharmacogenetics Research Group, University of Exeter, Exeter, United Kingdom
6. Cambridge University Hospitals NHS Foundation Trust, Cambridge, United Kingdom
7. Newcastle Clinical Trials Unit (NCTU), 1-4 Claremont Terrace, Newcastle University, Newcastle upon Tyne, NE2 4AE, United Kingdom
8. Department of Gastroenterology, St Marks Hospital and Academic Institute, Gastroenterology, London, United Kingdom
9. Department of Surgery and Cancer, Imperial College, London, United Kingdom
10. Department of Gastroenterology, Guy's & St Thomas' NHS Foundation Trust, London, United Kingdom
11. School of Immunology & Microbial Sciences, King's College London, London, United Kingdom
12. Institute of Genetics & Molecular Medicine, University of Edinburgh, Edinburgh, United Kingdom
13. Edinburgh IBD Unit, Western General Hospital, NHS Lothian, Edinburgh, United Kingdom

For peer review only

For peer review only - http://bmjopen.bmj.com/site/about/guidelines.xhtml

14. The Newcastle upon Tyne Hospitals NHS Foundation Trust, Newcastle upon Tyne. United Kingdom
15. Department of Gastroenterology, Barts Health NHS Trust, The Royal London Hospital, London, United Kingdom
16. Centre for Immunobiology, Blizard Institute, Barts and the London School of Medicine Queen Mary University of London, London, United Kingdom
17. Division of Digestive Diseases, Department of Metabolism, Digestion and Reproduction, St Mary's Hospital, Imperial College London, London, United Kingdom
18. Department of Gastroenterology, Imperial College Healthcare NHS Trust, London, United Kingdom
19. Department of Medical and Molecular Genetics, King's College London, Guy's Hospital, London, United Kingdom
20. Nuffield Department of Medicine, University of Oxford, Old Road Campus, Oxford, United Kingdom

    Department of Nutritional Sciences, King's College London, London, United Kingdom
21. Population Health Sciences Institute, Newcastle University, Newcastle upon Tyne, United Kingdom
22. Department of Nutritional Sciences, King's College London, United Kingdom
23. Kennedy Institute of Rheumatology, University of Oxford, Oxford, United Kingdom

Keywords:

Inflammatory bowel disease, Crohn disease, gastrointestinal microbiome, multiomics, precision medicine

ORCiDs (alphabetical):

For peer review only

Tariq Ahmad: https://orcid.org/0000-0002-6058-5528

Dean Allerton: https://orcid.org/0000-0003-0507-4343

Carl A. Anderson: https://orcid.org/0000-0003-1719-7009

Michelle Bardgett: https://orcid.org/0000-0003-1393-2010

Emma Clark: https://orcid.org/0000-0003-0065-1463

Dawn Clewes: https://orcid.org/0000-0001-9600-859X

Cristina Cotobal-Martin: https://orcid.org/0000-0002-5877-2228

Mary Doona: https://orcid.org/0000-0001-9533-3745

Jennifer A. Doyle: https://orcid.org/0000-0001-7042-3269

Hannah Fuller: https://orcid.org/0000-0002-7965-938X

Helen Hancock: https://orcid.org/0000-0002-1494-8551

Ailsa Hart: https://orcid.org/0000-0002-7141-6076

Peter M. Irving: https://orcid.org/0000-0003-0972-8148

Sameena Iqbal: https;//orcid.org/0000-0003-0926-593X

Luke Jostins-Dean: https://orcid.org/0000-0002-2475-3969

Ciara Kennedy: https://orcid.org/0000-0003-2987-0977

Nicholas A. Kennedy: https://orcid.org/0000-0003-4368-1961

Andrew King: https://orcid.org/0000-0002-5002-4751

Christopher A. Lamb: https://orcid.org/0000-0002-7271-4956

Sarah Lawrence: n/a

Charlie W. Lees: https://orcid.org/0000-0002-0732-8215

Robert Lees: https://orcid.org/0009-0003-7439-1681

Laura Letchford: n/a

Trevor Liddle: n/a

James O. Lindsay: https://orcid.org/0000-0003-3353-9590

Rebecca H. Maier: https://orcid.org/0000-0002-7350-3288

John C. Mansfield: https://orcid.org/0000-0003-2490-7750

Julian R. Marchesi: https://orcid.org/0000-0002-7994-5239

Naomi McGregor: https://orcid.org/0000-0002-5961-124X

Rebecca E. McIntyre: https://orcid.org/0000-0001-5291-1533

Jasmin Ostermayer: https://orcid.org/0000-0001-6461-398X

For peer review only

Tolulope Osunnuyi: https://orcid.org/0000-0002-0482-917X

Miles Parkes: https://orcid.org/0000-0002-6467-0631

Nick Powell: https://orcid.org/0000-0003-3231-6950

Natalie J. Prescott: https://orcid.org/0000-0002-5901-7371

Tim Raine: https://orcid.org/0000-0002-5855-9873

Jack Satsangi: https://orcid.org/0000-0002-6357-9684

Shriya Sharma: https://orcid.org/0000-0001-5237-757X

Tara Shrestha: n/a

Michelle Strickland: https://orcid.org/0000-0001-8053-400X

Ally Speight: https://orcid.org/0000-0003-3184-9181

Christopher J. Stewart: https://orcid.org/0000-0002-6033-338X

James M. S. Wason: https://orcid.org/0000-0002-4691-126X

Kevin Whelan: https://orcid.org/0000-0001-5414-2950

Ruth Wood: https://orchid.org/0000-0002-8296-1774

Nicola J. Wyatt: https://orcid.org/0000-0001-5050-327X

Xinyue Zhang: https://orcid.org/0000-0002-5321-4235

For peer review only

Abstract

INTRODUCTION

Characterised by chronic inflammation of the gastrointestinal tract, inflammatory bowel disease (IBD) symptoms including diarrhoea, abdominal pain and fatigue can significantly impact patient quality of life. Therapeutic developments in the last 20 years have revolutionised treatment. Despite these developments, clinical trials and real-world data show primary non-response rates up to 40%. A significant challenge is an inability to predict which treatment will benefit individual patients.

Current understanding of IBD pathogenesis implicates complex interactions between host genetics and the gut microbiome. Most cohorts studying the gut microbiota to date have been underpowered, examined single treatments, and produced heterogeneous results. Lack of cross-treatment comparisons and well-powered independent replication cohorts hampers the ability to infer real-world utility of predictive signatures.

IBD-RESPONSE will use multi-omic data to create a predictive tool for treatment response. Future patient benefit may include development of biomarker-based treatment stratification or manipulation of intestinal microbial targets. IBD-RESPONSE and downstream studies have the potential to improve quality of life, reduce patient risk, and reduce expenditure on ineffective treatments.

METHODS AND ANALYSIS

This prospective, multicentre, observational study will identify and validate a predictive model for response to advanced IBD therapies, incorporating gut microbiome, metabolome, single-cell transcriptome, human genome and clinical data. 1,325 participants commencing

For peer review only

advanced therapies will be recruited from ~40 UK sites. Data will be collected at baseline, week 14 and week 54. The primary outcome is week 14 clinical response. Secondary outcomes include clinical remission, loss of response in week 14 responders, corticosteroid-free response/remission, time to treatment escalation, and change in patient-reported outcome measures.

ETHICS AND DISSEMINATION

Ethical approval was obtained from the Wales Research Ethics Committee 5 (ref:21/WA/0228). Recruitment is ongoing. Following study completion, results will be submitted for publication in peer-reviewed journals and presented at scientific meetings. Publications will be summarised at www.ibd-response.co.uk.

TRIAL REGISTRATION NUMBER

ISRCTN96296121(pre-results)

Article Summary

STRENGTHS AND LIMITATIONS OF THIS STUDY

- IBD-RESPONSE is the largest precision medicine study of its kind to date, designed to develop a predictive tool for treatment response – or failure to respond – to IBD treatment, promoting *a priori* selection of the right drug, for the right patient, at the right time.
- IBD-RESPONSE will establish one of the largest biorepositories of longitudinal stool, blood, matched tissue and organoids with detailed linked phenotypic, diet, genetic and treatment outcome data in patients with IBD.

For peer review only - http://bmjopen.bmj.com/site/about/guidelines.xhtml

- This study will use a standardised yet pragmatic methodology for sample collection, processing and storage, allowing multicentre participation, improving generalisation and transferability of findings to real-world clinical practice.
- Remote ‘at home’ patient data and stool collection will allow >40 IBD centres to contribute to recruitment, increasing power in this multi-omics study when compared to other studies in the field that generally recruit from one or a small number of centres.
- Whilst recruiting sites are limited to the UK only, identified cross-validation cohorts from the UK and Canada will increase generalisability of our findings.

For peer review only

For peer review only - http://bmjopen.bmj.com/site/about/guidelines.xhtml

INTRODUCTION

Crohn's disease (CD) and ulcerative colitis (UC) are the principal forms of inflammatory bowel disease (IBD).(1) Characterised by symptoms including diarrhoea, rectal bleeding, abdominal pain and extra-intestinal features such as fatigue, IBD can have a substantial negative impact on patient quality of life.(2) Approximately 20% of CD patients and 10% of UC patients are unable to work due to their condition.(3) The global prevalence of IBD is rising. In the United Kingdom (UK), 1 in 125 people are affected, with prevalence expected to reach 1 in 100 by 2028.(4, 5) Outside of Western Europe and North America, the incidence is rising rapidly in many regions including South America, Latin America, Asia and Africa.(6, 7)

The biologics era has revolutionised IBD treatment in the last twenty years. Patients and clinicians have more advanced therapies to choose from than ever before. Several biologic classes are now licensed in the UK, targeting tumour necrosis factor alpha (TNFα) (including infliximab and adalimumab), interleukin (IL)-12/23 cytokine pathways (ustekinumab), or the gut-homing α4β7 integrin (vedolizumab). In addition, recently licensed small molecule therapies for UC include the Janus kinase inhibitors (JAKi) tofacitinib, filgotinib and upadacitinib, and the sphingosine-1-phosphate receptor (S1PR) modulator ozanimod. With several additional therapies in advanced stages of development or having completed phase 3 randomised clinical trials, the number of treatments available to patients is likely to increase.(8)

Current understanding of biological mechanisms driving the pathogenesis and natural history of IBD, implicates complex interactions between host genetics and the gut microbiome (bacteria, viruses, fungi, archaea and phage).(9) Whilst large clinical cohorts for human genetic discovery have led to major advances in understanding disease pathogenesis,(10)

For peer review only

cohorts for the study of gut microbiota have mostly been under-powered. Nonetheless, existing research intriguingly suggests utility of microbiome signatures in predicting response to therapy. In a small prospective study of 85 patients starting vedolizumab therapy, greater alpha-diversity and higher abundance of *Roseburia inulinivorans* and a Burkholderiales species at baseline were associated with therapeutic-induced remission in CD.(11) Incorporation of microbial taxonomy data alongside clinical data in a predictive model produced an area under the receiver operating characteristic curve (AUC) of 0.776 (compared to an AUC of 0.619 using clinical data only). A larger study of 232 patients receiving ustekinumab implicated *Bacteroides* and *Faecalibacterium* as predictors of treatment response.(12) Here, a predictive model of response to ustekinumab using clinical metadata produced an AUC of 0.616, rising to 0.844 when combined with baseline bacterial profile data.

Beyond IBD, further proof of concept that the gut microbiome is of prognostic importance in the context of systemically administered immune-targeted therapies is found with immune checkpoint inhibitor treatment in cancer, where experimental animal data demonstrated the beneficial impact of microbial modulation on treatment outcome.(13-15)

Metabolites derived from the gut microbiome are important intermediaries in the host-microbiome dialogue.(16) Specific classes of metabolites, such as bile acids (BA),short-chain fatty acids (SCFA) and tryptophan metabolites, may play a role in modulating disease activity and treatment responsiveness in IBD.(17) In a study of 185 (77 UC, 108 CD) patients commencing anti-cytokine (anti-TNFα or anti-IL12/23) or anti-integrin (vedolizumab) therapy, metabolomic and proteomic analysis of blood in addition to taxonomic and functional profiling of stool samples was conducted.(18) Among patients receiving anti-cytokine therapy, 120 enzymes were differentially abundant in baseline samples of remitters versus non-remitters. Single-species dominance (>50% of enzyme copies in >50% of samples explained by a single

For peer review only - http://bmjopen.bmj.com/site/about/guidelines.xhtml

species) was observed for 8/120 enzymes. *Eggerthella lenta* was dominant for 5 of these 8 enzymes, 3 of which are involved in secondary BA biosynthesis. Metabolomic analysis of baseline blood samples revealed significant enrichment of serum secondary BAs in patients achieving week 14 clinical remission. Paired baseline stool samples revealed a significant positive correlation with the abundance of 7α/β-dehydroxylation enzymes (responsible for primary to secondary BA conversion), presence of which was associated with a preferential response to anti-cytokine therapy. This was replicated by the authors in a small validation cohort of 46 patients initiating anti-TNFα (infliximab) therapy. In a small study of 29 patients with moderate-severe UC receiving vedolizumab treatment, untargeted metabolomic analysis of stool showed significantly higher levels of SCFAs including butyrate in those achieving week 14 remission (defined as total Mayo score ≤2, all subscores ≤1) versus non-remitters.(19) Combining metabolite data (SCFA levels for butyrate and isobutyric acid) with microbial profile data predicted anti-integrin response with an AUC of 0.961.

Whilst these previous studies are important first steps to utilising gut microbial signatures in stratified treatment algorithms, they were mostly underpowered, examined single treatments, used different sequencing technologies, and produced heterogeneous non-overlapping results. The lack of cross-treatment comparisons and well-powered independent replication cohorts hampers the ability to infer real-world utility of these predictive signatures, and to move from observations of association to causation in IBD.(20)

METHODS AND ANALYSIS

Study rationale

A significant challenge to effective, personalised use of biologic or small molecule therapies (collectively termed ‘advanced therapies’) in IBD is an inability to predict which class

For peer review only - http://bmjopen.bmj.com/site/about/guidelines.xhtml

of treatment is most likely to benefit an individual patient (see *Figure 1*). Despite increasing therapeutic options, clinical trial and real-world efficacy data show primary non-response rates of up to 40% across all therapeutic classes and in those with initial symptomatic benefit, up to 40% lose response by 1 year.(1, 21-23) Consequently, complications of chronic, active inflammation including strictures, fistulae and malignancy, continue to affect a substantial number of patients and have a negative impact on patient quality of life.(2) Up to 30% of CD patients require surgical intervention within 10 years of diagnosis, and around 15% of UC patients will ultimately require a colectomy.(24) Off-target side effects may also occur, including infection and malignancy.(1) With average treatment costs of £6,156/year for CD and £3,084/year for UC, future treatment algorithms must avoid the potential morbidity and additional cost associated with expensive treatments that do not benefit individual patients.(25)

The absolute importance of precision medicine research to identify biomarkers for treatment stratification and develop prognostic algorithms was highlighted by two recent national research prioritisation exercises incorporating responses from almost 3,000 patients, their families and friends.(1, 26) Validated prognostic models for treatment stratification do not exist and understanding of mechanisms controlling treatment non-response is limited. Through a multi-omic, precision medicine approach, the IBD-RESPONSE study seeks to improve selection of the right drug, for the right patient, at the right time. Other translational outputs of IBD-RESPONSE could bring into focus potential non-pharmacological approaches to treating IBD that do not necessarily involve large health economic expenditure. This could include manipulating the gut microbiome via the microbiota, through refinement of faecal microbial transplant protocols, use of pre- and probiotics, and dietary interventions.

For peer review only - http://bmjopen.bmj.com/site/about/guidelines.xhtml

Scientific objectives of IBD-RESPONSE

The primary scientific objective of IBD-RESPONSE is to identify and validate a predictive model for clinical response or failure to respond to advanced therapies in IBD after 14 weeks of therapy (the primary clinical outcome, see below). Modelling will incorporate gut microbiome, human genome, blood and intestinal single cell transcriptome data, and detailed clinical data. Through data derived from a nested sub-cohort (CD-metaRESPONSE), predictive modelling will also include detailed dietary information and blood and faecal metabolome data. The co-primary scientific objective of IBD-RESPONSE is to determine the relationship between clinical response and remission at week 14 and baseline gut microbiome.

Secondary scientific objectives of IBD-RESPONSE are to determine if there is a relationship between the microbiome at baseline or changes in the microbiome following advanced therapy with any of the secondary clinical outcomes (*Box 1*). Further exploratory scientific objectives of IBD-RESPONSE are listed in *Box 2.*

- Clinical remission at week 14.
- Clinical response at week 54.
- Clinical remission at week 54.
- Loss of response at week 54 in week 14 responders.
- Durable corticosteroid-free response or remission at week 54 defined as receiving no corticosteroids between week 14 and week 54 assessments inclusive and not meeting criteria for loss of response.
- Time to treatment escalation from baseline, defined as:
    - Advanced therapy switch due to lack of efficacy/those with loss of response (does not include biosimilar switch or switch from IV to SC route).

For peer review only

- Dose intensification of drug due to lack of efficacy (does not include intensification based on therapeutic drug monitoring without flare in responders).
- Resectional intestinal surgery (does not include examination under anaesthesia procedures in patients with perianal CD).
- Induction or dose escalation of corticosteroids.

- Time to treatment escalation as defined above, but disregarding dose intensification.
- Time to treatment escalation as defined above, among week 14 responders.
- Time to discontinuation of index drug (persistence).
- Incidence of and time to potential side effects of treatment during follow up.
- Continuation of drug at week 14 and/or week 54 in those not meeting criteria for response and/or remission.
- Change from baseline SF subscore at week 14 and/or 54 (both CD and UC).
- Change from baseline RB subscore at week 14 and/or 54 (UC only).
- Change from baseline AP subscore at week 14 and/or 54 (CD only).
- Development of anti-drug antibodies by week 14 or 54.
- Change in CRP from baseline at week 14 or 54 (50% reduction or absolute value ≤5mg/L deemed as clinically significant).
- Change in faecal calprotectin from baseline at week 14 or 54 (50% reduction or absolute value ≤100μg/g deemed as clinically significant).
- Endoscopic remission during follow up (Mayo endoscopic subscore ≤1 for UC or SES-CD ≤2 for CD).

For peer review only

For peer review only - http://bmjopen.bmj.com/site/about/guidelines.xhtml

- Change in quality of life, physical activity dietary intake, joint pain and fatigue as measured by study questionnaires.

Box 1. IBD-RESPONSE secondary clinical outcome measures

Box 1 abbreviations: abdominal pain (AP), Crohn's disease (CD), c-reactive protein (CRP), intravenous (IV), rectal bleeding (RB), subcutaneous (SC), stool frequency (SF), simple endoscopic score for Crohn's disease (SES-CD), ulcerative colitis (UC)

- Test the association of microbial metabolites (metabolome) in stool or plasma, human genetics and/or single cell transcriptome data from blood or intestinal tissue with the above primary and secondary objectives.
- Determine the influence of diet on the gut microbiome and treatment response in IBD, and the factors associated with dietary intake in IBD.
- Explore host human genetic-gut microbiome-metabolome interactions in IBD pathogenesis and causal pathways to treatment response.
- Ascertain the utility of archived endoscopy collected FFPE biopsies at predicting/imputing the gut microbiome and for inclusion in the predictive model.
- Establish a longitudinal tissue, organoid and stool biobank from this well characterised clinical cohort.

Box 2. IBD-RESPONSE exploratory scientific objectives

Box 2 abbreviations: inflammatory bowel disease (IBD), formalin fixed paraffin embedded (FFPE)

For peer review only

For peer review only - http://bmjopen.bmj.com/site/about/guidelines.xhtml

Study design

The design of IBD-RESPONSE and the nested CD-metaRESPONSE studies are summarised in *Figure 2*. This prospective, observational, multi-centre, cohort study will recruit participants with IBD (CD, UC, IBD-unclassified (IBD-U)) who are due to commence either biologic, JAKi or S1PR modulator therapy for symptomatic, clinically active (moderate to severe) luminal disease. Participants do not have to be naïve to advanced therapies and may be recruited when switching within or between class of advance therapy. Participants may be taking or planned to start concurrent thiopurines or methotrexate as combination therapy. Participation in the study will not change standard clinical care received. Detailed longitudinal clinical data will be collected alongside stool, blood and (where possible) biopsy samples, patient reported outcome measures, and dietary intake. Planned recruitment of 1,325 participants will consist of 762 patients with CD and 563 patients with UC (or IBD-U).

Data will be collected at baseline (prior to starting treatment), week 14 (following completion of induction therapy) and week 54. Participants will collect stool samples at each study timepoint. If a participant attends hospital within the baseline and/or week 14 study window, two blood samples (per timepoint) will be collected. Where a participant undergoes endoscopy as part of routine clinical care during the study period, up to 12 research biopsies will be collected.

Of 762 CD participants, 300 will be consented to take part in the nested CD-metaRESPONSE sub-cohort. Inclusion criteria are identical to the main cohort. Clinical data collection will occur at the same time points as in the main cohort. Additional study components will include completion of a 4-day food diary questionnaire, capturing all food and drink consumed at the baseline and week 14 timepoints. This is in addition to the food

For peer review only - http://bmjopen.bmj.com/site/about/guidelines.xhtml
For peer review only

frequency questionnaire completed by all participants. CD-metaRESPONSE participants will also be required to provide two additional stool tubes (one at baseline and one at week 14) for faecal metabolome analysis. Participants will be recruited to CD-metaRESPONSE from a limited number of the participating sites. These sites will initially preferentially recruit eligible participants with CD to CD-metaRESPONSE. Once the recruitment target of 300 participants is achieved, all new participants identified with a diagnosis of CD will be recruited to the main cohort.

All participants recruited to IBD-RESPONSE will be invited to co-recruit to IBD BioResource (if not already participating). IBD BioResource is a national platform and recallable biorepository linked to the National Institute for Health and Care Research (NIHR) BioResource that is designed to expedite IBD research, currently with >36,000 participants.(27) Co-recruiting participants to IBD-RESPONSE and the IBD BioResource platform, will generate a rich dataset and provide a long term means of archiving data from IBD-RESPONSE to facilitate ongoing research and maximise downstream patient benefit. To minimise participant burden, the contact for recruitment to IBD BioResource can occur any time in the 12 months following consent to IBD-RESPONSE. If a participant ultimately decides not to participate in the IBD BioResource they will not be withdrawn from IBD-RESPONSE.

If a participant discontinues treatment prior to week 14 or week 54 follow up, the next timepoint assessment will be brought forward and completed as a treatment discontinuation assessment. Where a participant does not respond to the first prescribed advanced therapy and a second (or third) advanced therapy is subsequently prescribed, this discontinuation assessment will act as the baseline sample and data collection assessment for the successive advanced therapy. Follow up samples and data collection will occur at week 14 and week 54 following commencement of each successive agent. The participant may remain in the study

For peer review only - http://bmjopen.bmj.com/site/about/guidelines.xhtml

up to 54 weeks after commencement of a particular agent or until the end of the study period. We anticipate that up to 40% of patients will not respond to the initial prescribed therapy or will lose response by 1 year and will move on to a second (or third) advanced therapy. We therefore anticipate that recruitment of 1,325 participants may capture approximately 1,760 new treatment initiation episodes.

Study setting

This multi-centre cohort study will be conducted at >40 study centres, based within National Health Service (NHS) Trusts across the UK. All sites must be able to accommodate the needs of the IBD-RESPONSE cohort including clinical engagement, research nurse support and facilities for assessments.

Eligibility criteria

Individuals will be deemed eligible to enrol in the study if they fulfil all inclusion criteria and meet none of the exclusion criteria (*Box 3*).

For peer review only - http://bmjopen.bmj.com/site/about/guidelines.xhtml

INCLUSION CRITERIA

Individuals must fulfil <u>all the following criteria</u> to be enrolled in the study.

- Aged 16 years and over.
- Diagnosis of IBD: CD, UC or IBD-U.*
- Already participating or willing to be approached for participation in IBD BioResource.
- Willing and able to provide informed consent.
- Willing to undertake study procedures including:
  - Completion of study questionnaires
  - Collection of home stool specimens
- Provision of blood and (where applicable) biopsy specimens.
- Symptoms of active luminal IBD (see definition of 'Clinically active disease', *Table 1*).
- At least one biochemical, endoscopic or radiological marker of active disease within 16 weeks of study consent (see *Box 4* and *Box 5*)
- Intention of clinical team to commence one of the following advanced therapies for active luminal IBD within 6 weeks of consent:△
  - Infliximab
  - Adalimumab
  - Vedolizumab
  - Ustekinumab
  - Tofacitinib
  - Filgotinib
  - Upadacitinib
  - Ozanimod

*Individuals with IBD-U will be managed as per the UC relevant protocol.

△Participants may be advanced therapy-naïve or -exposed. Any new biologic or small molecule drug that becomes licensed for the treatment of IBD during the planned study period will be permitted to allow study inclusion. Drugs used through Early Access to Medicines Schemes, compassionate use or expanded access schemes for unlicensed therapies are also permitted. Patients starting immunosuppressant monotherapy with a thiopurine or methotrexate are not eligible to take part. However, use of these treatments as part of combination therapy with an advanced therapy is not an exclusion to enrolment.

For peer review only - http://bmjopen.bmj.com/site/about/guidelines.xhtml

EXCLUSION CRITERIA

Individuals meeting any of the following criteria will not be eligible to participate in the study.

- Receiving oral corticosteroids for any indication where the dose is unlikely to be weaned by week 14.^
- Planned bowel resection surgery within 14 weeks of commencing therapy.
- Advanced therapy being commenced as rescue for ASUC.
- Advanced therapy being commenced as part of a CTIMP.
- Presence of an ileal pouch anal anastomosis.
- Presence of a stoma.
- Perianal CD in the absence of active luminal inflammation.
- Antibiotics or short-term (≤4 weeks) use of probiotics within the preceding 2 weeks.†
- FMT within the preceding 12 weeks or planned FMT within 14 weeks of commencing advanced therapy for IBD.‡

^Examples may include long term steroids for IBD where weaning by 14 weeks may not be possible irrespective of response to advanced therapy, or concurrent diagnosis where long term steroids are used e.g. polymyalgia rheumatica.

†Use of long-term (>4 weeks), stable doses of probiotics does not exclude individual participation but should be noted in the eCRF.

‡Use of antibiotics or prior FMT outside the exclusion period is permitted. Antibiotic use in the preceding 1 year and ever having received FMT will be noted in the eCRF.

Box 3. Eligibility criteria for IBD-RESPONSE

Box 3 abbreviations: Acute severe ulcerative colitis (ASUC), Clinical Trial of an Investigational Medicinal Product (CTIMP), Crohn's disease (CD), electronic case report form (eCRF), faecal microbial transplantation (FMT), inflammatory bowel disease (IBD), inflammatory bowel disease-unclassified (IBD-U), ulcerative colitis (UC).

Participants must have at least one biochemical, endoscopic or radiological (CD only) marker of clinically active luminal disease within 16 weeks of study consent (see *Box 4* (CD) and *Box 5* (UC)). Endoscopic assessment of disease activity will be judged locally and may

For peer review only - http://bmjopen.bmj.com/site/about/guidelines.xhtml

be assessed prospectively or retrospectively. Whilst pregnancy may influence advance therapy selection, pregnancy is not an exclusion criterion.

CD patients must also have at least one of the following documented within 16 weeks prior to consent:

- Faecal calprotectin ≥250 μg/g.
- CRP ≥6 mg/L.
- Any endoscopic evidence of active CD, defined as ulceration (with at least one ulcer ≥5mm) judged locally from available clinical data (as an approximation equivalent to SES-CD of ≥4 for ileal disease or ≥6 for ileocolonic or colonic disease.
- Active inflammatory disease on imaging (MRI/CT/ultrasound) judged locally from available clinical data.

Box 4. IBD-RESPONSE and CD-metaRESPONSE additional inclusion criteria: CD

Box 4 abbreviations: Crohn's disease (CD), C-reactive protein (CRP), Computed tomography (CT), Magnetic resonance imaging (MRI), Simple Endoscopic Score for Crohn's Disease (SES-CD)

UC/IBD-U patients must also have at least one of the following documented within 16 weeks prior to consent:

- Faecal calprotectin ≥250 μg/g.
- CRP ≥6 mg/L.
- Any endoscopic evidence of at least moderately active ulcerative colitis (of any extent including proctitis), defined as features of MCS endoscopic sub-score ≥2 (marked erythema, lack of vascular pattern, friability, erosions, spontaneous bleeding or ulceration.

Box 5. IBD-RESPONSE additional inclusion criteria: UC and IBD-U

For peer review only - http://bmjopen.bmj.com/site/about/guidelines.xhtml

Box 5 abbreviations: C-reactive protein (CRP), inflammatory bowel disease-unclassified (IBD-U), Mayo Clinic Score (MCS), ulcerative colitis (UC)

Clinical outcome measures

Key definitions related to clinical outcome measures can be found in *Table 1*.

Clinically active disease	• CD: Unweighted PRO-2 (CD) of average daily SF subscore ≥4 and/or average daily AP subscore ≥2.<br>• UC: Total PRO-2 (UC) ≥3 with RB subscore ≥1.<br>PRO-2 data will be entered by patients over 4 days (minimum 2 days PRO-2 data is permissible for PRO-2 calculation).
Clinical remission	Patient remains on drug and meets the following criteria:<br>• CD: Unweighted PRO-2 (CD) average daily SF subscore ≤2.8 and average daily AP subscore ≤1 (and neither worse than inclusion scores at baseline).<br>• UC: PRO-2 (UC) SF subscore ≤1 with a decrease of ≥1 point(s) in SF subscore from baseline, plus RB subscore = 0.<br>**AND** an absence of any of the following at time of assessment:<br>• Resectional bowel surgery at any time after baseline until time of current assessment.<br>• Current use of oral corticosteroids/failure to wean oral corticosteroids prescribed at baseline.
Clinical response	Meeting criteria for clinical remission **OR**:<br>• CD: unweighted PRO-2 (CD) ≥30% reduction in average daily SF subscore and/or ≥30% decrease in average daily AP subscore (and neither worse than inclusion scores at baseline).<br>• UC: total PRO-2 (UC) decrease ≥3 from baseline.

For peer review only - http://bmjopen.bmj.com/site/about/guidelines.xhtml

	**AND** an absence of any of the following at time of assessment:<br>• Resectional bowel surgery at any time after baseline until time of current assessment.<br>• Current use of oral corticosteroids/failure to wean oral corticosteroids prescribed at baseline.
Week 14 non-response	• Not meeting clinical response criteria AND not having stopped drug for any reason other than lack of efficacy.
Week 54 loss of response	• Not meeting clinical response criteria at week 54 having met clinical response criteria at week 14 (AND not having stopped drug for any reason other than inefficacy between week 14 and 54 assessments).

Table 1. IBD-RESPONSE key clinical definitions

Table 1 abbreviations: abdominal pain (AP), Crohn's disease (CD), patient reported outcome (PRO)-2, rectal bleeding (RB), stool frequency (SF), ulcerative colitis (UC).

For peer review only

Primary clinical outcome measures

Clinical response at week 14 after commencing therapy.

Secondary clinical outcome measures

Secondary clinical outcome measures are listed in *Box 1*.

Sample size calculations

The sample size (n=1,325 cases overall, including n=300 cases for CD-metaRESPONSE sub-cohort) was chosen to ensure sufficient power to answer the co-primary scientific objective (to detect associations between microbiome measures and clinical response or remission) and key exploratory scientific objectives (to detect associations between metabolites and clinical response or remission, and to detect associations between genetics and microbiome measures). Sample size calculations for predictive models require

For peer review only - http://bmjopen.bmj.com/site/about/guidelines.xhtml

extensive assumptions about the number and effect size of associations and the correlation structure of the data. We noted predictive models built with microbiome and/or metabolite measures with high in-sample predictive accuracy (including AUC=0.78 with n=84 from Ananthakrishnan et al.(11), AUC=0.91 with n=76 from Ding et al.(28), and AUC=0.84 with n=232 from the CERTIFI study(12)), implying that n=300 individuals (from CD-metaRESPONSE) and n=1,325 individuals (from IBD-RESPONSE), should be sufficient for high predictive in-sample accuracy.

Sample size for analysis of primary objective

We took plausible effect sizes for the primary scientific objective analysis from the effect of *Bacteroides* levels in stool on ustekinumab response (d=0.66, from the CERTIFI study(12)) and the effect of anti-drug immunogenicity on remission after anti-TNFα treatment (d=0.30, from the PANTS study(21)). We calculated power for a simple two-sample t-test (using the R package pwr), assuming the expected non-response rate at 14 weeks (23.8%) and non-remission rate at 54 weeks (63.1%) from the PANTS study. We assumed a conservative Bonferroni-corrected significance threshold of 1e-5 (correcting for 5000 microbiome measures). We calculated the minimum sample size required to achieve 80% power for the two effect sizes and two outcomes (*Supplementary Figure 1*), showing that relatively low sample sizes are required to have high power to detect the larger plausible effect size (n=282 for remission and n=361 for response), but that larger sample sizes are required to have high power to detect the smaller plausible effect size (n=1,331 for remission and n=1,705 for response). The sample size of n=1,325 chosen for our study gives a power of close to 100% for the larger *Bacteroides* effect size for both week 14 response and week 54 remission, and 57% and 80% for the smaller anti-drug immunogenicity effect size for week 14 response and week 54 remission, respectively.

For peer review only - http://bmjopen.bmj.com/site/about/guidelines.xhtml

Sample size for analysis of secondary and exploratory scientific objectives

We took plausible effect sizes for the exploratory metabolite analysis from the effects of three selected stool lipid and bile acid metabolites on anti-TNFα response from Ding et al.(28): faecal triglyceride (d=1.00), and two bile acid metabolites; BA1 (d=0.89) and BA3 (d=0.70). We assumed a conservative Bonferroni-corrected significance threshold of 5e-5 (correcting for 1000 metabolites). For n=300, this gives a power of 99.7%, 91% and 74% for faecal triglyceride, BA1 and BA3 respectively for week 14 response, and 100%, 99.8% and 91% for week 54 remission.

We do not anticipate that this study on its own will be well powered to detect new associations between genotype and microbiome measures in IBD. It is known from studies of healthy individuals that genetic variants that explain more than 3% of variation (R^2=0.03) in microbial abundance are rare.(29) Assuming a conservative significance threshold of < 1e-11 (correcting for 1e6 independent genotypes and 5000 microbiome measures), n=1,325 samples would only have 33% power to detect associations with R^2=0.03 (calculated using genpwr(30)). We will therefore combine our samples with a further genotype/microbiome study of IBD (PREdiCCt) to increase sample size to n=2,325. This will provide us 80% power to detect genetic associations with R^2>0.025. In the case where such genotype/microbiome measure associations exist, and are associated with a causal biomarker for week 54 remission, a Mendelian Randomization analysis would have >80% power to demonstrate causality of this biomarker when the causal effect odds ratios is larger than 2 (calculated using mRnd(31)).

For peer review only - http://bmjopen.bmj.com/site/about/guidelines.xhtml

Study procedures and measures

Participants ≥16 years old may be identified from a variety of settings such as outpatient clinics (face-to-face or virtual), flare assessments, IBD clinical nurse specialist helplines/email contact, endoscopy examinations, infusion suites, multidisciplinary team meetings and virtual biologics clinics (*Figure 2*). Consent will be taken electronically using a REDCap (Research Electronic Data Capture) online database. Full informed e-consent will be supported by an appropriately delegated member of the study team, using a laptop, tablet or mobile device in the patient's own home, or using a hospital tablet, laptop, computer or patient mobile device during a scheduled visit to hospital as part of routine clinical care. Paper copies of the consent form will be made available for those patients unable to access e-consent. If a later decision is made not to commence an advanced therapy for IBD, the participant will not be eligible to continue in the study and must be withdrawn. Data collected up to the point of withdrawal may be used for the study. Any samples collected will be used for research within IBD-RESPONSE or stored for future research.

All research activity will be completed by the participant either remotely or during hospital visits scheduled to deliver routine clinical care (see *Figure 3*). Participants will be asked to complete data collection after consent and before starting treatment (baseline), and at week 14 and week 54 following commencement of advanced therapy in line with routine dosing schedule visits. This will include patient questionnaires, stool samples and, where applicable, blood and biopsy specimens. Participants will be asked to complete questionnaires with data entered directly into the study specific REDCap database. Paper questionnaires will be made available for those participants without access or who express a preference to complete in paper format. Participants will be asked to complete questionnaires related to

For peer review only - http://bmjopen.bmj.com/site/about/guidelines.xhtml

disease activity, health, physical activity, quality of life and diet at the three assessment timepoints (see *Box 6*).

Participant samples

All sample collection and processing will be standardised, with full requirements detailed in a study Sample Collection Manual.

Stool samples will be collected by participants at home using stool collection kits and returned using a prepaid Royal Mail Safebox™. All participants will collect two stool samples per study assessment timepoint (DNA Genotek OMNIgene®•GUT tube and universal polystyrene tube). Participants recruited to the CD-metaRESPONSE sub-cohort will be required to collect a third stool sample at baseline and week 14 (DNA Genotek OMNImet®•GUT tube). DNA extraction and metagenomic shotgun sequencing will be performed on buffered samples. Calprotectin will be measured in unbuffered stool. Remaining fresh stool and nucleic acids will be cryopreserved for use in future research.

Participants who attend a clinical appointment prior to commencing advanced therapy or within the week 14 visit window (week 10-20; week 12-16 preferred) will be asked to provide two blood samples (lithium heparin (LiH) tube and ethylenediaminetetra-acetic acid (EDTA) tube). Blood samples will be utilised for single cell analysis, plasma extraction and cryopreservation. Any remaining blood samples or derivatives will be stored at Newcastle Biobank for use in future research. Participants will not be asked to attend hospital specifically for blood sample collection. If participants are not scheduled to attend hospital face-to-face within either study assessment window, blood samples will not be taken.

If a participant has a lower gastrointestinal endoscopy as part of planned care during study participation, up to 12 research biopsies (one set of six biopsies to be collected from the

For peer review only

colon in all participants and a further set of six biopsies from the ileum in CD participants) will be taken. Where a participant meets the study eligibility criteria and a disease assessment endoscopy is planned prior to starting therapy, consent should be received ahead of their planned endoscopy to enable the collection of research biopsies. Biopsy samples will be utilised for single cell sequencing and organoid generation. 16S rRNA gene sequencing of both FFPE and fresh tissue will also be undertaken. Any remaining biopsy samples will be stored for use in future research.

[Figure 3]

COMPLETED BY ALL PARTICIPANTS AT BASELINE, WEEK 14 AND WEEK 54
**PRO-2 (CD or UC version depending on diagnosis)**<br>PRO-2 is a validated questionnaire measuring patient reported outcomes including stool frequency, abdominal pain and rectal bleeding.(32, 33)<br>**Bristol Stool Form Scale (BSFS)**<br>The BSFS is a 7-point scale that helps describe stool shape and consistency and assess bowel patterns and habits.(34)<br>**IBD-Control**<br>The IBD-Control questionnaire comprises 13 items plus a visual analogue scale (VAS) ranging from 0–100.(35) The questionnaire measures patient related outcome of their disease state during the past 2 weeks.<br>**PROMIS-Fatigue 8a Short Form**<br>The Patient Reported Outcomes Measurement Information System (PROMIS), are validated questionnaires that help evaluate patients' quality of life.(36)<br>**EQ-5D-5L**<br>The EQ-5D-5L is a quality-of-life questionnaire and is a widely used generic PROM incorporating five domains: 1) mobility, 2) self-care, 3) usual activities, 4) pain/discomfort,

For peer review only - http://bmjopen.bmj.com/site/about/guidelines.xhtml

5) anxiety/depression.(37, 38) Scores for each domain are combined to describe the patient's state of health.

International Physical Activity Questionnaire (IPAQ)

IPAQ is a commonly used self-reported questionnaire to estimate physical activity and sedentary behaviours for adults across a range of socio-economic settings.(39) The IPAQ measures the type of physical activities people do as part of their everyday lives.(39)

IBD-RESPONSE Joint Pain and Stiffness Score (JPSS)

The Ankylosing Spondylitis Disease Activity Score (ASDAS) is a patient reported questionnaire which quantifies clinical disease activity in Ankylosing Spondylitis (AS) and combines five disease activity variables (four 10-point Likert scale patient symptom responses and a CRP measurement), to produce a single score.(40-42) We believe joint pain to be an under-recognised symptom in active inflammatory bowel disease which may change in response to therapy as inflammation resolves/fails to resolve and so we have modified the ASDAS to assess joint pain, swelling and stiffness in all IBD-RESPONSE patients irrespective of whether they have a rheumatological diagnosis. We have called this modified score the IBD-RESPONSE Joint Pain and Stiffness Score (JPSS).

Food frequency questionnaire (FFQ)

The FFQ will be completed by participants directly into the study specific REDCap database and can also be completed on paper. The FFQ requests information on 175 food items, their typical portion size and frequency of consumption and has been extensively validated for measuring nutrient intakes in adults.(43) Data are converted to nutrient intake using the Composition of Foods Integrated Dataset (COFIDs), as well as diet quality indices and other food components (e.g. polyphenols) and food categorisation (e.g. ultra-processed foods). The FFQ data will initially be analysed at the Centre for Healthcare Randomised Trials (CHaRT), University of Aberdeen and other collaborating institutions at the Department of Nutritional Sciences, King's College London.

For peer review only

COMPLETED BY CD-METARESPONSE PARTICIPANTS AT BASELINE AND WEEK 14 ONLY
**4-day food diaries**<br>The 4-day food diary measures current food intake. It will comprehensively and prospectively measure all intake allowing calculation of energy and nutrient intake, dietary indices (e.g. diet diversity and Mediterranean diet), intake of ultra-processed foods, prebiotic and emulsifier intake.
Box 6. Summary of patient questionnaires completed throughout study period

Statistical analysis

The analysis approach for our primary objective will be to test for the association between features of the patient microbiome at baseline and primary clinical response to treatment at 14 weeks. Features will include alpha diversity, abundance of bacterial taxa (including species, genus and phyla) as well as the abundance of genes within various microbial pathways (for example using KEGG pathways, MetaCyc metabolic pathways and gene families). Association testing will be carried out using negative binomial regression, controlling for total sequence depth and pre-defined technical and clinical confounders, and significance will be determined using Benjamini-Hochberg multiple testing correction to ensure a false discovery rate of less than 5%.

For the predictive modelling aim, we will use a random forest classifier to predict primary response to treatment at 14 weeks using microbiome, host genetic and clinical features at baseline. Model parameters will be tuned and accuracy assessed using nested cross-validation. This full model will be compared to a clinical-variables-only model, with improvement measured by area under the received operator curve, as well as positive and

For peer review only - http://bmjopen.bmj.com/site/about/guidelines.xhtml

negative predictive power. A further model will be fitted using the same approach including metabolomic and dietary data on the CD-metaRESPONSE subset. Our primary predictive measure will be reported for a random forest classifier, but a further sensitivity analysis will be carried out by fitting alternative prediction models to test whether this has a strong effect on the predictive accuracy, using both simpler models (including logistic regression with a LASSO penalty) and other more advanced methods (such as neural networks and support vector machines), The results of the predictive models will be reported in future publications according to the TRIPOD guidelines.(44)

Secondary and exploratory objective analyses will use the same general analysis approach as described above. For time-dependent events, such as treatment escalation due to loss of response, a Cox proportional hazards regression will be used to assess the impact of microbiome features on time to event, with patients censured at 54 weeks, last recorded (if lost to follow-up) or date of withdrawal (if withdrawn). The dietary data will be analysed to test the association between primary response and measures of specific nutrients (such as dietary fibre) and dietary indices will be used to assess adherence to certain recommended diets (such as achievement of food-based dietary guidelines or a "Mediterranean diet"). Host genotype data will be used to test for associations between primary response and generate polygenic risk scores of susceptibility to Crohn's disease and ulcerative colitis, as well as pre-specified variants associated with response to therapy (including HLA-DQA1*05). In all these individual analyses, Benjamini-Hochberg will be applied used to control the false discovery rate at 5%.

Loss to follow-up and missing data will be handled during the analyses in different ways depending on the specific question being addressed. For the primary analysis at 14 weeks, individuals who are lost to follow-up or withdraw from the study before 14 weeks will

For peer review only - http://bmjopen.bmj.com/site/about/guidelines.xhtml

be removed from the analysis, though we will also carry out a robustness analysis where we include individuals lost to follow-up as non-responders to ensure the results are robust to this choice. Secondary and exploratory analyses at specific time-points will be treated in the same fashion. For time-dependent events, analysed using survival models, individuals who are lost to follow-up or withdraw will be treated as censured at this time point (the point of withdrawal for withdrawn participants, and the last point of contact for patients lost to follow-up), though we will also carry out sensitivity analyses where these are instead treated as adverse outcomes where appropriate (e.g. treating withdrawals as adverse events). For missing data, standard quality control criteria for microbiome, metabolome and genetic data will be used to remove variables with excessive missing data (as well as other markers of poor data quality). Where data is missing for microbiome or other experimental assays for specific individuals after QC, only participants with non-missing data for this variable will be analysed. When constructing and validating predictive models, individuals with missing data for the predictive variables being tested, or that have withdrawn or been lost to follow-up before the assessment time, will be excluded from model building and testing. Statistical analysis will be carried out in R.

Replication

Scaling up microbiome discoveries and providing validation of results is needed to benefit patients. Whilst IBD-RESPONSE is the largest project of its kind to date, it is a UK-only programme. Translating findings to maximise patient benefit requires international collaboration. Standardised data collection and data integration from different countries and ethnic groups is key to establishing predictors of treatment response that are generalisable and replicable.

For peer review only

We will validate our predictive model using an appropriate, already assembled microbiome validation cohort. To generate this replication cohort, we will use banked stool DNA from the Prognostic effect of Environmental factors in Crohn's and Colitis Study (PREdiCCt; www.predicct.co.uk), led by Professor Charlie Lees. PREdiCCt is a prospective observational study of IBD participants in clinical remission, designed to identify whether baseline factors (including genetics, dietary habits and gut microbiota) predict subsequent disease flare. We will perform metagenomic sequencing of 1,000 stool samples from patients who experienced a disease flare during the PREdiCCt study. We expect approximately 40% of PREdiCCt patients to experience a disease flare requiring commencement of biologics. As these patients all have baseline (clinical remission) stool microbiome samples in storage, they provide a well matched and cost-efficient set of samples for replicating IBD-RESPONSE results.

We will also carry out a collaborative cross-replication analysis of results from IBD-RESPONSE and the Montreal, Canada iGenoMed-MTT cohort, led by Professor John Rioux to evaluate how well microbiome associations and predictive algorithms replicate across continents. This will involve analysis of stored stool samples from 105 Canadian IBD patients collected prior to commencing advanced therapies (following the sequencing and analysis protocols established in IBD-RESPONSE) with treatment outcome data. It will provide a rapid "first-look" analysis to discover and assess microbiome predictors of biological response in an international cohort with extensive metadata, ahead of larger studies coming online over a longer timeframe within IBD-RESPONSE and allow later cross-replication/meta-analysis.

For peer review only - http://bmjopen.bmj.com/site/about/guidelines.xhtml

Potential future benefit to patients

IBD-RESPONSE will provide timely and important information regarding associations between the gut microbiome and responsiveness to treatment in IBD. It will likely highlight potential mechanisms through which the microbiota may drive inflammation. We hope that findings from IBD-RESPONSE will lead to new personalised avenues for IBD treatment through discovery and validation of predictive tools that may be incorporated directly into clinical practice or further tested in stratified clinical trials. This could lead to the development of experimental techniques to modify gut microbes; for example, donor selection for faecal microbial transplantation, identification of single or multiple strains of microbes, or use of antimicrobials, phage or microbial metabolites that may be used to induce a more 'treatment responsive' microbiome.

Patient and public involvement statement

IBD-RESPONSE was informed by two national patient research prioritisation exercises in IBD care led by members of our team and involving feedback from 3,000 people living with IBD, their family and friends.(1, 26) These identify aspects of precision medicine, microbiome and diet in IBD as of high importance. The initial IBD-RESPONSE grant proposal to the Medical Research Council (MRC) was reviewed by the NIHR Research Design Service North East and North Cumbria Patient and Public Involvement Panel and was presented and discussed at the 2020 Crohn's & Colitis UK Patient and Public Involvement in Research Day. IBD-RESPONSE has been supported by two patient representatives in the Study Oversight Committee (SOC) since inception. The study team have engaged with lay members of the SOC to ensure all patient facing documents including the participant information sheet (PIS), consent forms, stool collection guidance for patients and study questionnaires have

For peer review only - http://bmjopen.bmj.com/site/about/guidelines.xhtml

undergone review. Patients will also be involved in dissemination activities relating to outputs from this research.

ETHICS AND DISSEMINATION

Ethical approval for the study was obtained from the Wales Research Ethics Committee 5 (reference 21/WA/0228). Recruitment to IBD-RESPONSE began in February 2022 and is currently ongoing at sites around the UK.

In line with the Newcastle University and The Newcastle upon Tyne Hospitals NHS Foundation Trust research data policy, datasets will be kept for at least five years after the date they were last accessed. Metadata linked to genomic and metagenomic datasets will include anonymised clinical information. Examples include diagnosis (UC/CD/IBD-U), disease location, disease behaviour, complications, extra-intestinal manifestations, co-morbidities, family history, smoking history, surgical interventions, and outcomes from prior drug therapies.

Raw data files in the original format (e.g. fastq) and the accompanying anonymised phenotypic data will be uploaded to a public repository e.g. the NCBI database of Genotypes and Phenotypes (dbGaP) at https://www.ncbi.nlm.nih.gov/gap/.

As part of CD-metaRESPONSE, microbial sequence and faecal/serum metabolomic data will be integrated with single-cell RNA sequencing, human genomics and clinical outcome data; the whole dataset will be made available to other investigators and will be archived long term within the IBD BioResource to facilitate downstream research.

The CI, SMG, Sponsor, Funders and research team members are committed to ensure that the research findings are shared. Findings will be written up and submitted to a peer-reviewed scientific journal. Findings will be presented by the study team at national and international conferences for example the British Society of Gastroenterology (BSG) annual

For peer review only - http://bmjopen.bmj.com/site/about/guidelines.xhtml

meeting, the European Crohn's and Colitis (ECCO) meeting and Digestive Diseases Week (DDW). The study team will prepare a lay summary of the study findings for dissemination to the study participants and members of the national patient group, Crohn's & Colitis UK. Following study completion, results will be submitted for publication in peer-reviewed journals and presented at national and international scientific meetings.

REGISTRATION DETAILS

Trial registration number: ISRCTN96296121 (pre-results).

FUNDING

The IBD-RESPONSE cohort is supported by a grant from the Medical Research Council (funder reference MR/T032162/1) and the CD-metaRESPONSE cohort by The Leona M. and Harry B. Helmsley Charitable Trust (funder reference 2002-04255). Single cell RNA sequencing in IBD-RESPONSE and CD-metaRESPONSE is supported by a grant from the Helmsley Charitable Trust (funder reference 2304-05972). Cross-replication analysis utilising the Canada iGenoMed-MTT cohort us supported by a grant from Crohn's and Colitis UK (funder reference M2022-4 Lamb). CJS is supported by a Sir Henry Dale Fellowship jointly funded by the Wellcome Trust and the Royal Society (Grant Number 221745/Z/20/Z) and the 2021 Lister Institute Prize Fellow Award. NJW is supported by the NIHR Academic Clinical Fellowship (ACF) programme. LJD is supported by a Sir Henry Dale Fellowship jointly funded by the Wellcome Trust and the Royal Society (Grant Number 208750/Z/17/Z) and the Kennedy Trust for Rheumatology Research. JMSW is funded by a NIHR Research Professorship (NIHR301614). XZ is funded by a NIHR Pre-doctoral Fellowship (NIHR302014).

For peer review only - http://bmjopen.bmj.com/site/about/guidelines.xhtml

ACKNOWLEDGEMENTS

We are grateful for support from the Newcastle Clinical Trials Unit, the National Phenome Centre, Imperial College London and the NIHR Biomedical Research Centres from Newcastle, Imperial and Cambridge. The views expressed are those of the authors and not necessarily those of our funders, the NIHR or the Department of Health and Social Care.

CONTRIBUTORSHIP STATEMENT

All authors contributed to clinical protocol and/or laboratory standard operating procedures. The grants to fund IBD-RESPONSE and CD-metaRESPONSE were conceptualised and written by CAL, LJ-D, CJS, MP, CA, NAK, TR, TA, AH, HH, CWL, JCM, JRM, NPo, NPr, AS, JS. Clinical study design and analytical plans were further developed by these authors plus MB, EC, DC, MD, AK, CK, PMI, JOL, RL, SL, TL, RM, SS, TS, JW, KW, NJW, RW and XZ with critical review from all authors. Laboratory standard operating procedures were developed by CAL, CJS, HF, REM, LJ-D, CK, NAK, CA, MB, EC, DC, JAD, MD, SI, JOL, CCM, JRM, TO, MP, MS, NP, NJP, TR, MS, SS and NJW. Manuscript drafting was led by NJW and CAL with subsequent critical review and revision by all authors.

COMPETING INTERESTS

Tariq Ahmad reports personal grants from F. Hoffmann-La Roche AG, Biogen Inc, AbbVie, Janssen, Celltrion, Galapagos NV, Immunodiagnostik, and Takeda, outside the submitted work; personal fees for educational development/delivery from from Pfizer, payment or honoraria for lectures, presentations, speakers bureaus, manuscript writing or educational events from Pfizer, Takeda, and F. Hoffman-La Roche AG; support for attending meetings from Celltrion, Tillotts, and Pfizer. Carl A. Anderson reports grants from the Wellcome Sanger Institute Quinquennial Review 2021-2026, Crohn's and Colitis Foundation (USA), the Medical

For peer review only - http://bmjopen.bmj.com/site/about/guidelines.xhtml

Research Council, Open Targets UK, and the Helmsley Charitable Trust; consulting fees from BridgeBio Ltd, Genomics PLC, and Brigham & Women's Hospital Boston; payment or honoraria for lectures, presentations, speakers bureaus, manuscript writing or educational events from GlaxoSmithKline; support for attending meetings and/or travel membership from the Wellcome Sanger Institute Quinquennial Review 2021-2026; (Chair) of the Board of Trustees for the Sanger Prize; other interests as Director of Anderson Genomics Consultancy Ltd. Michelle Bardgett reports partial personal salary funding from the Medical Research Council. Ailsa Hart reports personal consulting fees from AbbVie, BMS, Celltrion, Falk, Galapagos, Janssen, Pfizer, Takeda, and Roche; payment or honoraria for lectures, presentations, speakers bureaus, manuscript writing or educational events from BMS, Celltrion, Falk, Galapagos, Janssen, Pfizer, Takeda, Roche, and AbbVie; support for attending meetings and/or travel from BMS, Celltrion, Falk, Galapagos, Janssen, Pfizer, Takeda, Roche, and AbbVie. Peter M. Irving reports reports personal grants from Celltrion, Galapagos, and Pfizer, outside the submitted work; personal consulting fees from AbbVie, Takeda, BMS, Janssen, Arena, Pfizer, Galapagos, Lilly, Boehringer-Ingelheim, and Celgene; payment or honoraria for lectures, presentations, speakers bureaus, manuscript writing or educational events from AbbVie, Takeda, Janssen, Lilly, BMS, Pfizer, and Galapagos; support for attending meetings and/or travel from AbbVie and Tillotts. Luke Jostins-Dean reports grants from the Wellcome Trust, the Royal Society, the Kennedy Trust for Rheumatology Research, the Helmsley Charitable Trust, and the Medical Research Council; grants from Novartis Pharmaceutical, outside the submitted work; consulting fees from Nightingale Health and Genomics PLC. Ciara Kennedy reports partial salary funding from the Medical Research Council. Nicholas A. Kennedy reports grants from AbbVie, Biogen, Celltrion, Galapagos, and Immunodiagnostik; consulting fees from AbbVie, Bristol-Meyer Squibb, and Dr Falk; payment

For peer review only - http://bmjopen.bmj.com/site/about/guidelines.xhtml

or honoraria for lectures, presentations, speakers bureaus, manuscript writing or educational events from AbbVie, Dr Falk, Tillotts, Galapagos, and Takeda; support for attending meetings and/or travel from Tillotts; participation (Chair) on the Board of the British Society of Gastroenterology IBD Clinical Research Group. Christopher A. Lamb reports grants from Janssen, Takeda, AbbVie, AstraZeneca, Eli Lilly, Orion, Pfizer, Roche, Sanofi Aventis, UCB, Biogen, and Genentech Inc; payment or honoraria for lectures, presentations, speakers bureaus, manuscript writing or educational events from Ferring, Takeda, Janssen, and Dr Falk; participation (Secretary) on the British Society of Gastroenterology IBD Section; participation on the Steering Committee of IBD UK. Charlie W. Lees reports grants from UKRI Future Leaders Fellowship; personal consulting fees from AbbVie, Pfizer, Janssen, Takeda, Galapagos, Fresnius Kabi, Novartis/Sandoz, BMS, and Celltrion; payment or honoraria for lectures, presentations, speakers bureaus, manuscript writing or educational events from AbbVie, Pfizer, Janssen, Takeda, Galapagos, Fresnius Kabi, Novartis/Sandoz, BMS, Ferring, Dr Falk, and Celltrion. James O. Lindsay reports grants from AbbVie, and Gilead; personal consulting fees from Allergan, AbbVie, Bristol Myers Squib, Celgene, Cornerstones US, Galapagos, Gilead, GSK, Lilly, MSD UK, Shire UK, Shire International, Ferring UK, Ferring International, Celltrion, Takeda, Pfizer, and Janssen; payment or honoraria for lectures, presentations, speakers bureaus, manuscript writing or educational events from AbbVie, Bristol Myers Squibb, Cornerstones US, Galapagos, Ferring UK, Ferring International, Celltrion, Takeda, Pfizer, and Janssen; support to attend meetings and/or travel from AbbVie, and Janssen. Rebecca H. Maier reports that she is an independent membership on the Trial Steering Committee for the National Institute for Health and Care Research funded ALLEGRO trial. Julian R. Marchesi reports personal consulting fees from EnteroBiotix Limited and Cultech Limited; patent held (without financial gain) on C. *Difficile* therapy

For peer review only - http://bmjopen.bmj.com/site/about/guidelines.xhtml

(WO2019197836A1), participation (Chair) on the IDMC Board. Naomi McGregor reports partial (10%) salary funding from the Medical Research Council. Rebecca E. McIntyre reports personal salary funding from the Wellcome Sanger Institute. Jasmin Ostermayer reports stock held in Novartis AG. Miles Parkes reports grants from Pfizer and Gilead; personal consulting fees from Galapagos; payment or honoraria for lectures, presentations, speakers bureaus, manuscript writing or educational events from Janssen. Nick Powell reports grants from Bristol-Myers Squibb, Takeda, and Pfizer; consulting fees from AbbVie, Allergan, Astra-Zeneca, Bristol-Myers Squibb, Celgene, Celltrion, Galapagos, GSK, Takeda, and Vifor; payment or honoraria for lectures, presentations, speakers bureaus, manuscript writing or educational events from AbbVie, Bristol-Myers Squibb, Ferring, Galapagos, Janssen, Roche, Pfizer, Takeda, and Tillotts; support for attending meetings and/or travel from AbbVie, Allergan, Celltrion, Janssen, and Takeda; participation on a data safety monitoring board or advisory board for AbbVie, Allergan, Astra-Zeneca, Bristol-Myers Squibb, Celgene, Celltrion, Galapagos, GSK, Takeda, and Vifor. Tim Raine reports personal grants from AbbVie; personal consulting fees from AbbVie, Arena, Aslan, AstraZeneca, Boehringer-Ingelheim, BMS, Celgene, Ferring, Galapagos, Gilead, GSK, Heptares, LabGenius, Janssen, Mylan, MSD, Novartis, Pfizer, Roche, Sandoz, Takeda, UCB, and XAP therapeutics; participation on the board of UCB, membership (Chair) of the ECCO Guidelines Committee, membership of the UEG Scientific Committee. Jack Satsangi reports grants from Crohn's and Colitis UK, the Helmsley Charitable Trust, ECCO, the European Commission, CCFA, and Action Medical Research; payment or honoraria for lectures, presentations, speakers bureaus, manuscript writing or educational events from Roche; participation on a Data Safety Monitoring Board or Advisory Board for the MODULATE trial and the TRIBUTE trial; leadership or fiduciary role as the Director of the Royal College of Physicians IBD Registry, and Governing Body Fellow

For peer review only

For peer review only - http://bmjopen.bmj.com/site/about/guidelines.xhtml

at Green Templeton College. Ally Speight reports personal consulting fees from GSK; payment or honoraria for lectures, presentations, speakers bureaus, manuscript writing or educational events from Falk, and AbbVie; payment of conference fees to attend the British Society of Gastroenterology Annual Conference 2022 from Celltrion; participation on a Data Safety Monitoring Board or Advisory Board for the IBD-RESPONSE study (unpaid), and Abbvie; participation on the British Society of Gastroenterology IBD Section Committee. Christopher J. Stewart reports personal consultancy fees from Astarte Medical; payment or honoraria for lectures, presentations, speakers bureaus, manuscript writing or educational events from Nestle Nutrition Institute. James M. S. Wason reports grants from Intercept; consulting fees from Worg and UCB; payment or honoraria for lectures, presentations, speakers bureaus, manuscript writing or educational events from Janssen; participation on a Data Safety Monitoring Board or Advisory Board for Roche. Kevin Whelan reports grants from the Helmsmley Charitable Trust, Crohn's and Colitis UK, Almond Board of California, Danone, International Dried Fruit and Nut Council, Medical Research Council, National Institute for Health and care Research; royalty or license payments for Volatile organic compounds in the diagnosis and management of irritable bowel syndrome, and Wiley BDA Advances in Nutrition & Dietetics book series; personal consulting fees from Danone; payment or honoraria for lectures, presentations, speakers bureaus, manuscript writing or educational events from Janssen; support for attending meetings and/or travel from Yakult; participation on a Data Safety Monitoring Board or Advisory Board for the MODULATE trial (unpaid). The remaining authors disclose no conflicts of interest.

For peer review only

REFERENCES

1. Lamb CA, Kennedy NA, Raine T, Hendy PA, Smith PJ, Limdi JK, et al. British Society of Gastroenterology consensus guidelines on the management of inflammatory bowel disease in adults. Gut. 2019;68(Suppl 3):s1-s106.

2. Floyd DN, Langham S, Severac HC, Levesque BG. The economic and quality-of-life burden of Crohn's disease in Europe and the United States, 2000 to 2013: a systematic review. Dig Dis Sci. 2015;60(2):299-312.

3. van der Valk ME, Mangen MJ, Leenders M, Dijkstra G, van Bodegraven AA, Fidder HH, et al. Risk factors of work disability in patients with inflammatory bowel disease--a Dutch nationwide web-based survey: work disability in inflammatory bowel disease. J Crohns Colitis. 2014;8(7):590-7.

4. Jones GR, Lyons M, Plevris N, Jenkinson PW, Bisset C, Burgess C, et al. IBD prevalence in Lothian, Scotland, derived by capture-recapture methodology. Gut. 2019;68(11):1953-60.

5. Hamilton B, Green H, Heerasing N, Hendy P, Moore L, Chanchlani N, et al. Incidence and prevalence of inflammatory bowel disease in Devon, UK. Frontline Gastroenterology. 2021;12(6):461.

6. GBD 2017 Inflammatory Bowel Disease Collaborators. The global, regional, and national burden of inflammatory bowel disease in 195 countries and territories, 1990-2017: a systematic analysis for the Global Burden of Disease Study 2017. Lancet Gastroenterol Hepatol. 2020;5(1):17-30.

7. Ng SC, Shi HY, Hamidi N, Underwood FE, Tang W, Benchimol EI, et al. Worldwide incidence and prevalence of inflammatory bowel disease in the 21st century: a systematic review of population-based studies. Lancet. 2017;390(10114):2769-78.

8. Wyatt NJ, Speight RA, Stewart CJ, Kirby JA, Lamb CA. Targeting Leukocyte Trafficking in Inflammatory Bowel Disease. BioDrugs. 2021;35(5):473-503.

9. Jostins L, Ripke S, Weersma RK, Duerr RH, McGovern DP, Hui KY, et al. Host-microbe interactions have shaped the genetic architecture of inflammatory bowel disease. Nature. 2012;491(7422):119-24.

10. Luo Y, de Lange KM, Jostins L, Moutsianas L, Randall J, Kennedy NA, et al. Exploring the genetic architecture of inflammatory bowel disease by whole-genome sequencing identifies association at ADCY7. Nat Genet. 2017;49(2):186-92.

11. Ananthakrishnan AN, Luo C, Yajnik V, Khalili H, Garber JJ, Stevens BW, et al. Gut Microbiome Function Predicts Response to Anti-integrin Biologic Therapy in Inflammatory Bowel Diseases. Cell Host Microbe. 2017;21(5):603-10 e3.

12. Doherty MK, Ding T, Koumpouras C, Telesco SE, Monast C, Das A, et al. Fecal Microbiota Signatures Are Associated with Response to Ustekinumab Therapy among Crohn's Disease Patients. MBio. 2018;9(2).

13. Routy B, Le Chatelier E, Derosa L, Duong CPM, Alou MT, Daillere R, et al. Gut microbiome influences efficacy of PD-1-based immunotherapy against epithelial tumors. Science. 2018;359(6371):91-7.

14. Matson V, Fessler J, Bao R, Chongsuwat T, Zha Y, Alegre ML, et al. The commensal microbiome is associated with anti-PD-1 efficacy in metastatic melanoma patients. Science. 2018;359(6371):104-8.

15. Gopalakrishnan V, Spencer CN, Nezi L, Reuben A, Andrews MC, Karpinets TV, et al. Gut microbiome modulates response to anti-PD-1 immunotherapy in melanoma patients. Science. 2018;359(6371):97-103.

For peer review only - http://bmjopen.bmj.com/site/about/guidelines.xhtml

16. Lavelle A, Sokol H. Gut microbiota-derived metabolites as key actors in inflammatory bowel disease. Nature Reviews Gastroenterology & Hepatology. 2020;17(4):223-37.

17. Fiorucci S, Carino A, Baldoni M, Santucci L, Costanzi E, Graziosi L, et al. Bile Acid Signaling in Inflammatory Bowel Diseases. Dig Dis Sci. 2021;66(3):674-93.

18. Lee JWJ, Plichta D, Hogstrom L, Borren NZ, Lau H, Gregory SM, et al. Multi-omics reveal microbial determinants impacting responses to biologic therapies in inflammatory bowel disease. Cell Host & Microbe. 2021;29(8):1294-304.e4.

19. Liu J, Fang H, Hong N, Lv C, Zhu Q, Feng Y, et al. Gut Microbiome and Metabonomic Profile Predict Early Remission to Anti-Integrin Therapy in Patients with Moderate to Severe Ulcerative Colitis. Microbiol Spectr. 2023;11(3):e0145723.

20. Plichta DR, Graham DB, Subramanian S, Xavier RJ. Therapeutic Opportunities in Inflammatory Bowel Disease: Mechanistic Dissection of Host-Microbiome Relationships. Cell. 2019;178(5):1041-56.

21. Kennedy NA, Heap GA, Green HD, Hamilton B, Bewshea C, Walker GJ, et al. Predictors of anti-TNF treatment failure in anti-TNF-naive patients with active luminal Crohn's disease: a prospective, multicentre, cohort study. Lancet Gastroenterol Hepatol. 2019;4(5):341-53.

22. Narula N, Peerani F, Meserve J, Kochhar G, Chaudrey K, Hartke J, et al. Vedolizumab for Ulcerative Colitis: Treatment Outcomes from the VICTORY Consortium. Am J Gastroenterol. 2018;113(9):1345.

23. Wils P, Bouhnik Y, Michetti P, Flourie B, Brixi H, Bourrier A, et al. Subcutaneous Ustekinumab Provides Clinical Benefit for Two-Thirds of Patients With Crohn's Disease Refractory to Anti-Tumor Necrosis Factor Agents. Clin Gastroenterol Hepatol. 2016;14(2):242-50 e1-2.

24. Tsai L, Ma C, Dulai PS, Prokop LJ, Eisenstein S, Ramamoorthy SL, et al. Contemporary Risk of Surgery in Patients With Ulcerative Colitis and Crohn’s Disease: A Meta-Analysis of Population-Based Cohorts. Clinical Gastroenterology and Hepatology. 2021;19(10):2031-45.e11.

25. Ghosh N, Premchand P. A UK cost of care model for inflammatory bowel disease. Frontline Gastroenterol. 2015;6(3):169-74.

26. Hart AL, Lomer M, Verjee A, Kemp K, Faiz O, Daly A, et al. What Are the Top 10 Research Questions in the Treatment of Inflammatory Bowel Disease? A Priority Setting Partnership with the James Lind Alliance. J Crohns Colitis. 2017;11(2):204-11.

27. Parkes M, Investigators IBDB. IBD BioResource: an open-access platform of 25 000 patients to accelerate research in Crohn's and Colitis. Gut. 2019;68(9):1537-40.

28. Ding NS, McDonald JAK, Perdones-Montero A, Rees DN, Adegbola SO, Misra R, et al. Metabonomics and the Gut Microbiome Associated With Primary Response to Anti-TNF Therapy in Crohn's Disease. J Crohns Colitis. 2020;14(8):1090-102.

29. Bonder MJ, Kurilshikov A, Tigchelaar EF, Mujagic Z, Imhann F, Vila AV, et al. The effect of host genetics on the gut microbiome. Nat Genet. 2016;48(11):1407-12.

30. Moore CM, Jacobson SA, Fingerlin TE. Power and Sample Size Calculations for Genetic Association Studies in the Presence of Genetic Model Misspecification. Hum Hered. 2019;84(6):256-71.

31. Brion MJ, Shakhbazov K, Visscher PM. Calculating statistical power in Mendelian randomization studies. Int J Epidemiol. 2013;42(5):1497-501.

32. Jairath V, Khanna R, Zou GY, Stitt L, Mosli M, Vandervoort MK, et al. Development of interim patient-reported outcome measures for the assessment of ulcerative colitis disease activity in clinical trials. Aliment Pharmacol Ther. 2015;42(10):1200-10.

For peer review only - http://bmjopen.bmj.com/site/about/guidelines.xhtml

33. Khanna R, Zou G, D'Haens G, Feagan BG, Sandborn WJ, Vandervoort MK, et al. A retrospective analysis: the development of patient reported outcome measures for the assessment of Crohn's disease activity. Aliment Pharmacol Ther. 2015;41(1):77-86.

34. Lewis SJ, Heaton KW. Stool form scale as a useful guide to intestinal transit time. Scand J Gastroenterol. 1997;32(9):920-4.

35. Bodger K, Ormerod C, Shackcloth D, Harrison M, Collaborative IBDC. Development and validation of a rapid, generic measure of disease control from the patient's perspective: the IBD-control questionnaire. Gut. 2014;63(7):1092-102.

36. Cella D, Yount S, Rothrock N, Gershon R, Cook K, Reeve B, et al. The Patient-Reported Outcomes Measurement Information System (PROMIS): progress of an NIH Roadmap cooperative group during its first two years. Med Care. 2007;45(5 Suppl 1):S3-S11.

37. Herdman M, Gudex C, Lloyd A, Janssen M, Kind P, Parkin D, et al. Development and preliminary testing of the new five-level version of EQ-5D (EQ-5D-5L). Qual Life Res. 2011;20(10):1727-36.

38. Janssen MF, Pickard AS, Golicki D, Gudex C, Niewada M, Scalone L, et al. Measurement properties of the EQ-5D-5L compared to the EQ-5D-3L across eight patient groups: a multi-country study. Qual Life Res. 2013;22(7):1717-27.

39. Craig CL, Marshall AL, Sjostrom M, Bauman AE, Booth ML, Ainsworth BE, et al. International physical activity questionnaire: 12-country reliability and validity. Med Sci Sports Exerc. 2003;35(8):1381-95.

40. Lukas C, Landewe R, Sieper J, Dougados M, Davis J, Braun J, et al. Development of an ASAS-endorsed disease activity score (ASDAS) in patients with ankylosing spondylitis. Ann Rheum Dis. 2009;68(1):18-24.

41. Machado PM, Landewe R, Heijde DV, Assessment of SpondyloArthritis international S. Ankylosing Spondylitis Disease Activity Score (ASDAS): 2018 update of the nomenclature for disease activity states. Ann Rheum Dis. 2018;77(10):1539-40.

42. van der Heijde D, Lie E, Kvien TK, Sieper J, Van den Bosch F, Listing J, et al. ASDAS, a highly discriminatory ASAS-endorsed disease activity score in patients with ankylosing spondylitis. Ann Rheum Dis. 2009;68(12):1811-8.

43. Hollis JL, Craig LCA, Whybrow S, Clark H, Kyle JAM, McNeill G. Assessing the relative validity of the Scottish Collaborative Group FFQ for measuring dietary intake in adults. Public Health Nutrition. 2017;20(3):449-55.

44. Collins GS, Reitsma JB, Altman DG, Moons KGM. Transparent reporting of a multivariable prediction model for individual prognosis or diagnosis (TRIPOD): the TRIPOD Statement. BMC Medicine. 2015;13(1):1.

For peer review only

For peer review only - http://bmjopen.bmj.com/site/about/guidelines.xhtml

WORD COUNT

4,966 words excluding words in figures/tables/boxes, contributorship statement, competing interests, funding, acknowledgements and references

For peer review only

For peer review only - http://bmjopen.bmj.com/site/about/guidelines.xhtml

FIGURE LEGENDS

Figure 1. Schematic illustrating the evolving approach to treatment of inflammatory bowel disease, with the aim of improving treatment outcomes through individualised precision-medicine. Current treatment selection is stratified and modified based on diagnosis, disease phenotype, imaging (radiological and endoscopic) and limited clinical biomarkers, such as blood and stool markers of inflammation, drug metabolising enzyme activity, drug levels, and anti-drug antibodies. Precision medicine approaches integrating additional complex multi-omic data with information about environmental factors such as dietary intake, smoking and physical activity levels (the exogenous 'exposome'), may enable individualised treatment selection through predictive modelling. Precision medicine may also help to identify of at-risk populations, predict disease course, reduce unnecessary patient risk and health service costs associated with ineffective pharmacological therapies, and guide non-pharmacological interventions such as dietary modification. Figure created with BioRender.com

Figure 1 abbreviations: c-reactive protein (CRP), thiopurine methyltransferase (TPMT)

Figure 2. Study overview schematic. 1,325 participants with IBD planned to commence an advanced therapy will be recruited, including a nested sub-cohort of 300 CD patients (CD-metaRESPONSE). All participants will collect two stool sample tubes at each study assessment timepoint (baseline, week 14 and week 54). CD-metaRESPONSE participants will be required to collect a third stool sample tube at baseline and week 14. If a participant attends hospital for a face-to-face appointment within the baseline and/or week 14 study assessment window, blood samples will be collected. If a participant attends hospital for a lower gastrointestinal endoscopy at any time during the study period (pre- or post- treatment), biopsy samples will be collected. Participants will complete several questionnaires at each

For peer review only - http://bmjopen.bmj.com/site/about/guidelines.xhtml

assessment time point. For CD-metaRESPONSE participants, additional detailed analyses will be undertaken of metabolic profiles (metabolome) in stool and matched blood plus in-depth dietary assessment (additional elements highlighted in blue boxes). Data generated will be used to perform predictive modelling. Any remaining participant samples will form a large biorepository for use in future research. Figure created with BioRender.com

Figure 2 abbreviations: anti-tumour necrosis factor alpha (anti-TNFα), anti-interleukin (anti-IL), Bristol stool form scale (BSFS), Crohn's disease (CD), formalin-fixed paraffin embedded (FFPE), inflammatory bowel disease (IBD), International Physical Activity Questionnaire (IPAQ), janus kinase inhibitor (JAKi), Joint Pain and Stiffness Score (JPSS), Patient Reported Outcome-2 (PRO-2), Patient Reported Outcomes Measurement Information System (PROMIS), sphingosine-1-phosphate receptor (S1PR), ulcerative colitis (UC).

Figure 3. Flowchart providing overview of study events.

Figure 3 abbreviations: Crohn's disease (CD), inflammatory bowel disease (IBD), Janus kinase inhibitor (JAKi), Mayo Clinic Score (MCS), multidisciplinary team (MDT), Patient Reported Outcome-2 (PRO-2), simple endoscopic score for Crohn's disease (SES-CD), sphingosine-1-phosphate receptor (S1PR), Research Electronic Data Capture (REDCap), ulcerative colitis (UC).

Supplementary Figure 1. Power at different sample sizes for detecting associations in the primary analysis between microbiome variables at baseline and response to treatment at 14 weeks or remission at week 52, using two different plausible effect sizes (d=0.66 and d=0.30). Vertical lines mark the sample size required to achieve 80% power for the four different scenarios.

For peer review only

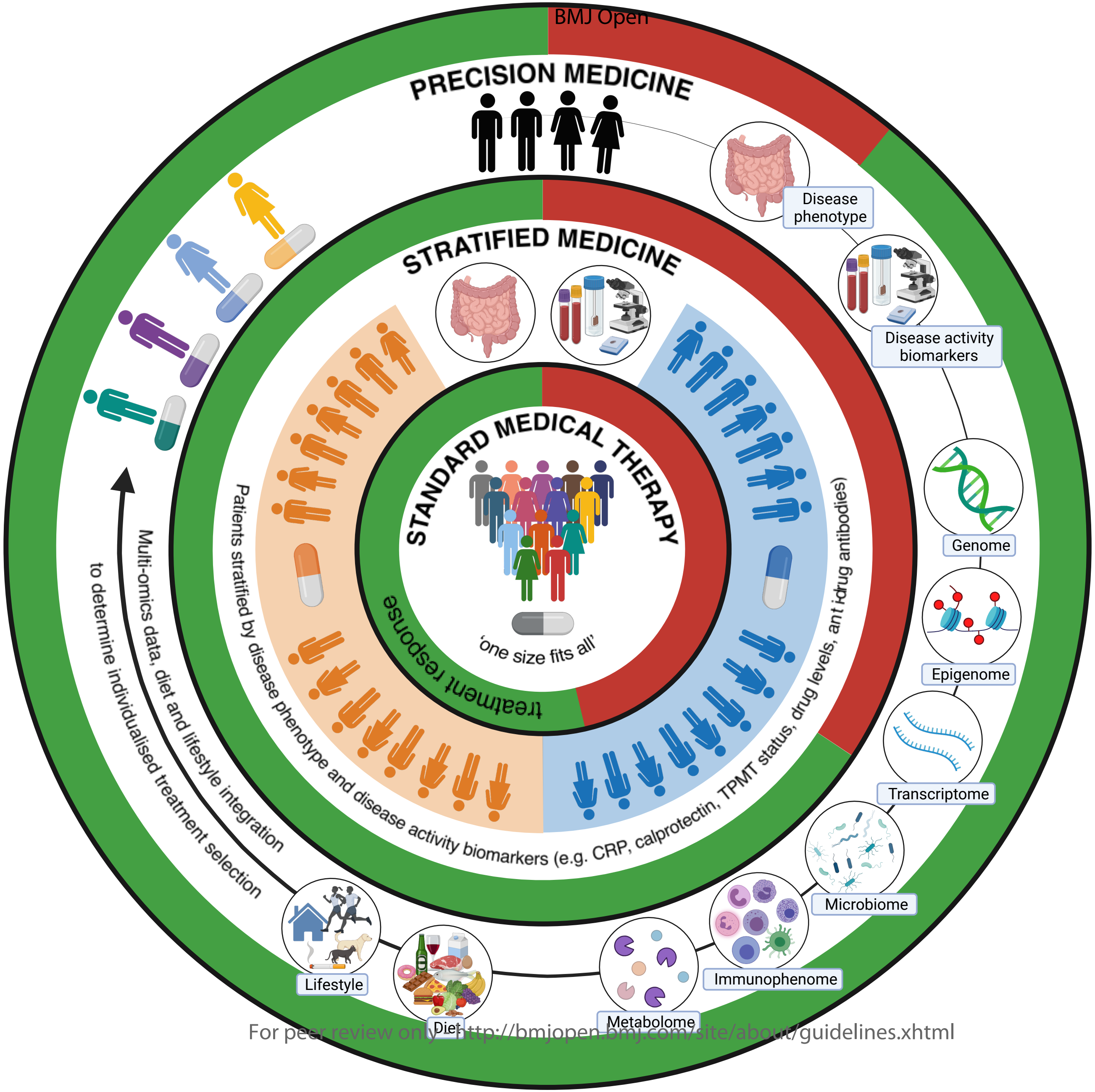

For peer review only - http://bmjopen.bmj.com/site/about/guidelines.xhtml

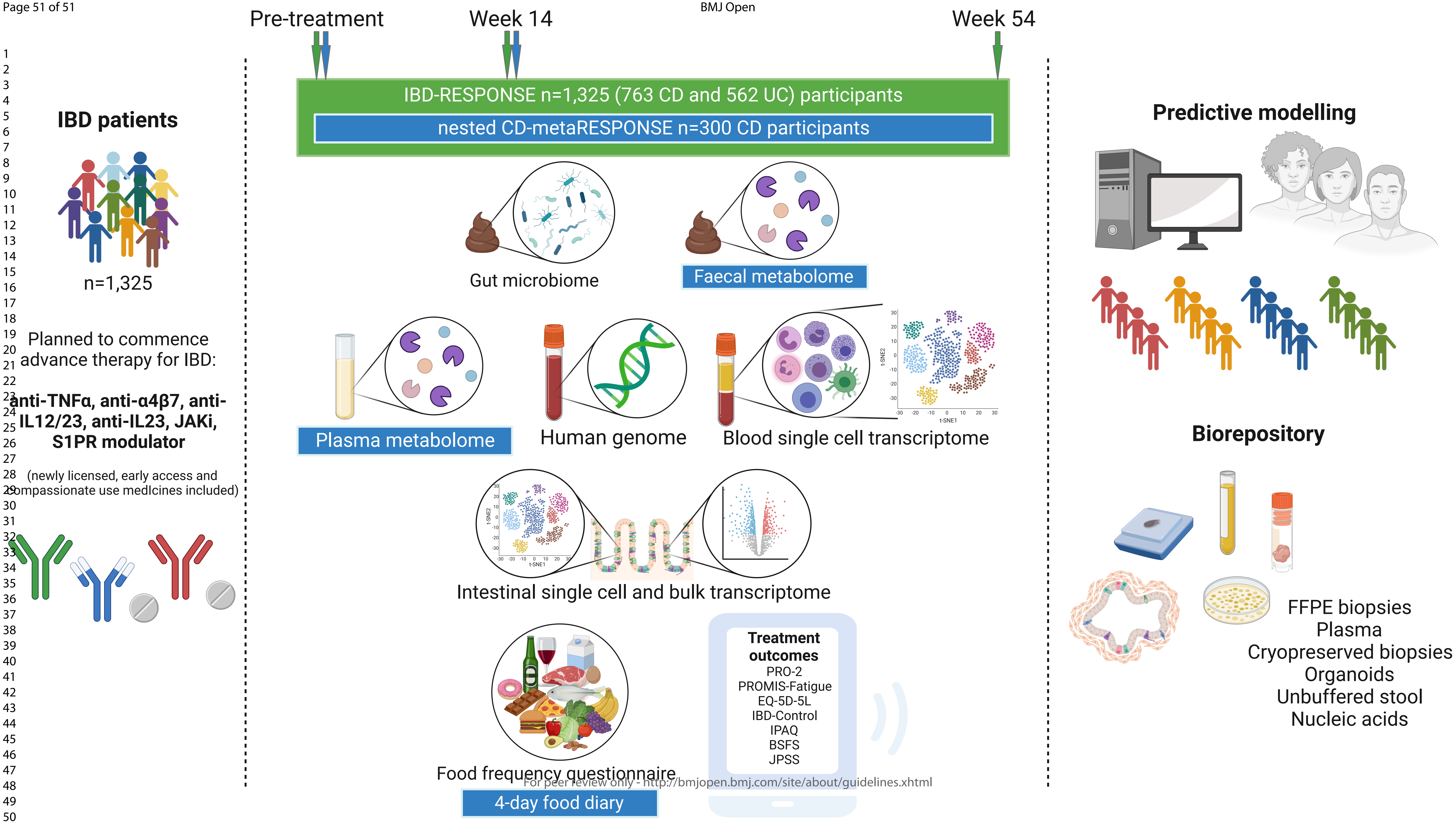

For peer review only - http://bmjopen.bmj.com/site/about/guidelines.xhtml

Potential participant identified

Where: Face-to-face clinics, telephone consultations, endoscopy lists, infusion suites, MDT meetings, virtual biologics clinics, IBD nurse helpline

Who: ≥16 years old; symptomatic, active luminal IBD; no minimum disease duration

Advanced therapy being commenced: Licensed biologic, JAKi or S1PR modulator (infliximab, adalimumab, vedolizumab, ustekinumab, tofacitinib, filgotinib, upadacitinib, ozanimod) or early access/compassionate use therapy (e.g. risankizumab)

Screening tips:

- UC symptoms: increased stool frequency relative to baseline AND at least one episode of recent rectal bleeding within e.g. last 3-5 days
- CD symptoms of active flare: presence of liquid or very soft stools AND/OR presence of abdominal pain
- Active disease confirmed by clinical test in 16 weeks prior to consent: faecal calprotectin, CRP, endoscopy or (CD only) imaging
- Participants do not need to be naïve to advanced therapies e.g. can previously have received a biologic
- Participants switching between advanced therapies do not need a minimum washout period
- Participants can be receiving or about to start concomitant thiopurine or methotrexate alongside planned advanced therapy
- Participants can be receiving or starting steroids at baseline provided likely to have fully weaned by week 14

Eligibility confirmed

Study information and informed consent given

- Participant Information Sheet (PIS) provided (paper or electronic format)
- Potential participant questions answered by local study team
- REDCap access given to participant to complete informed study consent

Consent verified

Stool sample collection kit given to/posted to participant once valid consent obtained and verified by local study team

Baseline assessment

Ideal: Week 0
Target: Up to 6 weeks prior to commencing advanced therapy
Permitted: Assessment >6 weeks prior to commencing advanced therapy is permitted, e.g. if delayed due to infusion unit capacity

All participants to complete after consent and before starting advanced therapy:

1. Participant questionnaires completed on REDCap
2. Stool sample tubes (n=2) collected and returned using prepaid Royal Mail Safebox™
3. If attending hospital for a face-to-face clinical encounter within assessment window, two tubes (total 20mls) blood collected

CD-metaRESPONSE sub-cohort (in addition to above requirements):

1. Third stool sample tube collected (provided in home stool sample collection kit)
2. Prospective 4-day food diary completed

Participants unable or unwilling to complete the PRO-2 questionnaire, not meeting the PRO-2 threshold for clinically active disease, missing stool samples or who do not start treatment with an advanced therapy will be withdrawn from the study

Eligibility re-confirmed

Baseline 4 day PRO-2 (minimum 2 days data) assessed to confirm inclusion criteria for clinically active disease met

Biologic, JAKi or S1PR modulator commenced

WEEK 0

Week 14 assessment

Ideal: Week 14
Target: Week 12 - 16
Permitted: Week 10 - 20

As per baseline assessment (including additional requirements for CD-metaRESPONSE)

Week 54 assessment

Ideal: Week 54
Permitted: Week 48 - 60

All participants:

1. Participant questionnaires completed on REDCap
2. Stool sample tubes (n=2) collected and returned using prepaid Royal Mail Safebox™

If a participant stops treatment, the next planned study assessment should be brought forward and completed as a treatment discontinuation assessment. Where an alternative advanced therapy is then commenced, this discontinuation assessment will serve as the baseline assessment for the next therapy. Further assessments should then be completed at week 14 and 54 after commencing a new advanced therapy as per the study protocol

In addition to the above assessments, if a participant attends hospital for a planned lower gastrointestinal endoscopy at any time during the study period following consent, the following samples/data will be collected: up to 12 research biopsies, patient-reported 1-day PRO-2 score, endoscopic assessment of disease activity data (SES-CD/MCS endoscopic subscore)

For peer review only - http://bmjopen.bmj.com/site/about/guidelines.xhtml

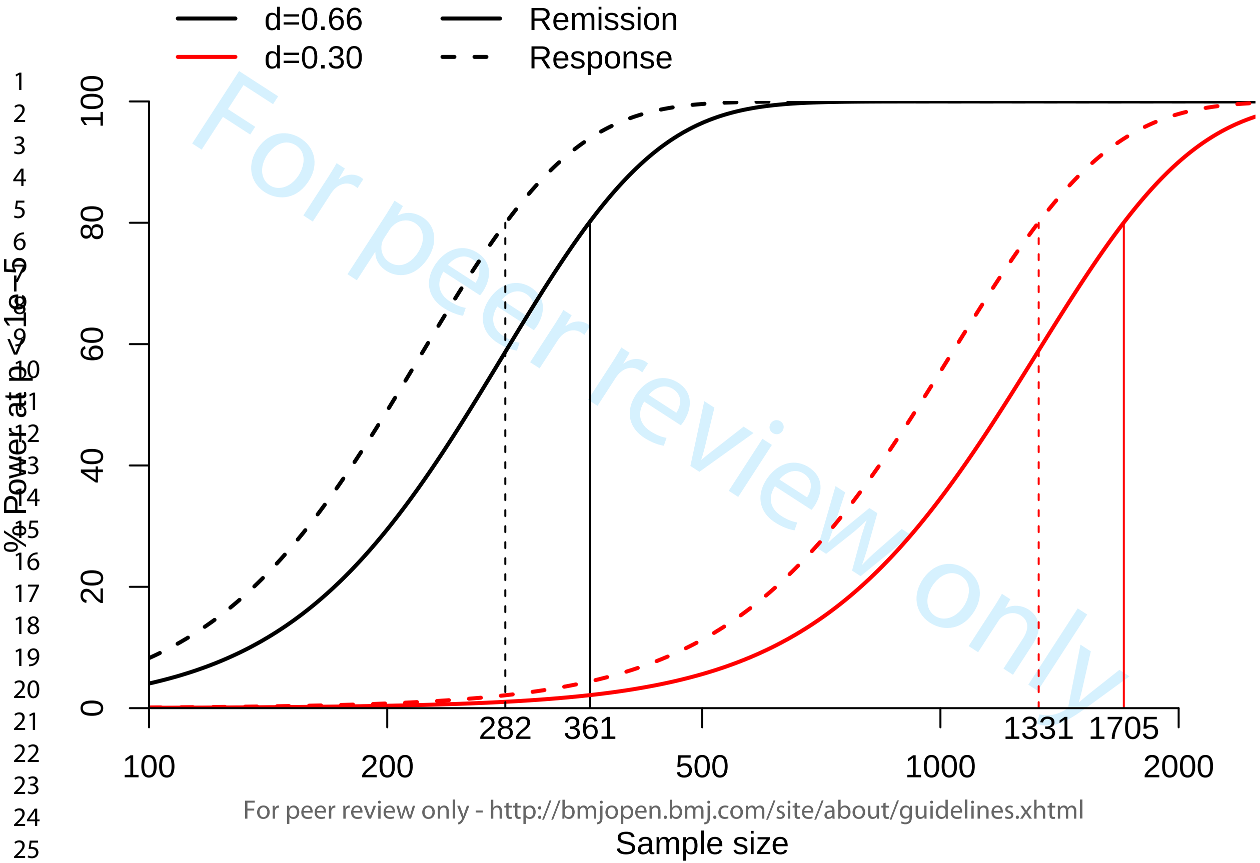

For peer review only

BMJ Open

Defining predictors of responsiveness to advanced therapies in Crohn's disease and ulcerative colitis: Protocol for the IBD-RESPONSE and nested CD-metaRESPONSE prospective, multicentre, observational cohort study in precision medicine

Journal:	*BMJ Open*
Manuscript ID	bmjopen-2023-073639.R2
Article Type:	Protocol
Date Submitted by the Author:	23-Nov-2023
Complete List of Authors:	Wyatt, Nicola; Newcastle University Translational and Clinical Research Institute, Faculty of Medical Sciences; Newcastle Upon Tyne Hospitals NHS Foundation Trust, Department of Gastroenterology<br>Watson, Hannah; Newcastle University Translational and Clinical Research Institute, Faculty of Medical Sciences<br>Anderson, Carl ; Wellcome Sanger Institute, Wellcome Genome Campus<br>Kennedy, Nicholas A; Royal Devon University Healthcare NHS Foundation Trust, Department of Gastroenterology; University of Exeter Inflammatory Bowel Disease and Pharmacogenetics Research Group<br>Raine, Tim; Cambridge University Hospitals NHS Foundation Trust<br>Ahmad, T.; Royal Devon University Healthcare NHS Foundation Trust, Department of Gastroenterology; University of Exeter Inflammatory Bowel Disease and Pharmacogenetics Research Group<br>Allerton, Dean; Newcastle University, Newcastle Clinical Trials Unit<br>Bardgett, Michelle; Newcastle University, Newcastle Clinical Trials Unit<br>Clark, Emma; Newcastle University, Newcastle Clinical Trials Unit<br>Clewes, Dawn; Newcastle University, Newcastle Clinical Trials Unit<br>Cotobal Martin, Cristina; Wellcome Sanger Institute, Wellcome Genome Campus<br>Doona, Mary; Newcastle Upon Tyne Hospitals NHS Foundation Trust, Department of Gastroenterology<br>Doyle, Jennifer; Newcastle University Translational and Clinical Research Institute, Faculty of Medical Sciences<br>Frith, Katheirne; Newcastle University, Newcastle Clinical Trials Unit<br>Hancock, Helen; Newcastle University, Newcastle Clinical Trials Unit<br>Hart, Ailsa L.; St Mark's Hospital and Academic Institute, Department of Gastroenterology; Imperial College London, Department of Surgery and Cancer<br>Hildreth, Victoria; Newcastle University<br>Irving, Peter; Guy's and St Thomas' NHS Foundation Trust, Department of Gastroenterology; King's College London, School of Immunology & Microbial Sciences<br>Iqbal, Sameena; Wellcome Sanger Institute, Wellcome Genome Campus<br>Kennedy, Ciara; Newcastle University, Newcastle Clinical Trials Unit<br>King, Andrew; Newcastle Upon Tyne Hospitals NHS Foundation Trust, Department of Gastroenterology<br>Lawrence, Sarah; Newcastle University, Newcastle Clinical Trials Unit

Lees, Charlie W.; The University of Edinburgh MRC Institute of Genetics and Molecular Medicine; Western General Hospital, Institute of Genetics & Molecular Medicine
Lees, Robert; Newcastle Upon Tyne Hospitals NHS Foundation Trust, Department of Gastroenterology
Letchford, Laura; Wellcome Sanger Institute, Wellcome Genome Campus
Liddle, Trevor; Newcastle Upon Tyne Hospitals NHS Foundation Trust
Lindsay, James ; Barts Health NHS Trust, Department of Gastroenterology; Blizard Institute of Cell and Molecular Science, Centre for Immunobiology
Maier, Rebecca; Newcastle University, Newcastle Clinical Trials Unit
Mansfield, John; Newcastle University Translational and Clinical Research Institute, Faculty of Medical Sciences; Newcastle Upon Tyne Hospitals NHS Foundation Trust, Department of Gastroenterology
Marchesi, Julian R.; St Mary's Hospital, Division of Digestive Diseases, Department of Metabolism, Digestion and Reproduction
McGregor, Naomi; Newcastle University, Newcastle Clinical Trials Unit
McIntyre, Rebecca ; Wellcome Sanger Institute, Wellcome Genome Campus
Ostermayer, Jasmin; Wellcome Sanger Institute, Wellcome Genome Campus
Osunnuyi, Tolulope; Wellcome Sanger Institute, Wellcome Genome Campus
Powell, Nick; St Mary's Hospital, Division of Digestive Diseases, Department of Metabolism, Digestion and Reproduction; Imperial College Healthcare NHS Trust, Division of Digestive Diseases, Department of Metabolism, Digestion and Reproduction
Prescott, Natalie; King's College London, Department of Medical and Molecular Genetics, Guy's Hospital
Satsangi, J; University of Oxford, Nuffield Department of Medicine
Sharma, Shriya; Newcastle University, Newcastle Clinical Trials Unit
Shrestha, Tara; Newcastle University, Newcastle Clinical Trials Unit
Speight, Ally; Newcastle University Translational and Clinical Research Institute; Newcastle Upon Tyne Hospitals NHS Foundation Trust, Department of Gastroenterology
Strickland, Michelle; Wellcome Sanger Institute, Wellcome Genome Campus
Wason, James; Newcastle University, Population Health Sciences Institute
Whelan, Kevin; King's College London, Department of Nutritional Sciences
Wood, Ruth; Newcastle University, Newcastle Clinical Trials Unit
Young, Gregory; Newcastle University Faculty of Medical Sciences, Translational and Clinical Research Institute
Zhang, Xinyue; Newcastle University, Population Health Sciences Institute
Parkes, M; Cambridge University Hospitals NHS Foundation Trust, Department of Gastroenterology
Stewart, Christopher; Newcastle University Translational and Clinical Research Institute, Faculty of Medical Sciences
Jostins-Dean, Luke; University of Oxford, Kennedy Institute of Rheumatology
Lamb, Christopher; Newcastle University Translational and Clinical Research Institute, Faculty of Medical Sciences; Newcastle Upon Tyne Hospitals NHS Foundation Trust, Department of Gastroenterology

**Primary Subject Heading**:	Gastroenterology and hepatology
Secondary Subject Heading:	Pharmacology and therapeutics, Patient-centred medicine, Immunology (including allergy), Genetics and genomics, Diagnostics

Keywords:	Inflammatory bowel disease < GASTROENTEROLOGY, Gastroenterology < INTERNAL MEDICINE, MICROBIOLOGY, Patient-Centered Care, Patient Reported Outcome Measures

SCHOLARONE™
Manuscripts

Defining predictors of responsiveness to advanced therapies in Crohn's disease and ulcerative colitis: Protocol for the IBD-RESPONSE and nested CD-metaRESPONSE prospective, multicentre, observational cohort study in precision medicine

Nicola J. Wyatt[1,2], Hannah Watson[1], Carl A. Anderson[3], Nicholas A. Kennedy[4,5], Tim Raine[6], Tariq Ahmad[4,5], Dean Allerton[7], Michelle Bardgett[7], Emma Clark[7], Dawn Clewes[7], Cristina Cotobal Martin[3], Mary Doona[2], Jennifer A. Doyle[1], Katherine Frith[7], Helen Hancock[7], Ailsa Hart[8,9], Victoria Hildreth[7], Peter M. Irving[10,11], Sameena Iqbal[3], Ciara Kennedy[7], Andrew King[2], Sarah Lawrence[7], Charlie W. Lees[12,13], Robert Lees[2], Laura Letchford[3], Trevor Liddle[14], James O. Lindsay[15,16], Rebecca H. Maier[7], John C. Mansfield[1,2], Julian R. Marchesi[17], Naomi McGregor[7], Rebecca E. McIntyre[3], Jasmin Ostermayer[3], Tolulope Osunnuyi[3], Nick Powell[17,18], Natalie J Prescott[19], Jack Satsangi[20], Shriya Sharma[7], Tara Shrestha[7], Ally Speight[1,2], Michelle Strickland[3], James M. S. Wason[21], Kevin Whelan[22], Ruth Wood[7], Gregory R. Young[1], Xinyue Zhang[21], Miles Parkes[6], Christopher J. Stewart[*1], Luke Jostins-Dean[*23], Christopher A. Lamb[*1,2]

[*]Denotes co-senior authorship

Correspondence:

Dr Christopher Lamb, Translational & Clinical Research Institute, Faculty of Medical Sciences, Newcastle University, Newcastle upon Tyne, NE2 4HH. United Kingdom

christopher.lamb@newcastle.ac.uk

For peer review only - http://bmjopen.bmj.com/site/about/guidelines.xhtml

Affiliations:

1. Translational & Clinical Research Institute, Faculty of Medical Sciences, Newcastle University, Newcastle upon Tyne, United Kingdom
2. Department of Gastroenterology, The Newcastle upon Tyne Hospitals NHS Foundation Trust, Newcastle upon Tyne, United Kingdom
3. Wellcome Sanger Institute, Wellcome Genome Campus, Hinxton, Cambridgeshire, United Kingdom
4. Department of Gastroenterology, Royal Devon University Healthcare NHS Foundation Trust, Exeter, United Kingdom
5. Exeter Inflammatory Bowel Disease and Pharmacogenetics Research Group, University of Exeter, Exeter, United Kingdom
6. Department of Gastroenterology, Cambridge University Hospitals NHS Foundation Trust, Cambridge, United Kingdom
7. Newcastle Clinical Trials Unit (NCTU), 1-4 Claremont Terrace, Newcastle University, Newcastle upon Tyne, NE2 4AE, United Kingdom
8. Department of Gastroenterology, St Marks Hospital and Academic Institute, Gastroenterology, London, United Kingdom
9. Department of Surgery and Cancer, Imperial College, London, United Kingdom
10. Department of Gastroenterology, Guy's & St Thomas' NHS Foundation Trust, London, United Kingdom
11. School of Immunology & Microbial Sciences, King's College London, London, United Kingdom

For peer review only

12. Institute of Genetics & Molecular Medicine, University of Edinburgh, Edinburgh, United Kingdom
13. Edinburgh IBD Unit, Western General Hospital, NHS Lothian, Edinburgh, United Kingdom
14. Research informatics team, Clinical Research, The Newcastle upon Tyne Hospitals NHS Foundation Trust, Newcastle upon Tyne. United Kingdom
15. Department of Gastroenterology, Barts Health NHS Trust, The Royal London Hospital, London, United Kingdom
16. Centre for Immunobiology, Blizard Institute, Barts and the London School of Medicine Queen Mary University of London, London, United Kingdom
17. Division of Digestive Diseases, Department of Metabolism, Digestion and Reproduction, St Mary's Hospital, Imperial College London, London, United Kingdom
18. Department of Gastroenterology, Imperial College Healthcare NHS Trust, London, United Kingdom
19. Department of Medical and Molecular Genetics, King's College London, Guy's Hospital, London, United Kingdom
20. Nuffield Department of Medicine, University of Oxford, Old Road Campus, Oxford, United Kingdom
21. Population Health Sciences Institute, Newcastle University, Newcastle upon Tyne, United Kingdom
22. Department of Nutritional Sciences, King's College London, United Kingdom
23. Kennedy Institute of Rheumatology, University of Oxford, Oxford, United Kingdom

For peer review only

Abstract

INTRODUCTION

Characterised by chronic inflammation of the gastrointestinal tract, inflammatory bowel disease (IBD) symptoms including diarrhoea, abdominal pain and fatigue can significantly impact patient quality of life. Therapeutic developments in the last 20 years have revolutionised treatment. Despite these developments, clinical trials and real-world data show primary non-response rates up to 40%. A significant challenge is an inability to predict which treatment will benefit individual patients.

Current understanding of IBD pathogenesis implicates complex interactions between host genetics and the gut microbiome. Most cohorts studying the gut microbiota to date have been underpowered, examined single treatments, and produced heterogeneous results. Lack of cross-treatment comparisons and well-powered independent replication cohorts hampers the ability to infer real-world utility of predictive signatures.

IBD-RESPONSE will use multi-omic data to create a predictive tool for treatment response. Future patient benefit may include development of biomarker-based treatment stratification or manipulation of intestinal microbial targets. IBD-RESPONSE and downstream studies have the potential to improve quality of life, reduce patient risk, and reduce expenditure on ineffective treatments.

METHODS AND ANALYSIS

This prospective, multicentre, observational study will identify and validate a predictive model for response to advanced IBD therapies, incorporating gut microbiome, metabolome, single-cell transcriptome, human genome and clinical data. 1,325 participants commencing

For peer review only

advanced therapies will be recruited from ~40 UK sites. Data will be collected at baseline, week 14 and week 54. The primary outcome is week 14 clinical response. Secondary outcomes include clinical remission, loss of response in week 14 responders, corticosteroid-free response/remission, time to treatment escalation, and change in patient-reported outcome measures.

ETHICS AND DISSEMINATION

Ethical approval was obtained from the Wales Research Ethics Committee 5 (ref:21/WA/0228). Recruitment is ongoing. Following study completion, results will be submitted for publication in peer-reviewed journals and presented at scientific meetings. Publications will be summarised at www.ibd-response.co.uk.

TRIAL REGISTRATION NUMBER

ISRCTN96296121(pre-results)

Keywords:

Inflammatory bowel disease, Crohn disease, gastrointestinal microbiome, multiomics, precision medicine

ORCiDs (alphabetical):

Tariq Ahmad: https://orcid.org/0000-0002-6058-5528

Dean Allerton: https://orcid.org/0000-0003-0507-4343

Carl A. Anderson: https://orcid.org/0000-0003-1719-7009

Michelle Bardgett: https://orcid.org/0000-0003-1393-2010

Emma Clark: https://orcid.org/0000-0003-0065-1463

Dawn Clewes: https://orcid.org/0000-0001-9600-859X

Cristina Cotobal-Martin: https://orcid.org/0000-0002-5877-2228

Mary Doona: https://orcid.org/0000-0001-9533-3745

For peer review only - http://bmjopen.bmj.com/site/about/guidelines.xhtml

Jennifer A. Doyle: https://orcid.org/0000-0001-7042-3269

Katherine Frith: n/a

Helen Hancock: https://orcid.org/0000-0002-1494-8551

Ailsa Hart: https://orcid.org/0000-0002-7141-6076

Victoria Hildreth: n/a

Peter M. Irving: https://orcid.org/0000-0003-0972-8148

Sameena Iqbal: https://orcid.org/0000-0003-0926-593X

Luke Jostins-Dean: https://orcid.org/0000-0002-2475-3969

Ciara Kennedy: https://orcid.org/0000-0003-2987-0977

Nicholas A. Kennedy: https://orcid.org/0000-0003-4368-1961

Andrew King: https://orcid.org/0000-0002-5002-4751

Christopher A. Lamb: https://orcid.org/0000-0002-7271-4956

Sarah Lawrence: n/a

Charlie W. Lees: https://orcid.org/0000-0002-0732-8215

Robert Lees: https://orcid.org/0009-0003-7439-1681

Laura Letchford: n/a

Trevor Liddle: n/a

James O. Lindsay: https://orcid.org/0000-0003-3353-9590

Rebecca H. Maier: https://orcid.org/0000-0002-7350-3288

John C. Mansfield: https://orcid.org/0000-0003-2490-7750

Julian R. Marchesi: https://orcid.org/0000-0002-7994-5239

Naomi McGregor: https://orcid.org/0000-0002-5961-124X

Rebecca E. McIntyre: https://orcid.org/0000-0001-5291-1533

Jasmin Ostermayer: https://orcid.org/0000-0001-6461-398X

Tolulope Osunnuyi: https://orcid.org/0000-0002-0482-917X

Miles Parkes: https://orcid.org/0000-0002-6467-0631

Nick Powell: https://orcid.org/0000-0003-3231-6950

Natalie J. Prescott: https://orcid.org/0000-0002-5901-7371

Tim Raine: https://orcid.org/0000-0002-5855-9873

Jack Satsangi: https://orcid.org/0000-0002-6357-9684

Shriya Sharma: https://orcid.org/0000-0001-5237-757X

For peer review only

Tara Shrestha: n/a

Michelle Strickland: https://orcid.org/0000-0001-8053-400X

Ally Speight: https://orcid.org/0000-0003-3184-9181

Christopher J. Stewart: https://orcid.org/0000-0002-6033-338X

James M. S. Wason: https://orcid.org/0000-0002-4691-126X

Hannah Watson: https://orcid.org/0000-0002-7965-938X

Kevin Whelan: https://orcid.org/0000-0001-5414-2950

Ruth Wood: https://orchid.org/0000-0002-8296-1774

Nicola J. Wyatt: https://orcid.org/0000-0001-5050-327X

Gregory R. Young: https://orcid.org/0000-0001-5342-1421

Xinyue Zhang: https://orcid.org/0000-0002-5321-4235

For peer review only

Article Summary

STRENGTHS AND LIMITATIONS OF THIS STUDY

- IBD-RESPONSE is the largest precision medicine study of its kind to date, designed to develop a predictive tool for treatment response – or failure to respond – to IBD treatment, promoting *a priori* selection of the right drug, for the right patient, at the right time.
- IBD-RESPONSE will establish one of the largest biorepositories of longitudinal stool, blood, matched tissue and organoids with detailed linked phenotypic, diet, genetic and treatment outcome data in patients with IBD.
- This study will use a standardised yet pragmatic methodology for sample collection, processing and storage, allowing multicentre participation, improving generalisation and transferability of findings to real-world clinical practice.
- Remote 'at home' patient data and stool collection will allow >40 IBD centres to contribute to recruitment, increasing power in this multi-omics study when compared to other studies in the field that generally recruit from one or a small number of centres.
- Whilst recruiting sites are limited to the UK only, identified cross-validation cohorts from the UK and Canada will increase generalisability of our findings.

For peer review only

For peer review only - http://bmjopen.bmj.com/site/about/guidelines.xhtml

INTRODUCTION

Crohn's disease (CD) and ulcerative colitis (UC) are the principal forms of inflammatory bowel disease (IBD).(1) Characterised by symptoms including diarrhoea, rectal bleeding, abdominal pain and extra-intestinal features such as fatigue, IBD can have a substantial negative impact on patient quality of life.(2) Approximately 20% of CD patients and 10% of UC patients are unable to work due to their condition.(3) The global prevalence of IBD is rising. In the United Kingdom (UK), 1 in 125 people are affected, with prevalence expected to reach 1 in 100 by 2028.(4, 5) Outside of Western Europe and North America, the incidence is rising rapidly in many regions including South America, Latin America, Asia and Africa.(6, 7)

The biologics era has revolutionised IBD treatment in the last twenty years. Patients and clinicians have more advanced therapies to choose from than ever before. Several biologic classes are now licensed in the UK, targeting tumour necrosis factor alpha (TNFα) (including infliximab and adalimumab), interleukin (IL)-12 and/or -23 cytokine pathways (ustekinumab, risankizumab and mirikizumab), or the gut-homing α4β7 integrin (vedolizumab). In addition, recently licensed small molecule therapies for UC include the Janus kinase inhibitors (JAKi) tofacitinib, filgotinib and upadacitinib, and the sphingosine-1-phosphate receptor (S1PR) modulator ozanimod. With several additional therapies in advanced stages of development or having completed phase 3 randomised clinical trials, the number of treatments available to patients is likely to increase.(8)

Current understanding of biological mechanisms driving the pathogenesis and natural history of IBD, implicates complex interactions between host genetics and the gut microbiome (bacteria, viruses, fungi, archaea and phage).(9) Whilst large clinical cohorts for human genetic discovery have led to major advances in understanding disease pathogenesis,(10)

For peer review only - http://bmjopen.bmj.com/site/about/guidelines.xhtml

cohorts for the study of gut microbiota have mostly been under-powered. Nonetheless, existing research intriguingly suggests utility of microbiome signatures in predicting response to therapy. In a small prospective study of 85 patients starting vedolizumab therapy, greater alpha-diversity and higher abundance of *Roseburia inulinivorans* and a Burkholderiales species at baseline were associated with therapeutic-induced remission in CD.(11) Incorporation of microbial taxonomy data alongside clinical data in a predictive model produced an area under the receiver operating characteristic curve (AUC) of 0.776 (compared to an AUC of 0.619 using clinical data only). A larger study of 232 patients receiving ustekinumab implicated *Bacteroides* and *Faecalibacterium* as predictors of treatment response.(12) Here, a predictive model of response to ustekinumab using clinical metadata produced an AUC of 0.616, rising to 0.844 when combined with baseline bacterial profile data.

Beyond IBD, further proof of concept that the gut microbiome is of prognostic importance in the context of systemically administered immune-targeted therapies is found with immune checkpoint inhibitor treatment in cancer, where experimental animal data demonstrated the beneficial impact of microbial modulation on treatment outcome.(13-15)

Metabolites derived from the gut microbiome are important intermediaries in the host-microbiome dialogue.(16) Specific classes of metabolites, such as bile acids (BA),short-chain fatty acids (SCFA) and tryptophan metabolites, may play a role in modulating disease activity and treatment responsiveness in IBD.(17) In a study of 185 (77 UC, 108 CD) patients commencing anti-cytokine (anti-TNFα or anti-IL12/23) or anti-integrin (vedolizumab) therapy, metabolomic and proteomic analysis of blood in addition to taxonomic and functional profiling of stool samples was conducted.(18) Among patients receiving anti-cytokine therapy, 120 enzymes were differentially abundant in baseline samples of remitters versus non-remitters. Single-species dominance (>50% of enzyme copies in >50% of samples explained by a single

For peer review only - http://bmjopen.bmj.com/site/about/guidelines.xhtml

species) was observed for 8/120 enzymes. *Eggerthella lenta* was dominant for 5 of these 8 enzymes, 3 of which are involved in secondary BA biosynthesis. Metabolomic analysis of baseline blood samples revealed significant enrichment of serum secondary BAs in patients achieving week 14 clinical remission. Paired baseline stool samples revealed a significant positive correlation with the abundance of 7α/β-dehydroxylation enzymes (responsible for primary to secondary BA conversion), presence of which was associated with a preferential response to anti-cytokine therapy. This was replicated by the authors in a small validation cohort of 46 patients initiating anti-TNFα (infliximab) therapy. In a small study of 29 patients with moderate-severe UC receiving vedolizumab treatment, untargeted metabolomic analysis of stool showed significantly higher levels of SCFAs including butyrate in those achieving week 14 remission (defined as total Mayo score ≤2, all subscores ≤1) versus non-remitters.(19) Combining metabolite data (SCFA levels for butyrate and isobutyric acid) with microbial profile data predicted anti-integrin response with an AUC of 0.961.

Whilst these previous studies are important first steps to utilising gut microbial signatures in stratified treatment algorithms, they were mostly underpowered, examined single treatments, used different sequencing technologies, and produced heterogeneous non-overlapping results. The lack of cross-treatment comparisons and well-powered independent replication cohorts hampers the ability to infer real-world utility of these predictive signatures, and to move from observations of association to causation in IBD.(20)

METHODS AND ANALYSIS

Study rationale

A significant challenge to effective, personalised use of biologic or small molecule therapies (collectively termed ‘advanced therapies’) in IBD is an inability to predict which class

For peer review only - http://bmjopen.bmj.com/site/about/guidelines.xhtml

of treatment is most likely to benefit an individual patient (see *Figure 1*). Despite increasing therapeutic options, clinical trial and real-world efficacy data show primary non-response rates of up to 40% across all therapeutic classes and in those with initial symptomatic benefit, up to 40% lose response by 1 year.(1, 21-23) Consequently, complications of chronic, active inflammation including strictures, fistulae and malignancy, continue to affect a substantial number of patients and have a negative impact on patient quality of life.(2) Up to 30% of CD patients require surgical intervention within 10 years of diagnosis, and around 15% of UC patients will ultimately require a colectomy.(24) Off-target side effects may also occur, including infection and malignancy.(1) With average treatment costs of £6,156/year for CD and £3,084/year for UC, future treatment algorithms must avoid the potential morbidity and additional cost associated with expensive treatments that do not benefit individual patients.(25)

The absolute importance of precision medicine research to identify biomarkers for treatment stratification and develop prognostic algorithms was highlighted by two recent national research prioritisation exercises incorporating responses from almost 3,000 patients, their families and friends.(1, 26) Validated prognostic models for treatment stratification do not exist and understanding of mechanisms controlling treatment non-response is limited. Through a multi-omic, precision medicine approach, the IBD-RESPONSE study seeks to improve selection of the right drug, for the right patient, at the right time. Other translational outputs of IBD-RESPONSE could bring into focus potential non-pharmacological approaches to treating IBD that do not necessarily involve large health economic expenditure. This could include manipulating the gut microbiome via the microbiota, through refinement of faecal microbial transplant protocols, use of pre- and probiotics, and dietary interventions.

For peer review only

Scientific objectives of IBD-RESPONSE

The primary scientific objective of IBD-RESPONSE is to identify and validate a predictive model for clinical response or failure to respond to advanced therapies in IBD after 14 weeks of therapy (the primary clinical outcome, see below). Modelling will incorporate gut microbiome, human genome, blood and intestinal single cell transcriptome data, and detailed clinical data. Through data derived from a nested sub-cohort (CD-metaRESPONSE), predictive modelling will also include detailed dietary information and blood and faecal metabolome data. The co-primary scientific objective of IBD-RESPONSE is to determine the relationship between clinical response and remission at week 14 and baseline gut microbiome.

Secondary scientific objectives of IBD-RESPONSE are to determine if there is a relationship between the microbiome at baseline or changes in the microbiome following advanced therapy with any of the secondary clinical outcomes (*Box 1*). Further exploratory scientific objectives of IBD-RESPONSE are listed in *Box 2.*

- Clinical remission at week 14.
- Clinical response at week 54.
- Clinical remission at week 54.
- Loss of response at week 54 in week 14 responders.
- Durable corticosteroid-free response or remission at week 54 defined as receiving no corticosteroids between week 14 and week 54 assessments inclusive and not meeting criteria for loss of response.
- Time to treatment escalation from baseline, defined as:
    - Advanced therapy switch due to lack of efficacy/those with loss of response (does not include biosimilar switch or switch from IV to SC route).

For peer review only

- Dose intensification of drug due to lack of efficacy (does not include intensification based on therapeutic drug monitoring without flare in responders).
- Resectional intestinal surgery (does not include examination under anaesthesia procedures in patients with perianal CD).
- Induction or dose escalation of corticosteroids.

- Time to treatment escalation as defined above, but disregarding dose intensification.
- Time to treatment escalation as defined above, among week 14 responders.
- Time to discontinuation of index drug (persistence).
- Incidence of and time to potential side effects of treatment during follow up.
- Continuation of drug at week 14 and/or week 54 in those not meeting criteria for response and/or remission.
- Change from baseline SF subscore at week 14 and/or 54 (both CD and UC).
- Change from baseline RB subscore at week 14 and/or 54 (UC only).
- Change from baseline AP subscore at week 14 and/or 54 (CD only).
- Development of anti-drug antibodies by week 14 or 54.
- Change in CRP from baseline at week 14 or 54 (50% reduction or absolute value ≤5mg/L deemed as clinically significant).
- Change in faecal calprotectin from baseline at week 14 or 54 (50% reduction or absolute value ≤100μg/g deemed as clinically significant).
- Endoscopic remission during follow up (Mayo endoscopic subscore ≤1 for UC or SES-CD ≤2 for CD).

For peer review only - http://bmjopen.bmj.com/site/about/guidelines.xhtml

- Change in quality of life, physical activity dietary intake, joint pain and fatigue as measured by study questionnaires.

Box 1. IBD-RESPONSE secondary clinical outcome measures

Box 1 abbreviations: abdominal pain (AP), Crohn's disease (CD), c-reactive protein (CRP), intravenous (IV), rectal bleeding (RB), subcutaneous (SC), stool frequency (SF), simple endoscopic score for Crohn's disease (SES-CD), ulcerative colitis (UC)

- Test the association of microbial metabolites (metabolome) in stool or plasma, human genetics and/or single cell transcriptome data from blood or intestinal tissue with the above primary and secondary objectives.
- Determine the influence of diet on the gut microbiome and treatment response in IBD, and the factors associated with dietary intake in IBD.
- Explore host human genetic-gut microbiome-metabolome interactions in IBD pathogenesis and causal pathways to treatment response.
- Ascertain the utility of archived endoscopy collected FFPE biopsies at predicting/imputing the gut microbiome and for inclusion in the predictive model.
- Establish a longitudinal tissue, organoid and stool biobank from this well characterised clinical cohort.

Box 2. IBD-RESPONSE exploratory scientific objectives

Box 2 abbreviations: inflammatory bowel disease (IBD), formalin fixed paraffin embedded (FFPE)

For peer review only

Study design

The design of IBD-RESPONSE and the nested CD-metaRESPONSE studies are summarised in *Figure 2*. This prospective, observational, multi-centre, cohort study will recruit participants with IBD (CD, UC, IBD-unclassified (IBD-U)) who are due to commence either biologic, JAKi or S1PR modulator therapy for symptomatic, clinically active (moderate to severe) luminal disease. Participants do not have to be naïve to advanced therapies and may be recruited when switching within or between class of advance therapy. Participants may be taking or planned to start concurrent thiopurines or methotrexate as combination therapy. Participation in the study will not change standard clinical care received. Detailed longitudinal clinical data will be collected alongside stool, blood and (where possible) biopsy samples, patient reported outcome measures, and dietary intake. Planned recruitment of 1,325 participants will consist of 762 patients with CD and 563 patients with UC (or IBD-U).

Data will be collected at baseline (prior to starting treatment), week 14 (following completion of induction therapy) and week 54. Participants will collect stool samples at each study timepoint. If a participant attends hospital within the baseline and/or week 14 study window, two blood samples (per timepoint) will be collected. Where a participant undergoes endoscopy as part of routine clinical care during the study period, up to 12 research biopsies will be collected.

Of 762 CD participants, 300 will be consented to take part in the nested CD-metaRESPONSE sub-cohort. Inclusion criteria are identical to the main cohort. Clinical data collection will occur at the same time points as in the main cohort. Additional study components will include completion of a 4-day food diary questionnaire, capturing all food and drink consumed at the baseline and week 14 timepoints. This is in addition to the food

For peer review only

frequency questionnaire completed by all participants. CD-metaRESPONSE participants will also be required to provide two additional stool tubes (one at baseline and one at week 14) for faecal metabolome analysis. Participants will be recruited to CD-metaRESPONSE from a limited number of the participating sites. These sites will initially preferentially recruit eligible participants with CD to CD-metaRESPONSE. Once the recruitment target of 300 participants is achieved, all new participants identified with a diagnosis of CD will be recruited to the main cohort.

All participants recruited to IBD-RESPONSE will be invited to co-recruit to IBD BioResource (if not already participating). IBD BioResource is a national platform and recallable biorepository linked to the National Institute for Health and Care Research (NIHR) BioResource that is designed to expedite IBD research, currently with >36,000 participants.(27) Co-recruiting participants to IBD-RESPONSE and the IBD BioResource platform, will generate a rich dataset and provide a long term means of archiving data from IBD-RESPONSE to facilitate ongoing research and maximise downstream patient benefit. To minimise participant burden, the contact for recruitment to IBD BioResource can occur any time in the 12 months following consent to IBD-RESPONSE. If a participant ultimately decides not to participate in the IBD BioResource they will not be withdrawn from IBD-RESPONSE.

If a participant discontinues treatment prior to week 14 or week 54 follow up, the next timepoint assessment will be brought forward and completed as a treatment discontinuation assessment. Where a participant does not respond to the first prescribed advanced therapy and a second (or third) advanced therapy is subsequently prescribed, this discontinuation assessment will act as the baseline sample and data collection assessment for the successive advanced therapy. Follow up samples and data collection will occur at week 14 and week 54 following commencement of each successive agent. The participant may remain in the study

For peer review only - http://bmjopen.bmj.com/site/about/guidelines.xhtml

For peer review only

up to 54 weeks after commencement of a particular agent or until the end of the study period. We anticipate that up to 40% of patients will not respond to the initial prescribed therapy or will lose response by 1 year and will move on to a second (or third) advanced therapy. We therefore anticipate that recruitment of 1,325 participants may capture approximately 1,760 new treatment initiation episodes.

Study setting

This multi-centre cohort study will be conducted at >40 study centres, based within National Health Service (NHS) Trusts across the UK. All sites must be able to accommodate the needs of the IBD-RESPONSE cohort including clinical engagement, research nurse support and facilities for assessments.

Eligibility criteria

Individuals will be deemed eligible to enrol in the study if they fulfil all inclusion criteria and meet none of the exclusion criteria (*Box 3*).

For peer review only - http://bmjopen.bmj.com/site/about/guidelines.xhtml

INCLUSION CRITERIA

Individuals must fulfil all the following criteria to be enrolled in the study.

For peer review only

For peer review only - http://bmjopen.bmj.com/site/about/guidelines.xhtml

- Aged 16 years and over.
- Diagnosis of IBD: CD, UC or IBD-U.*
- Already participating or willing to be approached for participation in IBD BioResource.
- Willing and able to provide informed consent.
- Willing to undertake study procedures including:
  - Completion of study questionnaires
  - Collection of home stool specimens
- Provision of blood and (where applicable) biopsy specimens.
- Symptoms of active luminal IBD (see definition of 'Clinically active disease', *Table 1*).
- At least one biochemical, endoscopic or radiological marker of active disease within 16 weeks of study consent (see *Box 4* and *Box 5*)
- Intention of clinical team to commence one of the following licensed advanced therapies for active luminal IBD within 6 weeks of consent:△
  - Infliximab
  - Adalimumab
  - Vedolizumab
  - Ustekinumab
  - Risankizumab (CD only)
  - Mirikizumab (UC only)
  - Tofacitinib (UC only)
  - Filgotinib (UC only)
  - Upadacitinib
  - Ozanimod (UC only)

*Individuals with IBD-U will be managed as per the UC relevant protocol.

△Participants may be advanced therapy-naïve or -exposed. Any new biologic or small molecule drug that becomes licensed for the treatment of IBD during the planned study period will be permitted to allow study inclusion. Drugs used through Early Access to Medicines Schemes, compassionate use or expanded access schemes for unlicensed therapies are also permitted. Patients starting immunosuppressant monotherapy with a thiopurine or methotrexate are not eligible to take part. However, use of these treatments as part of combination therapy with an advanced therapy is not an exclusion to enrolment.

For peer review only - http://bmjopen.bmj.com/site/about/guidelines.xhtml

EXCLUSION CRITERIA

Individuals meeting any of the following criteria will not be eligible to participate in the study.

- Receiving oral corticosteroids for any indication where the dose is unlikely to be weaned by week 14.^
- Planned bowel resection surgery within 14 weeks of commencing therapy.
- Advanced therapy being commenced as rescue for ASUC.
- Advanced therapy being commenced as part of a CTIMP.
- Presence of an ileal pouch anal anastomosis.
- Presence of a stoma.
- Perianal CD in the absence of active luminal inflammation.
- Antibiotics or short-term (≤4 weeks) use of probiotics within the preceding 2 weeks.†
- FMT within the preceding 12 weeks or planned FMT within 14 weeks of commencing advanced therapy for IBD.‡

^Examples may include long term steroids for IBD where weaning by 14 weeks may not be possible irrespective of response to advanced therapy, or concurrent diagnosis where long term steroids are used e.g. polymyalgia rheumatica.

†Use of long-term (>4 weeks), stable doses of probiotics does not exclude individual participation but should be noted in the eCRF.

‡Use of antibiotics or prior FMT outside the exclusion period is permitted. Antibiotic use in the preceding 1 year and ever having received FMT will be noted in the eCRF.

Box 3. Eligibility criteria for IBD-RESPONSE

Box 3 abbreviations: Acute severe ulcerative colitis (ASUC), Clinical Trial of an Investigational Medicinal Product (CTIMP), Crohn's disease (CD), electronic case report form (eCRF), faecal microbial transplantation (FMT), inflammatory bowel disease (IBD), inflammatory bowel disease-unclassified (IBD-U), ulcerative colitis (UC).

Participants must have at least one biochemical, endoscopic or radiological (CD only) marker of clinically active luminal disease within 16 weeks of study consent (see *Box 4* (CD) and *Box 5* (UC)). Endoscopic assessment of disease activity will be judged locally and may

For peer review only - http://bmjopen.bmj.com/site/about/guidelines.xhtml

be assessed prospectively or retrospectively. Whilst pregnancy may influence advance therapy selection, pregnancy is not an exclusion criterion.

CD patients must also have at least one of the following documented within 16 weeks prior to consent:

- Faecal calprotectin ≥250 µg/g.
- CRP ≥6 mg/L.
- Any endoscopic evidence of active CD, defined as ulceration (with at least one ulcer ≥5mm) judged locally from available clinical data (as an approximation equivalent to SES-CD of ≥4 for ileal disease or ≥6 for ileocolonic or colonic disease.
- Active inflammatory disease on imaging (MRI/CT/ultrasound) judged locally from available clinical data.

Box 4. IBD-RESPONSE and CD-metaRESPONSE additional inclusion criteria: CD

Box 4 abbreviations: Crohn's disease (CD), C-reactive protein (CRP), Computed tomography (CT), Magnetic resonance imaging (MRI), Simple Endoscopic Score for Crohn's Disease (SES-CD)

UC/IBD-U patients must also have at least one of the following documented within 16 weeks prior to consent:

- Faecal calprotectin ≥250 µg/g.
- CRP ≥6 mg/L.
- Any endoscopic evidence of at least moderately active ulcerative colitis (of any extent including proctitis), defined as features of MCS endoscopic sub-score ≥2 (marked erythema, lack of vascular pattern, friability, erosions, spontaneous bleeding or ulceration.

Box 5. IBD-RESPONSE additional inclusion criteria: UC and IBD-U

For peer review only

For peer review only - http://bmjopen.bmj.com/site/about/guidelines.xhtml

Box 5 abbreviations: C-reactive protein (CRP), inflammatory bowel disease-unclassified (IBD-U), Mayo Clinic Score (MCS), ulcerative colitis (UC)

Clinical outcome measures

Key definitions related to clinical outcome measures can be found in *Table 1*.

Clinically active disease	• CD: Unweighted PRO-2 (CD) of average daily SF subscore ≥4 and/or average daily AP subscore ≥2.<br>• UC: Total PRO-2 (UC) ≥3 with RB subscore ≥1.<br>PRO-2 data will be entered by patients over 4 days (minimum 2 days PRO-2 data is permissible for PRO-2 calculation).
Clinical remission	Patient remains on drug and meets the following criteria:<br>• CD: Unweighted PRO-2 (CD) average daily SF subscore ≤2.8 and average daily AP subscore ≤1 (and neither worse than inclusion scores at baseline).<br>• UC: PRO-2 (UC) SF subscore ≤1 with a decrease of ≥1 point(s) in SF subscore from baseline, plus RB subscore = 0.<br>**AND** an absence of any of the following at time of assessment:<br>• Resectional bowel surgery at any time after baseline until time of current assessment.<br>• Current use of oral corticosteroids/failure to wean oral corticosteroids prescribed at baseline.
Clinical response	Meeting criteria for clinical remission **OR**:<br>• CD: unweighted PRO-2 (CD) ≥30% reduction in average daily SF subscore and/or ≥30% decrease in average daily AP subscore (and neither worse than inclusion scores at baseline).

For peer review only

	• UC: total PRO-2 (UC) decrease ≥1 and ≥30% from baseline, and a decrease in RB subscore ≥1 or an absolute RB subscore of ≤1.<br>**AND** an absence of any of the following at time of assessment:<br>• Resectional bowel surgery at any time after baseline until time of current assessment.<br>• Current use of oral corticosteroids/failure to wean oral corticosteroids prescribed at baseline.
Week 14 non-response	• Not meeting clinical response criteria AND not having stopped drug for any reason other than lack of efficacy.
Week 54 loss of response	• Not meeting clinical response criteria at week 54 having met clinical response criteria at week 14 (AND not having stopped drug for any reason other than inefficacy between week 14 and 54 assessments).

Table 1. IBD-RESPONSE key clinical definitions

Table 1 abbreviations: abdominal pain (AP), Crohn's disease (CD), patient reported outcome (PRO)-2, rectal bleeding (RB), stool frequency (SF), ulcerative colitis (UC).

For peer review only

Primary clinical outcome measures

Clinical response at week 14 after commencing therapy.

Secondary clinical outcome measures

Secondary clinical outcome measures are listed in *Box 1*.

Sample size calculations

The sample size (n=1,325 cases overall, including n=300 cases for CD-metaRESPONSE sub-cohort) was chosen to ensure sufficient power to answer the co-primary scientific objective (to detect associations between microbiome measures and clinical response or remission) and key exploratory scientific objectives (to detect associations

For peer review only - http://bmjopen.bmj.com/site/about/guidelines.xhtml

between metabolites and clinical response or remission, and to detect associations between genetics and microbiome measures). Sample size calculations for predictive models require extensive assumptions about the number and effect size of associations and the correlation structure of the data. We noted predictive models built with microbiome and/or metabolite measures with high in-sample predictive accuracy (including AUC=0.78 with n=84 from Ananthakrishnan et al.(11), AUC=0.91 with n=76 from Ding et al.(28), and AUC=0.84 with n=232 from the CERTIFI study(12)), implying that n=300 individuals (from CD-metaRESPONSE) and n=1,325 individuals (from IBD-RESPONSE), should be sufficient for high predictive in-sample accuracy.

Sample size for analysis of primary objective

We took plausible effect sizes for the primary scientific objective analysis from the effect of *Bacteroides* levels in stool on ustekinumab response (d=0.66, from the CERTIFI study(12)) and the effect of anti-drug immunogenicity on remission after anti-TNFα treatment (d=0.30, from the PANTS study(21)). We calculated power for a simple two-sample t-test (using the R package pwr), assuming the expected non-response rate at 14 weeks (23.8%) and non-remission rate at 54 weeks (63.1%) from the PANTS study. We assumed a conservative Bonferroni-corrected significance threshold of 1e-5 (correcting for 5000 microbiome measures). We calculated the minimum sample size required to achieve 80% power for the two effect sizes and two outcomes (*Supplementary Figure 1*), showing that relatively low sample sizes are required to have high power to detect the larger plausible effect size (n=282 for remission and n=361 for response), but that larger sample sizes are required to have high power to detect the smaller plausible effect size (n=1,331 for remission and n=1,705 for response). The sample size of n=1,325 chosen for our study gives a power of close to 100% for the larger *Bacteroides* effect size for both week 14 response and week 54

For peer review only - http://bmjopen.bmj.com/site/about/guidelines.xhtml

remission, and 57% and 80% for the smaller anti-drug immunogenicity effect size for week 14 response and week 54 remission, respectively.

Sample size for analysis of secondary and exploratory scientific objectives

We took plausible effect sizes for the exploratory metabolite analysis from the effects of three selected stool lipid and bile acid metabolites on anti-TNFα response from Ding et al.(28): faecal triglyceride (d=1.00), and two bile acid metabolites; BA1 (d=0.89) and BA3 (d=0.70). We assumed a conservative Bonferroni-corrected significance threshold of 5e-5 (correcting for 1000 metabolites). For n=300, this gives a power of 99.7%, 91% and 74% for faecal triglyceride, BA1 and BA3 respectively for week 14 response, and 100%, 99.8% and 91% for week 54 remission.

We do not anticipate that this study on its own will be well powered to detect new associations between genotype and microbiome measures in IBD. It is known from studies of healthy individuals that genetic variants that explain more than 3% of variation (R^2=0.03) in microbial abundance are rare.(29) Assuming a conservative significance threshold of < 1e-11 (correcting for 1e6 independent genotypes and 5000 microbiome measures), n=1,325 samples would only have 33% power to detect associations with R^2=0.03 (calculated using genpwr(30)). We will therefore combine our samples with a further genotype/microbiome study of IBD (PREdiCCt) to increase sample size to n=2,325. This will provide us 80% power to detect genetic associations with R^2>0.025. In the case where such genotype/microbiome measure associations exist, and are associated with a causal biomarker for week 54 remission, a Mendelian Randomization analysis would have >80% power to demonstrate causality of this biomarker when the causal effect odds ratios is larger than 2 (calculated using mRnd(31)).

For peer review only - http://bmjopen.bmj.com/site/about/guidelines.xhtml

Study procedures and measures

Participants ≥16 years old may be identified from a variety of settings such as outpatient clinics (face-to-face or virtual), flare assessments, IBD clinical nurse specialist helplines/email contact, endoscopy examinations, infusion suites, multidisciplinary team meetings and virtual biologics clinics (*Figure 2*). Consent will be taken electronically using a REDCap (Research Electronic Data Capture) online database. Full informed e-consent will be supported by an appropriately delegated member of the study team, using a laptop, tablet or mobile device in the patient's own home, or using a hospital tablet, laptop, computer or patient mobile device during a scheduled visit to hospital as part of routine clinical care. Paper copies of the consent form will be made available for those patients unable to access e-consent. If a later decision is made not to commence an advanced therapy for IBD, the participant will not be eligible to continue in the study and must be withdrawn. Data collected up to the point of withdrawal may be used for the study. Any samples collected will be used for research within IBD-RESPONSE or stored for future research.

All research activity will be completed by the participant either remotely or during hospital visits scheduled to deliver routine clinical care (see *Figure 3*). Participants will be asked to complete data collection after consent and before starting treatment (baseline), and at week 14 and week 54 following commencement of advanced therapy in line with routine dosing schedule visits. This will include patient questionnaires, stool samples and, where applicable, blood and biopsy specimens. Participants will be asked to complete questionnaires with data entered directly into the study specific REDCap database. Paper questionnaires will be made available for those participants without access or who express a preference to complete in paper format. Participants will be asked to complete questionnaires related to

For peer review only

For peer review only - http://bmjopen.bmj.com/site/about/guidelines.xhtml

disease activity, health, physical activity, quality of life and diet at the three assessment timepoints (see *Box 6*).

Participant samples

All sample collection and processing will be standardised, with full requirements detailed in a study Sample Collection Manual.

Stool samples will be collected by participants at home using stool collection kits and returned using a prepaid Royal Mail Safebox™. All participants will collect two stool samples per study assessment timepoint (DNA Genotek OMNIgene®•GUT tube and universal polystyrene tube). Participants recruited to the CD-metaRESPONSE sub-cohort will be required to collect a third stool sample at baseline and week 14 (DNA Genotek OMNImet®•GUT tube). DNA extraction and metagenomic shotgun sequencing will be performed on buffered samples. Calprotectin will be measured in unbuffered stool. Remaining fresh stool and nucleic acids will be cryopreserved for use in future research.

Participants who attend a clinical appointment prior to commencing advanced therapy or within the week 14 visit window (week 10-20; week 12-16 preferred) will be asked to provide two blood samples (lithium heparin (LiH) tube and ethylenediaminetetra-acetic acid (EDTA) tube). Blood samples will be utilised for single cell analysis, plasma extraction and cryopreservation. Any remaining blood samples or derivatives will be stored at Newcastle Biobank for use in future research. Participants will not be asked to attend hospital specifically for blood sample collection. If participants are not scheduled to attend hospital face-to-face within either study assessment window, blood samples will not be taken.

If a participant has a lower gastrointestinal endoscopy as part of planned care during study participation, up to 12 research biopsies (one set of six biopsies to be collected from the

For peer review only - http://bmjopen.bmj.com/site/about/guidelines.xhtml

colon in all participants and a further set of six biopsies from the ileum in CD participants) will be taken. Where a participant meets the study eligibility criteria and a disease assessment endoscopy is planned prior to starting therapy, consent should be received ahead of their planned endoscopy to enable the collection of research biopsies. Biopsy samples will be utilised for single cell sequencing and organoid generation. 16S rRNA gene sequencing of both FFPE and fresh tissue will also be undertaken. Any remaining biopsy samples will be stored for use in future research.

COMPLETED BY ALL PARTICIPANTS AT BASELINE, WEEK 14 AND WEEK 54
**PRO-2 (CD or UC version depending on diagnosis)**<br>PRO-2 is a validated questionnaire measuring patient reported outcomes including stool frequency, abdominal pain and rectal bleeding.(32, 33)<br>**Bristol Stool Form Scale (BSFS)**<br>The BSFS is a 7-point scale that helps describe stool shape and consistency and assess bowel patterns and habits.(34)<br>**IBD-Control**<br>The IBD-Control questionnaire comprises 13 items plus a visual analogue scale (VAS) ranging from 0–100.(35) The questionnaire measures patient related outcome of their disease state during the past 2 weeks.<br>**PROMIS-Fatigue 8a Short Form**<br>The Patient Reported Outcomes Measurement Information System (PROMIS), are validated questionnaires that help evaluate patients' quality of life.(36)<br>**EQ-5D-5L**<br>The EQ-5D-5L is a quality-of-life questionnaire and is a widely used generic PROM incorporating five domains: 1) mobility, 2) self-care, 3) usual activities, 4) pain/discomfort,

For peer review only - http://bmjopen.bmj.com/site/about/guidelines.xhtml

5) anxiety/depression.(37, 38) Scores for each domain are combined to describe the patient's state of health.

International Physical Activity Questionnaire (IPAQ)

IPAQ is a commonly used self-reported questionnaire to estimate physical activity and sedentary behaviours for adults across a range of socio-economic settings.(39) The IPAQ measures the type of physical activities people do as part of their everyday lives.(39)

IBD-RESPONSE Joint Pain and Stiffness Score (JPSS)

The Ankylosing Spondylitis Disease Activity Score (ASDAS) is a patient reported questionnaire which quantifies clinical disease activity in Ankylosing Spondylitis (AS) and combines five disease activity variables (four 10-point Likert scale patient symptom responses and a CRP measurement), to produce a single score.(40-42) We believe joint pain to be an under-recognised symptom in active inflammatory bowel disease which may change in response to therapy as inflammation resolves/fails to resolve and so we have modified the ASDAS to assess joint pain, swelling and stiffness in all IBD-RESPONSE patients irrespective of whether they have a rheumatological diagnosis. We have called this modified score the IBD-RESPONSE Joint Pain and Stiffness Score (JPSS).

Food frequency questionnaire (FFQ)

The FFQ will be completed by participants directly into the study specific REDCap database and can also be completed on paper. The FFQ requests information on 175 food items, their typical portion size and frequency of consumption and has been extensively validated for measuring nutrient intakes in adults.(43) Data are converted to nutrient intake using the Composition of Foods Integrated Dataset (COFIDs), as well as diet quality indices and other food components (e.g. polyphenols) and food categorisation (e.g. ultra-processed foods). The FFQ data will initially be analysed at the Centre for Healthcare Randomised Trials (CHaRT), University of Aberdeen and other collaborating institutions at the Department of Nutritional Sciences, King's College London.

For peer review only - http://bmjopen.bmj.com/site/about/guidelines.xhtml

COMPLETED BY CD-METARESPONSE PARTICIPANTS AT BASELINE AND WEEK 14 ONLY
**4-day food diaries**<br>The 4-day food diary measures current food intake. It will comprehensively and prospectively measure all intake allowing calculation of energy and nutrient intake, dietary indices (e.g. diet diversity and Mediterranean diet), intake of ultra-processed foods, prebiotic and emulsifier intake.
Box 6. Summary of patient questionnaires completed throughout study period

For peer review only

Statistical analysis

The analysis approach for our primary objective will be to test for the association between features of the patient microbiome at baseline and primary clinical response to treatment at 14 weeks. Features will include alpha diversity, abundance of bacterial taxa (including species, genus and phyla) as well as the abundance of genes within various microbial pathways (for example using KEGG pathways, MetaCyc metabolic pathways and gene families). Association testing will be carried out using negative binomial regression, controlling for total sequence depth and pre-defined technical and clinical confounders, and significance will be determined using Benjamini-Hochberg multiple testing correction to ensure a false discovery rate of less than 5%.

For the predictive modelling aim, we will use a random forest classifier to predict primary response to treatment at 14 weeks using microbiome, host genetic and clinical features at baseline. Model parameters will be tuned and accuracy assessed using nested cross-validation. This full model will be compared to a clinical-variables-only model, with model performance quantified by area under the receiver operator curve (AUROC), as well as the

sensitivity, specificity and positive and negative predictive power. A further model will be fitted using the same approach including metabolomic and dietary data on the CD-metaRESPONSE subset. Our primary predictive measure will be reported for a random forest classifier, but a further sensitivity analysis will be carried out by fitting alternative prediction models to test whether this has a strong effect on the predictive accuracy, using both simpler models (including logistic regression with a LASSO penalty) and other more advanced methods (such as neural networks and support vector machines), The results of the predictive models will be reported in future publications according to the TRIPOD guidelines.(44)

Secondary and exploratory objective analyses will use the same general analysis approach as described above. For time-dependent events, such as treatment escalation due to loss of response, a Cox proportional hazards regression will be used to assess the impact of microbiome features on time to event, with patients censored at 54 weeks, last recorded (if lost to follow-up) or date of withdrawal (if withdrawn). The dietary data will be analysed to test the association between primary response and measures of specific nutrients (such as dietary fibre) and dietary indices will be used to assess adherence to certain recommended diets (such as achievement of food-based dietary guidelines or a “Mediterranean diet”). Host genotype data will be used to test for associations between primary response and generate polygenic risk scores of susceptibility to Crohn’s disease and ulcerative colitis, as well as pre-specified variants associated with response to therapy (including HLA-DQA1*05). In all these individual analyses, Benjamini-Hochberg will be applied used to control the false discovery rate at 5%.

Loss to follow-up and missing data will be handled during the analyses in different ways depending on the specific question being addressed. For the primary analysis at 14 weeks, individuals who are lost to follow-up or withdraw from the study before 14 weeks will

For peer review only

be removed from the analysis, though we will also carry out a robustness analysis where we include individuals lost to follow-up as non-responders to ensure the results are robust to this choice. Secondary and exploratory analyses at specific time-points will be treated in the same fashion. For time-dependent events, analysed using survival models, individuals who are lost to follow-up or withdraw will be treated as censored at this time point (the point of withdrawal for withdrawn participants, and the last point of contact for patients lost to follow-up), though we will also carry out sensitivity analyses where these are instead treated as adverse outcomes where appropriate (e.g. treating withdrawals as adverse events). For missing data, standard quality control criteria for microbiome, metabolome and genetic data will be used to remove variables with excessive missing data (as well as other markers of poor data quality). Where data is missing for microbiome or other experimental assays for specific individuals after QC, only participants with non-missing data for this variable will be analysed. When constructing and validating predictive models, individuals with missing data for the predictive variables being tested, or that have withdrawn or been lost to follow-up before the assessment time, will be excluded from model building and testing. Statistical analysis will be carried out in R.

Replication

Scaling up microbiome discoveries and providing validation of results is needed to benefit patients. Whilst IBD-RESPONSE is the largest project of its kind to date, it is a UK-only programme. Translating findings to maximise patient benefit requires international collaboration. Standardised data collection and data integration from different countries and ethnic groups is key to establishing predictors of treatment response that are generalisable and replicable.

For peer review only

We will validate our predictive model using an appropriate, already assembled microbiome validation cohort. To generate this replication cohort, we will use banked stool DNA from the Prognostic effect of Environmental factors in Crohn's and Colitis Study (PREdiCCt; www.predicct.co.uk), led by Professor Charlie Lees. PREdiCCt is a prospective observational study of IBD participants in clinical remission, designed to identify whether baseline factors (including genetics, dietary habits and gut microbiota) predict subsequent disease flare. We will perform metagenomic sequencing of 1,000 stool samples from patients who experienced a disease flare during the PREdiCCt study. We expect approximately 40% of PREdiCCt patients to experience a disease flare requiring commencement of biologics. As these patients all have baseline (clinical remission) stool microbiome samples in storage, they provide a well matched and cost-efficient set of samples for replicating IBD-RESPONSE results.

Potential future benefit to patients

IBD-RESPONSE will provide timely and important information regarding associations between the gut microbiome and responsiveness to treatment in IBD. It will likely highlight potential mechanisms through which the microbiota may drive inflammation. We hope that findings from IBD-RESPONSE will lead to new personalised avenues for IBD treatment through discovery and validation of predictive tools that may be incorporated directly into clinical practice or further tested in stratified clinical trials. This could lead to the development of experimental techniques to modify gut microbes; for example, donor selection for faecal microbial transplantation, identification of single or multiple strains of microbes, or use of antimicrobials, phage or microbial metabolites that may be used to induce a more 'treatment responsive' microbiome.

For peer review only - http://bmjopen.bmj.com/site/about/guidelines.xhtml

Patient and public involvement statement

IBD-RESPONSE was informed by two national patient research prioritisation exercises in IBD care led by members of our team and involving feedback from 3,000 people living with IBD, their family and friends.(1, 26) These identify aspects of precision medicine, microbiome and diet in IBD as of high importance. The initial IBD-RESPONSE grant proposal to the Medical Research Council (MRC) was reviewed by the NIHR Research Design Service North East and North Cumbria Patient and Public Involvement Panel and was presented and discussed at the 2020 Crohn's & Colitis UK Patient and Public Involvement in Research Day. IBD-RESPONSE has been supported by two patient representatives in the Study Oversight Committee (SOC) since inception. The study team have engaged with lay members of the SOC to ensure all patient facing documents including the participant information sheet (PIS), consent forms, stool collection guidance for patients and study questionnaires have undergone review. Patients will also be involved in dissemination activities relating to outputs from this research.

ETHICS AND DISSEMINATION

Ethical approval for the study was obtained from the Wales Research Ethics Committee 5 (reference 21/WA/0228). Recruitment to IBD-RESPONSE began in February 2022 and is currently ongoing at sites around the UK.

In line with the Newcastle University and The Newcastle upon Tyne Hospitals NHS Foundation Trust research data policy, datasets will be kept for at least five years after the date they were last accessed. Metadata linked to genomic and metagenomic datasets will include anonymised clinical information. Examples include diagnosis (UC/CD/IBD-U), disease

For peer review only

location, disease behaviour, complications, extra-intestinal manifestations, co-morbidities, family history, smoking history, surgical interventions, and outcomes from prior drug therapies.

Raw data files in the original format (e.g. fastq) and the accompanying anonymised phenotypic data will be uploaded to a public repository e.g. the NCBI database of Genotypes and Phenotypes (dbGaP) at https://www.ncbi.nlm.nih.gov/gap/.

As part of CD-metaRESPONSE, microbial sequence and faecal/serum metabolomic data will be integrated with single-cell RNA sequencing, human genomics and clinical outcome data; the whole dataset will be made available to other investigators and will be archived long term within the IBD BioResource to facilitate downstream research.

The CI, SMG, Sponsor, Funders and research team members are committed to ensure that the research findings are shared. Findings will be written up and submitted to a peer-reviewed scientific journal. Findings will be presented by the study team at national and international conferences for example the British Society of Gastroenterology (BSG) annual meeting, the European Crohn's and Colitis (ECCO) meeting and Digestive Diseases Week (DDW). The study team will prepare a lay summary of the study findings for dissemination to the study participants and members of the national patient group, Crohn's & Colitis UK. Following study completion, results will be submitted for publication in peer-reviewed journals and presented at national and international scientific meetings.

REGISTRATION DETAILS

Trial registration number: ISRCTN96296121 (pre-results).

FUNDING

The IBD-RESPONSE cohort is supported by a grant from the Medical Research Council (funder reference MR/T032162/1) and the CD-metaRESPONSE cohort by The Leona M. and

For peer review only - http://bmjopen.bmj.com/site/about/guidelines.xhtml

Harry B. Helmsley Charitable Trust (funder reference 2002-04255). Single cell RNA sequencing in IBD-RESPONSE and CD-metaRESPONSE is supported by a grant from the Helmsley Charitable Trust (funder reference 2304-05972). CJS is supported by a Sir Henry Dale Fellowship jointly funded by the Wellcome Trust and the Royal Society (Grant Number 221745/Z/20/Z) and the 2021 Lister Institute Prize Fellow Award. NJW is supported by the NIHR Academic Clinical Fellowship (ACF) programme. LJD is supported by a Sir Henry Dale Fellowship jointly funded by the Wellcome Trust and the Royal Society (Grant Number 208750/Z/17/Z) and the Kennedy Trust for Rheumatology Research. JMSW is funded by a NIHR Research Professorship (NIHR301614). XZ is funded by a NIHR Pre-doctoral Fellowship (NIHR302014).

ACKNOWLEDGEMENTS

We are grateful for support from the Newcastle Clinical Trials Unit, the National Phenome Centre, Imperial College London and the NIHR Biomedical Research Centres from Newcastle, Imperial and Cambridge. The views expressed are those of the authors and not necessarily those of our funders, the NIHR or the Department of Health and Social Care.

CONTRIBUTORSHIP STATEMENT

All authors contributed to clinical protocol and/or laboratory standard operating procedure design, development and operationalisation. CAL is the Chief Investigator of the programme. The grants to fund IBD-RESPONSE and CD-metaRESPONSE were conceptualised and written by CAL, LJ-D, CJS, MP, CAA, NAK, TR, TA, AH, HH, CWL, JCM, JRM, NP, NJP, AS and JS. Clinical study design, operationalisation, data acquisition and analytical plans were further developed by these authors plus DA, MB, EC, DC, MD, KF, VH, AK, CK, PMI, JOL, RL, SL, TL, NM, RHM, SS, TS, HW, JMSW, KW, NJW, RW, GRY and XZ with critical review

For peer review only

from all authors. Laboratory standard operating procedures were developed by CAL, CJS, HW, REM, LJ-D, CK, NAK, CA, MB, EC, DC, JAD, MD, SI, JOL, LL, CCM, JRM, JO, TO, MP, MS, NP, NJP, TR, MS, SS and NJW. Manuscript drafting was led by NJW and CAL with subsequent critical review and revision by all authors.

COMPETING INTERESTS

Tariq Ahmad reports personal grants from F. Hoffmann-La Roche AG, Biogen Inc, AbbVie, Janssen, Celltrion, Galapagos NV, Immunodiagnostik, and Takeda, outside the submitted work; personal fees for educational development/delivery from Pfizer, payment or honoraria for lectures, presentations, speakers bureaus, manuscript writing or educational events from Pfizer, Takeda, and F. Hoffman-La Roche AG; support for attending meetings from Celltrion, Tillotts, and Pfizer. Carl A. Anderson reports grants from the Wellcome Sanger Institute Quinquennial Review 2021-2026, Crohn's and Colitis Foundation (USA), the Medical Research Council, Open Targets UK, and the Helmsley Charitable Trust; consulting fees from BridgeBio Ltd, Genomics PLC, and Brigham & Women's Hospital Boston; payment or honoraria for lectures, presentations, speakers bureaus, manuscript writing or educational events from GlaxoSmithKline; support for attending meetings and/or travel membership from the Wellcome Sanger Institute Quinquennial Review 2021-2026; (Chair) of the Board of Trustees for the Sanger Prize; other interests as Director of Anderson Genomics Consultancy Ltd. Michelle Bardgett reports partial personal salary funding from the Medical Research Council. Ailsa Hart reports personal consulting fees from AbbVie, BMS, Celltrion, Falk, Galapagos, Janssen, Pfizer, Takeda, and Roche; payment or honoraria for lectures, presentations, speakers bureaus, manuscript writing or educational events from BMS, Celltrion, Falk, Galapagos, Janssen, Pfizer, Takeda, Roche, and AbbVie; support for attending meetings and/or travel from BMS, Celltrion, Falk, Galapagos, Janssen, Pfizer, Takeda, Roche,

For peer review only - http://bmjopen.bmj.com/site/about/guidelines.xhtml

and AbbVie. Peter M. Irving reports reports personal grants from Celltrion, Galapagos, and Pfizer, outside the submitted work; personal consulting fees from AbbVie, Takeda, BMS, Janssen, Arena, Pfizer, Galapagos, Lilly, Boehringer-Ingelheim, and Celgene; payment or honoraria for lectures, presentations, speakers bureaus, manuscript writing or educational events from AbbVie, Takeda, Janssen, Lilly, BMS, Pfizer, and Galapagos; support for attending meetings and/or travel from AbbVie and Tillotts. Luke Jostins-Dean reports grants from the Wellcome Trust, the Royal Society, the Kennedy Trust for Rheumatology Research, the Helmsley Charitable Trust, and the Medical Research Council; grants from Novartis Pharmaceutical, outside the submitted work; consulting fees from Nightingale Health and Genomics PLC. Ciara Kennedy reports partial salary funding from the Medical Research Council. Nicholas A. Kennedy reports grants from AbbVie, Biogen, Celltrion, Galapagos, and Immunodiagnostik; consulting fees from AbbVie, Bristol-Meyer Squibb, and Dr Falk; payment or honoraria for lectures, presentations, speakers bureaus, manuscript writing or educational events from AbbVie, Dr Falk, Tillotts, Galapagos, and Takeda; support for attending meetings and/or travel from Tillotts; participation (Chair) on the Board of the British Society of Gastroenterology IBD Clinical Research Group. Christopher A. Lamb reports grants from and/or consultancy for Janssen, Takeda, AbbVie, AstraZeneca, Eli Lilly, Orion, Pfizer, Roche, Sanofi Aventis, UCB, Biogen, GSK, Bristol Myers Squibb and Genentech Inc; payment or honoraria for lectures, presentations, speakers bureaus, manuscript writing or educational events from Ferring, Takeda, Janssen, Nordic Pharma and Dr Falk; participation (Secretary) on the British Society of Gastroenterology IBD Section; participation on the Steering Committee of IBD UK. Charlie W. Lees reports grants from UKRI Future Leaders Fellowship; personal consulting fees from AbbVie, Pfizer, Janssen, Takeda, Galapagos, Fresnius Kabi, Novartis/Sandoz, BMS, and Celltrion; payment or honoraria for lectures, presentations,

For peer review only - http://bmjopen.bmj.com/site/about/guidelines.xhtml

speakers bureaus, manuscript writing or educational events from AbbVie, Pfizer, Janssen, Takeda, Galapagos, Fresnius Kabi, Novartis/Sandoz, BMS, Ferring, Dr Falk, and Celltrion. James O. Lindsay reports grants from AbbVie, and Gilead; personal consulting fees from Allergan, AbbVie, Bristol Myers Squib, Celgene, Cornerstones US, Galapagos, Gilead, GSK, Lilly, MSD UK, Shire UK, Shire International, Ferring UK, Ferring International, Celltrion, Takeda, Pfizer, and Janssen; payment or honoraria for lectures, presentations, speakers bureaus, manuscript writing or educational events from AbbVie, Bristol Myers Squibb, Cornerstones US, Galapagos, Ferring UK, Ferring International, Celltrion, Takeda, Pfizer, and Janssen; support to attend meetings and/or travel from AbbVie, and Janssen. Rebecca H. Maier reports that she is an independent membership on the Trial Steering Committee for the National Institute for Health and Care Research funded ALLEGRO trial. Julian R. Marchesi reports personal consulting fees from EnteroBiotix Limited and Cultech Limited; patent held (without financial gain) on C. *Difficile* therapy (WO2019197836A1), participation (Chair) on the IDMC Board. Naomi McGregor reports partial (10%) salary funding from the Medical Research Council. Rebecca E. McIntyre reports personal salary funding from the Wellcome Sanger Institute. Jasmin Ostermayer reports stock held in Novartis AG. Miles Parkes reports grants from Pfizer and Gilead; personal consulting fees from Galapagos; payment or honoraria for lectures, presentations, speakers bureaus, manuscript writing or educational events from Janssen. Nick Powell reports grants from Bristol-Myers Squibb, Takeda, and Pfizer; consulting fees from AbbVie, Allergan, Astra-Zeneca, Bristol-Myers Squibb, Celgene, Celltrion, Galapagos, GSK, Takeda, and Vifor; payment or honoraria for lectures, presentations, speakers bureaus, manuscript writing or educational events from AbbVie, Bristol-Myers Squibb, Ferring, Galapagos, Janssen, Roche, Pfizer, Takeda, and Tillotts; support for attending meetings and/or travel from AbbVie, Allergan, Celltrion, Janssen, and

For peer review only - http://bmjopen.bmj.com/site/about/guidelines.xhtml

Takeda; participation on a data safety monitoring board or advisory board for AbbVie, Allergan, Astra-Zeneca, Bristol-Myers Squibb, Celgene, Celltrion, Galapagos, GSK, Takeda, and Vifor. Tim Raine reports personal grants from AbbVie; personal consulting fees from AbbVie, Arena, Aslan, AstraZeneca, Boehringer-Ingelheim, BMS, Celgene, Ferring, Galapagos, Gilead, GSK, Heptares, LabGenius, Janssen, Mylan, MSD, Novartis, Pfizer, Roche, Sandoz, Takeda, UCB, and XAP therapeutics; participation on the board of UCB, membership (Chair) of the ECCO Guidelines Committee, membership of the UEG Scientific Committee. Jack Satsangi reports grants from Crohn's and Colitis UK, the Helmsley Charitable Trust, ECCO, the European Commission, CCFA, and Action Medical Research; payment or honoraria for lectures, presentations, speakers bureaus, manuscript writing or educational events from Roche; participation on a Data Safety Monitoring Board or Advisory Board for the MODULATE trial and the TRIBUTE trial; leadership or fiduciary role as the Director of the Royal College of Physicians IBD Registry, and Governing Body Fellow at Green Templeton College. Ally Speight reports personal consulting fees from GSK; payment or honoraria for lectures, presentations, speakers bureaus, manuscript writing or educational events from Falk, and AbbVie; payment of conference fees to attend the British Society of Gastroenterology Annual Conference 2022 from Celltrion; participation on a Data Safety Monitoring Board or Advisory Board for the IBD-RESPONSE study (unpaid), and Abbvie; participation on the British Society of Gastroenterology IBD Section Committee. Christopher J. Stewart reports personal consultancy fees from Astarte Medical; payment or honoraria for lectures, presentations, speakers bureaus, manuscript writing or educational events from Nestle Nutrition Institute. James M. S. Wason reports grants from Intercept; consulting fees from Worg and UCB; payment or honoraria for lectures, presentations, speakers bureaus, manuscript writing or educational events from Janssen; participation on a Data Safety

For peer review only

Monitoring Board or Advisory Board for Roche. Kevin Whelan reports grants from the Helsmsley Charitable Trust, Crohn's and Colitis UK, Almond Board of California, Danone, International Dried Fruit and Nut Council, Medical Research Council, National Institute for Health and care Research; royalty or license payments for Volatile organic compounds in the diagnosis and management of irritable bowel syndrome, and Wiley BDA Advances in Nutrition & Dietetics book series; personal consulting fees from Danone; payment or honoraria for lectures, presentations, speakers bureaus, manuscript writing or educational events from Janssen; support for attending meetings and/or travel from Yakult; participation on a Data Safety Monitoring Board or Advisory Board for the MODULATE trial (unpaid). The remaining authors disclose no conflicts of interest.

For peer review only

REFERENCES

1. Lamb CA, Kennedy NA, Raine T, Hendy PA, Smith PJ, Limdi JK, et al. British Society of Gastroenterology consensus guidelines on the management of inflammatory bowel disease in adults. Gut. 2019;68(Suppl 3):s1-s106.

2. Floyd DN, Langham S, Severac HC, Levesque BG. The economic and quality-of-life burden of Crohn's disease in Europe and the United States, 2000 to 2013: a systematic review. Dig Dis Sci. 2015;60(2):299-312.

3. van der Valk ME, Mangen MJ, Leenders M, Dijkstra G, van Bodegraven AA, Fidder HH, et al. Risk factors of work disability in patients with inflammatory bowel disease--a Dutch nationwide web-based survey: work disability in inflammatory bowel disease. J Crohns Colitis. 2014;8(7):590-7.

4. Jones GR, Lyons M, Plevris N, Jenkinson PW, Bisset C, Burgess C, et al. IBD prevalence in Lothian, Scotland, derived by capture-recapture methodology. Gut. 2019;68(11):1953-60.

5. Hamilton B, Green H, Heerasing N, Hendy P, Moore L, Chanchlani N, et al. Incidence and prevalence of inflammatory bowel disease in Devon, UK. Frontline Gastroenterology. 2021;12(6):461.

6. GBD 2017 Inflammatory Bowel Disease Collaborators. The global, regional, and national burden of inflammatory bowel disease in 195 countries and territories, 1990-2017: a systematic analysis for the Global Burden of Disease Study 2017. Lancet Gastroenterol Hepatol. 2020;5(1):17-30.

7. Ng SC, Shi HY, Hamidi N, Underwood FE, Tang W, Benchimol EI, et al. Worldwide incidence and prevalence of inflammatory bowel disease in the 21st century: a systematic review of population-based studies. Lancet. 2017;390(10114):2769-78.

8. Wyatt NJ, Speight RA, Stewart CJ, Kirby JA, Lamb CA. Targeting Leukocyte Trafficking in Inflammatory Bowel Disease. BioDrugs. 2021;35(5):473-503.

9. Jostins L, Ripke S, Weersma RK, Duerr RH, McGovern DP, Hui KY, et al. Host-microbe interactions have shaped the genetic architecture of inflammatory bowel disease. Nature. 2012;491(7422):119-24.

10. Luo Y, de Lange KM, Jostins L, Moutsianas L, Randall J, Kennedy NA, et al. Exploring the genetic architecture of inflammatory bowel disease by whole-genome sequencing identifies association at ADCY7. Nat Genet. 2017;49(2):186-92.

11. Ananthakrishnan AN, Luo C, Yajnik V, Khalili H, Garber JJ, Stevens BW, et al. Gut Microbiome Function Predicts Response to Anti-integrin Biologic Therapy in Inflammatory Bowel Diseases. Cell Host Microbe. 2017;21(5):603-10 e3.

12. Doherty MK, Ding T, Koumpouras C, Telesco SE, Monast C, Das A, et al. Fecal Microbiota Signatures Are Associated with Response to Ustekinumab Therapy among Crohn's Disease Patients. MBio. 2018;9(2).

13. Routy B, Le Chatelier E, Derosa L, Duong CPM, Alou MT, Daillere R, et al. Gut microbiome influences efficacy of PD-1-based immunotherapy against epithelial tumors. Science. 2018;359(6371):91-7.

14. Matson V, Fessler J, Bao R, Chongsuwat T, Zha Y, Alegre ML, et al. The commensal microbiome is associated with anti-PD-1 efficacy in metastatic melanoma patients. Science. 2018;359(6371):104-8.

15. Gopalakrishnan V, Spencer CN, Nezi L, Reuben A, Andrews MC, Karpinets TV, et al. Gut microbiome modulates response to anti-PD-1 immunotherapy in melanoma patients. Science. 2018;359(6371):97-103.

For peer review only - http://bmjopen.bmj.com/site/about/guidelines.xhtml

16. Lavelle A, Sokol H. Gut microbiota-derived metabolites as key actors in inflammatory bowel disease. Nature Reviews Gastroenterology & Hepatology. 2020;17(4):223-37.

17. Fiorucci S, Carino A, Baldoni M, Santucci L, Costanzi E, Graziosi L, et al. Bile Acid Signaling in Inflammatory Bowel Diseases. Dig Dis Sci. 2021;66(3):674-93.

18. Lee JWJ, Plichta D, Hogstrom L, Borren NZ, Lau H, Gregory SM, et al. Multi-omics reveal microbial determinants impacting responses to biologic therapies in inflammatory bowel disease. Cell Host & Microbe. 2021;29(8):1294-304.e4.

19. Liu J, Fang H, Hong N, Lv C, Zhu Q, Feng Y, et al. Gut Microbiome and Metabonomic Profile Predict Early Remission to Anti-Integrin Therapy in Patients with Moderate to Severe Ulcerative Colitis. Microbiol Spectr. 2023;11(3):e0145723.

20. Plichta DR, Graham DB, Subramanian S, Xavier RJ. Therapeutic Opportunities in Inflammatory Bowel Disease: Mechanistic Dissection of Host-Microbiome Relationships. Cell. 2019;178(5):1041-56.

21. Kennedy NA, Heap GA, Green HD, Hamilton B, Bewshea C, Walker GJ, et al. Predictors of anti-TNF treatment failure in anti-TNF-naive patients with active luminal Crohn's disease: a prospective, multicentre, cohort study. Lancet Gastroenterol Hepatol. 2019;4(5):341-53.

22. Narula N, Peerani F, Meserve J, Kochhar G, Chaudrey K, Hartke J, et al. Vedolizumab for Ulcerative Colitis: Treatment Outcomes from the VICTORY Consortium. Am J Gastroenterol. 2018;113(9):1345.

23. Wils P, Bouhnik Y, Michetti P, Flourie B, Brixi H, Bourrier A, et al. Subcutaneous Ustekinumab Provides Clinical Benefit for Two-Thirds of Patients With Crohn's Disease Refractory to Anti-Tumor Necrosis Factor Agents. Clin Gastroenterol Hepatol. 2016;14(2):242-50 e1-2.

24. Tsai L, Ma C, Dulai PS, Prokop LJ, Eisenstein S, Ramamoorthy SL, et al. Contemporary Risk of Surgery in Patients With Ulcerative Colitis and Crohn’s Disease: A Meta-Analysis of Population-Based Cohorts. Clinical Gastroenterology and Hepatology. 2021;19(10):2031-45.e11.

25. Ghosh N, Premchand P. A UK cost of care model for inflammatory bowel disease. Frontline Gastroenterol. 2015;6(3):169-74.

26. Hart AL, Lomer M, Verjee A, Kemp K, Faiz O, Daly A, et al. What Are the Top 10 Research Questions in the Treatment of Inflammatory Bowel Disease? A Priority Setting Partnership with the James Lind Alliance. J Crohns Colitis. 2017;11(2):204-11.

27. Parkes M, Investigators IBDB. IBD BioResource: an open-access platform of 25 000 patients to accelerate research in Crohn's and Colitis. Gut. 2019;68(9):1537-40.

28. Ding NS, McDonald JAK, Perdones-Montero A, Rees DN, Adegbola SO, Misra R, et al. Metabonomics and the Gut Microbiome Associated With Primary Response to Anti-TNF Therapy in Crohn's Disease. J Crohns Colitis. 2020;14(8):1090-102.

29. Bonder MJ, Kurilshikov A, Tigchelaar EF, Mujagic Z, Imhann F, Vila AV, et al. The effect of host genetics on the gut microbiome. Nat Genet. 2016;48(11):1407-12.

30. Moore CM, Jacobson SA, Fingerlin TE. Power and Sample Size Calculations for Genetic Association Studies in the Presence of Genetic Model Misspecification. Hum Hered. 2019;84(6):256-71.

31. Brion MJ, Shakhbazov K, Visscher PM. Calculating statistical power in Mendelian randomization studies. Int J Epidemiol. 2013;42(5):1497-501.

32. Jairath V, Khanna R, Zou GY, Stitt L, Mosli M, Vandervoort MK, et al. Development of interim patient-reported outcome measures for the assessment of ulcerative colitis disease activity in clinical trials. Aliment Pharmacol Ther. 2015;42(10):1200-10.

For peer review only - http://bmjopen.bmj.com/site/about/guidelines.xhtml

33. Khanna R, Zou G, D'Haens G, Feagan BG, Sandborn WJ, Vandervoort MK, et al. A retrospective analysis: the development of patient reported outcome measures for the assessment of Crohn's disease activity. Aliment Pharmacol Ther. 2015;41(1):77-86.

34. Lewis SJ, Heaton KW. Stool form scale as a useful guide to intestinal transit time. Scand J Gastroenterol. 1997;32(9):920-4.

35. Bodger K, Ormerod C, Shackcloth D, Harrison M, Collaborative IBDC. Development and validation of a rapid, generic measure of disease control from the patient's perspective: the IBD-control questionnaire. Gut. 2014;63(7):1092-102.

36. Cella D, Yount S, Rothrock N, Gershon R, Cook K, Reeve B, et al. The Patient-Reported Outcomes Measurement Information System (PROMIS): progress of an NIH Roadmap cooperative group during its first two years. Med Care. 2007;45(5 Suppl 1):S3-S11.

37. Herdman M, Gudex C, Lloyd A, Janssen M, Kind P, Parkin D, et al. Development and preliminary testing of the new five-level version of EQ-5D (EQ-5D-5L). Qual Life Res. 2011;20(10):1727-36.

38. Janssen MF, Pickard AS, Golicki D, Gudex C, Niewada M, Scalone L, et al. Measurement properties of the EQ-5D-5L compared to the EQ-5D-3L across eight patient groups: a multi-country study. Qual Life Res. 2013;22(7):1717-27.

39. Craig CL, Marshall AL, Sjostrom M, Bauman AE, Booth ML, Ainsworth BE, et al. International physical activity questionnaire: 12-country reliability and validity. Med Sci Sports Exerc. 2003;35(8):1381-95.

40. Lukas C, Landewe R, Sieper J, Dougados M, Davis J, Braun J, et al. Development of an ASAS-endorsed disease activity score (ASDAS) in patients with ankylosing spondylitis. Ann Rheum Dis. 2009;68(1):18-24.

41. Machado PM, Landewe R, Heijde DV, Assessment of SpondyloArthritis international S. Ankylosing Spondylitis Disease Activity Score (ASDAS): 2018 update of the nomenclature for disease activity states. Ann Rheum Dis. 2018;77(10):1539-40.

42. van der Heijde D, Lie E, Kvien TK, Sieper J, Van den Bosch F, Listing J, et al. ASDAS, a highly discriminatory ASAS-endorsed disease activity score in patients with ankylosing spondylitis. Ann Rheum Dis. 2009;68(12):1811-8.

43. Hollis JL, Craig LCA, Whybrow S, Clark H, Kyle JAM, McNeill G. Assessing the relative validity of the Scottish Collaborative Group FFQ for measuring dietary intake in adults. Public Health Nutrition. 2017;20(3):449-55.

44. Collins GS, Reitsma JB, Altman DG, Moons KGM. Transparent reporting of a multivariable prediction model for individual prognosis or diagnosis (TRIPOD): the TRIPOD Statement. BMC Medicine. 2015;13(1):1.

For peer review only - http://bmjopen.bmj.com/site/about/guidelines.xhtml

WORD COUNT

4,972 words excluding words in figures/tables/boxes, contributorship statement, competing interests, funding, acknowledgements and references

For peer review only

For peer review only - http://bmjopen.bmj.com/site/about/guidelines.xhtml

FIGURE LEGENDS

Figure 1. Schematic illustrating the evolving approach to treatment of inflammatory bowel disease, with the aim of improving treatment outcomes through individualised precision-medicine. Current treatment selection is stratified and modified based on diagnosis, disease phenotype, imaging (radiological and endoscopic) and limited clinical biomarkers, such as blood and stool markers of inflammation, drug metabolising enzyme activity, drug levels, and anti-drug antibodies. Precision medicine approaches integrating additional complex multi-omic data with information about environmental factors such as dietary intake, smoking and physical activity levels (the exogenous 'exposome'), may enable individualised treatment selection through predictive modelling. Precision medicine may also help to identify of at-risk populations, predict disease course, reduce unnecessary patient risk and health service costs associated with ineffective pharmacological therapies, and guide non-pharmacological interventions such as dietary modification. Figure created with BioRender.com

Figure 1 abbreviations: c-reactive protein (CRP), thiopurine methyltransferase (TPMT)

Figure 2. Study overview schematic. 1,325 participants with IBD planned to commence an advanced therapy will be recruited, including a nested sub-cohort of 300 CD patients (CD-metaRESPONSE). All participants will collect two stool sample tubes at each study assessment timepoint (baseline, week 14 and week 54). CD-metaRESPONSE participants will be required to collect a third stool sample tube at baseline and week 14. If a participant attends hospital for a face-to-face appointment within the baseline and/or week 14 study assessment window, blood samples will be collected. If a participant attends hospital for a lower gastrointestinal endoscopy at any time during the study period (pre- or post- treatment), biopsy samples will be collected. Participants will complete several questionnaires at each

For peer review only - http://bmjopen.bmj.com/site/about/guidelines.xhtml

assessment time point. For CD-metaRESPONSE participants, additional detailed analyses will be undertaken of metabolic profiles (metabolome) in stool and matched blood plus in-depth dietary assessment (additional elements highlighted in blue boxes). Data generated will be used to perform predictive modelling. Any remaining participant samples will form a large biorepository for use in future research. Figure created with BioRender.com

Figure 2 abbreviations: anti-tumour necrosis factor alpha (anti-TNFα), anti-interleukin (anti-IL), Bristol stool form scale (BSFS), Crohn's disease (CD), formalin-fixed paraffin embedded (FFPE), inflammatory bowel disease (IBD), International Physical Activity Questionnaire (IPAQ), janus kinase inhibitor (JAKi), Joint Pain and Stiffness Score (JPSS), Patient Reported Outcome-2 (PRO-2), Patient Reported Outcomes Measurement Information System (PROMIS), sphingosine-1-phosphate receptor (S1PR), ulcerative colitis (UC).

Figure 3. Flowchart providing overview of study events.

Figure 3 abbreviations: Crohn's disease (CD), inflammatory bowel disease (IBD), Janus kinase inhibitor (JAKi), Mayo Clinic Score (MCS), multidisciplinary team (MDT), Patient Reported Outcome-2 (PRO-2), simple endoscopic score for Crohn's disease (SES-CD), sphingosine-1-phosphate receptor (S1PR), Research Electronic Data Capture (REDCap), ulcerative colitis (UC).

Supplementary Figure 1. Power at different sample sizes for detecting associations in the primary analysis between microbiome variables at baseline and response to treatment at 14 weeks or remission at week 52, using two different plausible effect sizes (d=0.66 and d=0.30). Vertical lines mark the sample size required to achieve 80% power for the four different scenarios.

For peer review only - http://bmjopen.bmj.com/site/about/guidelines.xhtml

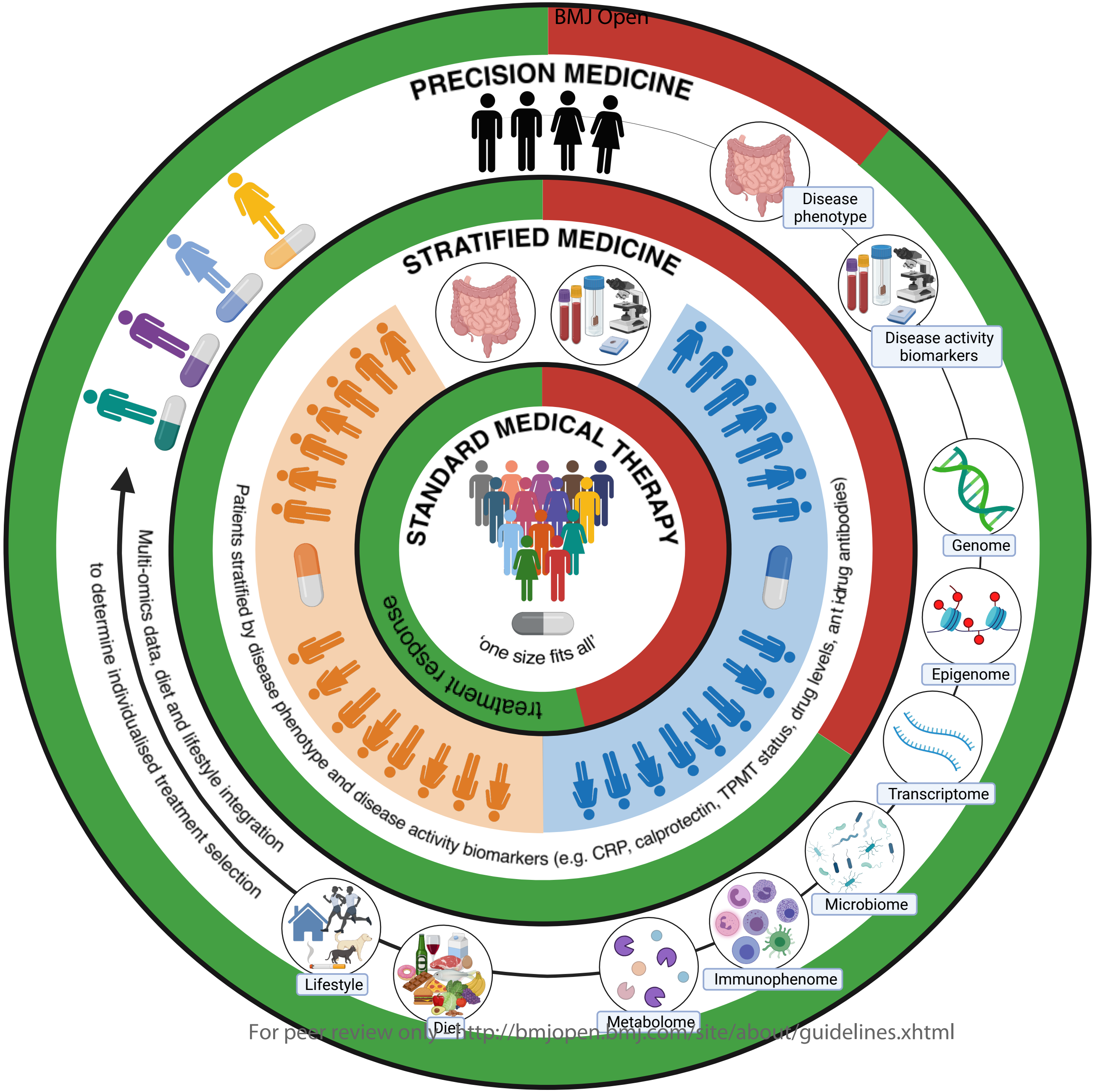
PRECISION MEDICINE
Disease phenotype
Disease activity biomarkers
Genome
Epigenome
Transcriptome
Microbiome
Immunophenome
Metabolome
Diet
Lifestyle
Multi-omics data, diet and lifestyle integration
to determine individualised treatment selection
STRATIFIED MEDICINE
Patients stratified by disease phenotype and disease activity biomarkers (e.g. CRP, calprotectin, TPMT status, drug levels, anti-drug antibodies)
STANDARD MEDICAL THERAPY
'one size fits all'
treatment response

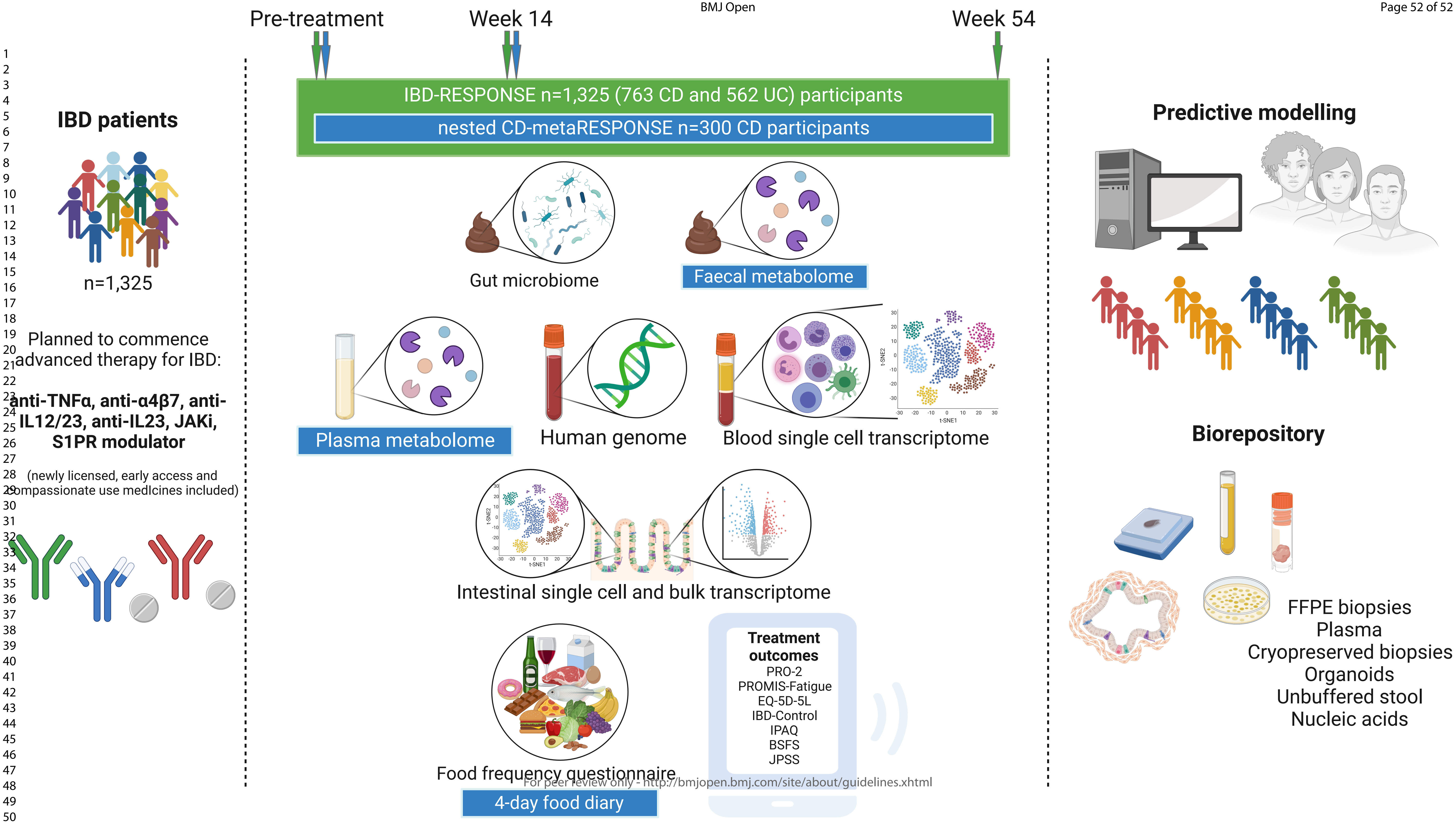
Pre-treatment
Week 14
Week 54
IBD patients
n=1,325
Planned to commence advanced therapy for IBD:
anti-TNFα, anti-α4β7, anti-IL12/23, anti-IL23, JAKi, S1PR modulator
(newly licensed, early access and compassionate use medIcines included)
IBD-RESPONSE n=1,325 (763 CD and 562 UC) participants
nested CD-metaRESPONSE n=300 CD participants
Gut microbiome
Faecal metabolome
Plasma metabolome
Human genome
Blood single cell transcriptome
t-SNE1
t-SNE2
Intestinal single cell and bulk transcriptome
Food frequency questionnaire
4-day food diary
Treatment outcomes
PRO-2
PROMIS-Fatigue
EQ-5D-5L
IBD-Control
IPAQ
BSFS
JPSS
Predictive modelling
Biorepository
FFPE biopsies
Plasma
Cryopreserved biopsies
Organoids
Unbuffered stool
Nucleic acids

Potential participant identified

Where: Face-to-face clinics, telephone consultations, endoscopy lists, infusion suites, MDT meetings, virtual biologics clinics, IBD nurse helpline
Who: ≥16 years old; symptomatic, active luminal IBD; no minimum disease duration
Advanced therapy being commenced: Licensed biologic, JAKi or S1PR modulator (infliximab, adalimumab, vedolizumab, ustekinumab, tofacitinib, filgotinib, upadacitinib, ozanimod) or early access/compassionate use therapy (e.g. risankizumab)
Screening tips:

- UC symptoms: increased stool frequency relative to baseline AND at least one episode of recent rectal bleeding within e.g. last 3-5 days
- CD symptoms of active flare: presence of liquid or very soft stools AND/OR presence of abdominal pain
- Active disease confirmed by clinical test in 16 weeks prior to consent: faecal calprotectin, CRP, endoscopy or (CD only) imaging
- Participants do not need to be naïve to advanced therapies e.g. can previously have received a biologic
- Participants switching between advanced therapies do not need a minimum washout period
- Participants can be receiving or about to start concomitant thiopurine or methotrexate alongside planned advanced therapy
- Participants can be receiving or starting steroids at baseline provided likely to have fully weaned by week 14

↓

Eligibility confirmed

↓

Study information and informed consent given

- Participant Information Sheet (PIS) provided (paper or electronic format)
- Potential participant questions answered by local study team
- REDCap access given to participant to complete informed study consent

↓

Consent verified

Stool sample collection kit given to/posted to participant once valid consent obtained and verified by local study team

↓

Baseline assessment

Ideal: Week 0
Target: Up to 6 weeks prior to commencing advanced therapy
Permitted: Assessment >6 weeks prior to commencing advanced therapy is permitted, e.g. if delayed due to infusion unit capacity

All participants to complete after consent and before starting advanced therapy:

1. Participant questionnaires completed on REDCap
2. Stool sample tubes (n=2) collected and returned using prepaid Royal Mail Safebox™
3. If attending hospital for a face-to-face clinical encounter within assessment window, two tubes (total 20mls) blood collected

CD-metaRESPONSE sub-cohort (in addition to above requirements):

1. Third stool sample tube collected (provided in home stool sample collection kit)
2. Prospective 4-day food diary completed

→ Participants unable or unwilling to complete the PRO-2 questionnaire, not meeting the PRO-2 threshold for clinically active disease, missing stool samples or who do not start treatment with an advanced therapy will be withdrawn from the study

↓

Eligibility re-confirmed

Baseline 4 day PRO-2 (minimum 2 days data) assessed to confirm inclusion criteria for clinically active disease met

↓

Biologic, JAKi or S1PR modulator commenced

WEEK 0

↓

Week 14 assessment

Ideal: Week 14
Target: Week 12 - 16
Permitted: Week 10 - 20

As per baseline assessment (including additional requirements for CD-metaRESPONSE)

↓

Week 54 assessment

Ideal: Week 54
Permitted: Week 48 - 60

All participants:

1. Participant questionnaires completed on REDCap
2. Stool sample tubes (n=2) collected and returned using prepaid Royal Mail Safebox™

If a participant stops treatment, the next planned study assessment should be brought forward and completed as a treatment discontinuation assessment. Where an alternative advanced therapy is then commenced, this discontinuation assessment will serve as the baseline assessment for the next therapy. Further assessments should then be completed at week 14 and 54 after commencing a new advanced therapy as per the study protocol

In addition to the above assessments, if a participant attends hospital for a planned lower gastrointestinal endoscopy at any time during the study period following consent, the following samples/data will be collected: up to 12 research biopsies, patient-reported 1-day PRO-2 score, endoscopic assessment of disease activity data (SES-CD/MCS endoscopic subscore)

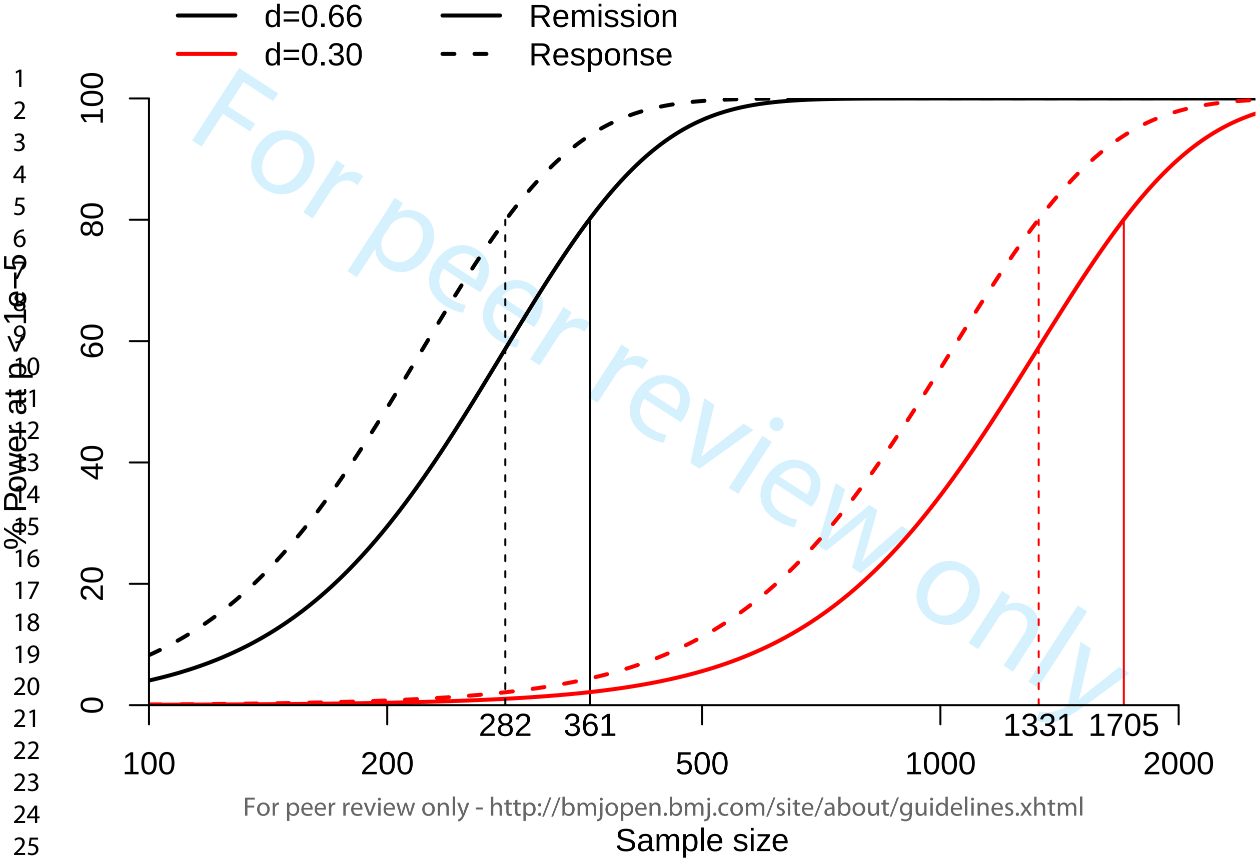

For peer review only
